# Dual suppression of stemness and redox adaptation in glioblastoma through filaggrin upregulation by an abiraterone-based HDAC inhibitor

**DOI:** 10.1186/s12929-026-01241-2

**Published:** 2026-04-06

**Authors:** Hoang Yen Tran, Ram Sharma, Hong-Yi Lin, Tzu-Yi Yeh, Chih-Jie Shen, Tsung-I. Hsu, Jing-Ping Liou

**Affiliations:** 1 School of Pharmacy, College of Pharmacy, Taipei Medical University, Taipei, 110 Taiwan; 2 Department of Pharmacology and Clinical Pharmacy, Faculty of Pharmacy, Can Tho University of Medicine and Pharmacy, Can Tho City, 900000 Vietnam; 3 Taipei Neuroscience Institute, New Taipei City, 235 Taiwan; 4 Taiwan Brain Disease Foundation, Taipei, 100 Taiwan; 5 TMU Research Center of Neuroscience, Taipei Medical University, Taipei, 235 Taiwan; 6 TMU Research Center for Drug Discovery, Taipei Medical University, Taipei, 110 Taiwan; 7 TMU Research Center of Cancer Translational Medicine, Taipei, 110 Taiwan; 8 Ph.D. Program in Medical Neuroscience, College of Medical Science and Technology, Taipei Medical University and National Health Research Institutes, Taipei, 235 Taiwan; 9 International Master Program in Medical Neuroscience, College of Medical Science and Technology, Taipei Medical University, Taipei, 235 Taiwan; 10 Ph.D. Program in Drug Discovery and Development Industry, College of Pharmacy, Taipei Medical University, Taipei, 110 Taiwan

**Keywords:** Glioblastoma, Glioma stem cells, MGMT, Reactive oxygen species, Filaggrin

## Abstract

**Background:**

Temozolomide (TMZ) resistance in glioblastoma (GBM) remains a critical barrier to treatment success, driven by O^6^-methylguanine-DNA methyltransferase (MGMT) overexpression, glioma stem cell (GSC) persistence, and redox adaptation.

**Methods:**

We developed cp8, a first-in-class abiraterone-based histone deacetylase (HDAC) inhibitor, to simultaneously target these resistance mechanisms. The orthotopic mouse models were used to evaluate the efficacy of cp8 compared to SAHA (vorinostat). The mouse survival period was recorded, and the tumor growth was monitored using the IVIS imaging system.

**Results:**

Cp8 demonstrated approximately tenfold greater potency than SAHA, with IC₅₀ values ≤ 3 µM against TMZ-resistant GBM cells (compared with ≥ 30 µM for SAHA). Transcriptomic analysis revealed a unique ability of cp8 to upregulate filaggrin (FLG), a structural protein whose expression correlated with improved patient survival in TCGA and CGGA datasets (p = 0.001). Functional studies showed that FLG knockdown increased GSC-associated markers (Oct4, 2.1-fold; SOX2, 1.8-fold) and enhanced TMZ resistance, whereas cp8 treatment reduced MGMT protein expression by 68% and significantly decreased glioma sphere size by 54% (p < 0.01). In orthotopic models, cp8 extended median survival to 59 days compared with 34 days for controls (p < 0.001) and 49 days for SAHA (p < 0.01), while reducing tumor volume by 72% (p < 0.001) without systemic toxicity. Mechanistically, dual inhibition of HDAC6 and CYP17A1 by cp8 disrupted redox homeostasis and stemness-associated pathways, leading to altered ROS metabolism, reduced MGMT expression, and attenuation of GSC-driven tumor growth while restoring FLG-mediated tumor suppression.

**Conclusion:**

This study establishes FLG as a novel therapeutic target in GBM and validates the suppressive efficacy of cp8 on the characteristics of TMZ resistance, highlighting the translational potential as a multitargeted therapy against TMZ-resistant GBM.

**Supplementary Information:**

The online version contains supplementary material available at 10.1186/s12929-026-01241-2.

## Introduction

Despite revolutionary advancements in cancer treatment, drug resistance remains a significant obstacle, enabling tumors to evade therapy and continue growing. This challenge is especially evident in glioblastoma (GBM), an aggressive and lethal primary brain cancer, where resistance to standard treatments like temozolomide (TMZ) and targeted therapies severely limits patient outcomes [1, 2]. Unfortunately, more than 50% of GBM patients exhibit poor prognosis due to acquiring resistance to TMZ [3, 4]. Expression of O^6^-methylguanine-DNA methyltransferase (MGMT), removing TMZ-induced alkylation from nucleotides, is strongly implicated as the primary mechanism driving TMZ resistance in GBM [5, 6]. In addition, TMZ resistance in GBM is not solely due to MGMT but also involves the glioma stem cell (GSC) enrichment, disrupted reactive oxygen species accumulation, hyperactive DNA repair, increased drug efflux, and enhanced cell survival mechanisms [3, 7–10].

Filaggrin (FLG) is a key structural protein that is mainly found in the epidermis and is essential for healthy skin hydration and barrier [11, 12]. By aggregating keratin filaments, it creates a stronger epidermal barrier that protects against environmental factors, pathogens, and dehydration [12]. Mutations or deficiencies in the FLG gene have been associated with various dermatological disorders, leading to weakened skin barriers and increased susceptibility to allergens and infections [12, 13]. Recent studies have indicated the pivotal involvement of FLG mutations in cancer biology and clinical outcomes, including skin cancer [14], bladder urothelial carcinoma [15], cervical cancer [16], and human papillomavirus-related cancer [17]. However, the significance of FLG in GBM is still under investigation, and current research lacks evidence of its influence on GBM development.

Glioma stem cells (GSCs) are a subset of GBM tumor cells that play a pivotal role in therapy resistance and tumor recurrence [18]. The self-renewal and adaptability to harsh conditions allow these cells to survive chemotherapy and restore the tumor [19, 20]. Their ability to escape apoptosis, sustain MGMT expression, and alter the tumor microenvironment makes them key drivers of tumorigenic initiation, progression, TMZ resistance, and GBM recurrence [21]. Several GSC markers, including Oct4, SOX-2, Bmi-1, and CD133, contribute to TMZ resistance by promoting stemness, DNA repair, and therapy evasion. Studies have indicated that radiotherapy and chemotherapy are less effective against cancer stem cells than against other tumor cells [22, 23], and TMZ treatment may increase the number of GSCs [24, 25], potentially resulting in worse patient outcomes. Moreover, reactive oxygen species (ROS) and redox homeostasis serve crucial roles in mediating therapeutic resistance in TMZ-resistant GBM. Strategies that disrupt redox balance have demonstrated potential in overcoming this resistance. The discoveries emphasize the pressing need for novel therapeutic strategies targeting MGMT, ROS expression, and GSC survival simultaneously.

Our prior findings suggested a critical involvement for CYP17A1 in GBM progression [26]. Abiraterone, a CYP17A1 inhibitor, demonstrated promise in suppressing GBM by targeting both tumor survival mechanisms and the immune microenvironment. This compound was able to inhibit the survival of GBM cells by disrupting SAR1-mediated endoplasmic reticulum function and redox balance, leading to cellular stress and apoptosis in GBM cells [27]. In parallel, histone deacetylase (HDAC) inhibitors represent one of the most intensively investigated areas in antitumor scaffold development due to their capacity to induce the acetylation of histone and nonhistone proteins, thereby modulating gene expression and influencing various cellular pathways critical to cancer progression. By inhibiting HDAC6, induction of DNA damage and oxidative stress can sensitize GBM cells to TMZ, leading to significant GBM cell survival reduction [28].

Building on these findings, this study aims to synergize the antitumor properties of abiraterone with the epigenetic modulation capabilities of HDAC inhibitors to develop novel hybrid chemical architectures with enhanced therapeutic potential against GBM, especially to overcome TMZ resistance by attenuating GSC enrichment and MGMT expression and malfunctioning ROS. This innovative design strategy integrates the antiproliferative effects of abiraterone with the epigenetic regulatory mechanisms of HDAC inhibition, synthesizing a dual-action therapeutic approach for TMZ-resistant GBM. More importantly, our research will target FLG to explore the potential of our novel abiraterone derivatives in regulating FLG for the discovery and development of future anti-GBM therapies.

## Methods

### Cell line and cell culture

Both human and mouse glioma cell lines were included to assess cross-species conservation of compound efficacy; however, key comparative analyses were performed within species-matched pairs. Glioma cells, T98G, A172, U87MG, U87MG-luc, and CT-2A, were purchased from American Type Culture Collection (Manassas, VA, USA). Pt#3 (patient-derived GBM cell) and P1S (patient-derived TMZ-resistant cell) were generated as previously described [29–31]. In addition, a detailed description of the derivation source, selection steps, TMZ exposure and MGMT status, and mycoplasma testing of Pt#3, Pt#3-R, and P1S cells is shown in Table S1, Fig. S1, and Fig. S12. Cells were cultivated in complete Dulbecco’s Modification of Eagle’s Medium 1X (DMEM) (Corning, Mediatech, Inc., VA, USA), supplemented with 10% (*v/v*) fetal bovine serum and 100 µg/mL penicillin/streptomycin, and incubated at 37 °C and 5% CO_2_. Pt#3-R, CT-2A-R, and P1S were established and cultured in complete DMEM with 50 μM of TMZ (MilliporeSigma Corporate, St. Louis, MO, USA) or 100 μM of TMZ (U87MG-R). Mycoplasma contamination was monitored every two months (provided in Method S1).

### Stem-like cell culture

DMEM/F12 (1:1, *v/v*) containing recombinant human FGF-basic and recombinant human EGF (both at 20 ng/mL), and N2 supplement was used as the sphere culture medium. All components were purchased from Life Technologies Corporation, NY 14072, USA.

3 × 10^5^ GBM cells were seeded into 6-cm dishes coated with poly(2-hydroxyethyl methacrylate) (Santa Cruz Biotechnology, Inc.) and incubated at 37 °C with 5% CO₂. Fresh medium was added every 2 days. Once the spheres reached a certain volume, they were resuspended in a new medium. After 24 h of reseeding, spheres were treated with the potential compound for 72 h and harvested for western blotting and immunofluorescence staining.

The morphology of the spheres was photographed daily, and their size was quantified using ImageJ software (National Institutes of Health).

### HDAC6 knockout

HDAC6 knockout U87MG cells were generated and provided by Biotools Co., Ltd (New Taipei City, Taiwan) using the CRISPR-Cas9 approach [28].

### Filaggrin (FLG) knockdown and overexpression

FLG small interfering RNA (siRNA) was purchased from Dharmacon Inc. (Lafayette, CO, USA). T98G was transfected with non-target siRNA (Eurogentec, Liège, Belgium) and FLG siRNA using Lipofectamine® RNAiMAX reagent (Invitrogen, Thermo Fisher Scientific) for 72 h, then treated with the potential compound at the indicated concentrations.

FLG overexpression was initially performed through the CRISPR-Cas9 method to insert the FLG promoter sequence into plasmid #99,698 (pAAV-SCP1-dSa VPR mini.-2X snRP-1 BsaI gRNA), a kind gift from Prof. Chih-Hao Yang (Taipei Medical University), based on the prior protocol [32]. FLG-carrying plasmid cloning was conducted as in the previous procedure [33]. FLG-ligated plasmids were transfected into T98G cells using PolyJet™ reagent (SignaGen® Laboratories).

qPCR and western blotting were used to validate the FLG knockdown and overexpression efficiency.

### Cell viability assay

Two thousand cells were seeded into each well of 96-well plates and treated with abiraterone-derived compounds for 72 h. The mixture of cell counting kit-8 (CCK8) reagent (TargetMOI, MA, USA) and DMEM (1:10) was prepared and added to each well for 60 min. The absorbance was measured using an iMark Microplate Reader (BIO-RAD) at 450 nm.

### MTT assay

Cells were seeded onto 24-well culture plates at a density of 5 × 10^4^ cells/well. After incubating for 24 h, cells were exposed to various doses of the indicated compound. Following a 72-h period, 3-(4,5-dimethylthiazol-2-yl)-2,5-diphenyltetrazolium bromide (MTT) reagent (0.5 mg/mL, Sigma Aldrich) in fresh DMEM was added and incubated for 50 min at 37 °C. The MTT medium was then removed, and the formazan crystals were dissolved in 300 μL of DMSO. The absorbance of the DMSO extracts was read at 570 nm with an iMark Microplate Reader (BIO-RAD).

### Caspase-3/7 detection

The culture medium was collected after treatment. An equal volume of caspase-3/7 reagent from the assay kit (Promega, WI, USA) was added to each well of the 96-well plate containing the culture medium. The plate was incubated at room temperature (RT) for 30 min in the dark. The Caspase-Glo® 3/7 program was used to measure the luminescence signals.

### RNA sequencing analysis

Pt#3-R and siFLG Pt#3 cells were treated with the indicated compounds for 72 h. After adding QIAzol lysis reagent (QIAGEN), samples were subjected to Biotools for RNA sequencing. Ingenuity Pathway Analysis (IPA; QIAGEN, Redwood City, CA, USA; 2020—2025 releases) was used for pathway and upstream regulator analyses.

### Colony formation assay

One thousand Pt#3 cells were seeded into each well of 6-well plates. After 24 h, cells were treated with the indicated drugs at various concentrations, with treatments repeated every 3 days during the media change with fresh DMEM. On day 14, cell colonies were stained with 0.05% crystal violet overnight, then rinsed with water and imaged using a scanner (EPSON perfection V600 Photo). The number of colonies was quantitatively analyzed using ImageJ.

### Apoptosis analysis

After 72 h of treatment, cells were stained with an Annexin V/ FITC Apoptosis Detection kit (Thermo Fisher Scientific) for 20 min at RT in the dark. The stained cells were detected by flow cytometric analysis and Guava software (MilliporeSigma Corporate).

### MitoSOX and CellROX assays

MitoSOX™ Red mitochondrial superoxide indicator (Thermo Fisher Scientific) was used to label mitochondrial ROS. Live cell imaging was conducted using ImageXpress® Pico Automated Cell Imaging System (Molecular Devices, San Jose, CA, USA).

Cellular ROS detection by flow cytometry employed a CellROX assay with Dihydrorhodamine 123 (Thermo Fisher Scientific) labelling.

### qPCR

Total cell RNA extraction and purification were carried out using an RNA extraction kit (Zymo Research, Irvine, CA, USA) according to the manufacturer's protocol. RNA concentration and quality measurements were obtained using a NanoDrop 2000 Spectrophotometer (Thermo Scientific). cDNA was synthesized using PrimeScript™ RT Reagent kit (TaKaRa Bio Inc., Japan). mRNA transcripts were quantified by polymerase chain reaction (qPCR) assay using SYBR Green reagent (Promega, WI, USA) and CFX Duet Real-Time PCR System (BIO-RAD). GAPDH served as an internal control for the normalization of gene expression. The primer sequences are provided in Table S2.

### Western blotting

Detailed procedure for sample preparation and western blotting was performed as previously reported [26, 28, 34]. After treatment for the indicated time, cells were harvested, lysed in RIPA lysis buffer (Millipore Corp., Burlington, MA, USA), and centrifuged at 12,000 rpm for 15 min at 4 °C. The supernatant was collected, and protein concentration was determined using a BCA assay. Proteins were separated via SDS-PAGE, transferred to PVDF membranes, and blocked with a blocking buffer (Genestar Biotechnology Co. Ltd) for 10 min. Membranes were incubated overnight at 4 °C with primary antibodies (provided in Table S3), followed by HRP-conjugated secondary antibodies for 1 h at RT. Protein detection was performed using an enhanced chemiluminescence kit (MilliporeSigma Corporate) and visualized with the ChemiDoc™ Touch Imaging System (BIO-RAD).

Western blot images were quantified using ImageJ. Images were converted to grayscale, and integrated density was measured for each band using a fixed-size rectangular region of interest (ROI); background signal was measured using the same ROI placed in an adjacent blank area and subtracted from each band. Quantified data were plotted using GraphPad Prism, and statistical significance was evaluated by one-way ANOVA, with p < 0.05 considered statistically significant.

### Immunofluorescence staining (IF)

After treatment, GBM stem-like cells were fixed with 4% paraformaldehyde for 20 min, then rinsed with phosphate-buffered saline (PBS), followed by permeabilization with 0.3% Triton X-100 in 2% milk. After blocking with 10% goat serum in PBS for 1 h at RT, cells were incubated with the primary antibody, anti-Oct4 (1:100 dilution, GeneTex, GTX100622) or anti-SOX-2 (1:100 dilution, GeneTex, GTX101507), at 4 °C overnight. On the next day, stem-like cells were washed with water and then treated with anti-mouse IgG Alexa Fluor™ 488 donkey (1:100 dilution, Invitrogen, Eugene, US) or anti-rabbit IgG Alexa Fluor™ 594 donkey (1:100 dilution, Invitrogen, Eugene, US) for 1 h at RT, followed by Hoechst 33342 (1:20,000 dilution, Thermo Fisher Scientific) nuclei staining for 10 min at RT. Finally, slides were mounted with ProLong™ Glass Antifade Mountant with NucBlue™ Stain (#P36981, Thermo Fisher Scientific), and fluorescent images were captured using the PICO system.

### Immunohistochemical staining (IHC)

Tissue arrays of GBM patients were purchased from US Biomax Inc. (Derwood, MD, USA). Anti-Oct4 (1:100 dilution, GeneTex, GTX100622), anti-SOX-2 (1:100 dilution, GeneTex, GTX101507), and anti-FLG (1:200 dilution, Antibodies, A307872) were utilized for IHC analysis. Briefly, patient specimens and mouse brain tissue samples from in vivo experiments were consecutively cut with a thickness of 5 μm and subjected to IHC staining. Section deparaffinization was subsequently performed using xylene and rehydrated in graded alcohols (100%, 95%, 80%, 70%, and 50%). Sample antigen retrieval was performed in a pH 6.8 citrate buffer at 95 °C for 30 min. Endogenous peroxidase activity was blocked with 0.3% hydrogen peroxide in methanol for 10 min. Sections were then rinsed with PBS and incubated with primary antibodies at 4 °C overnight, followed by a one-hour incubation in secondary HRP-conjugated antibodies at RT. Hematoxylin counterstaining was used for cell nuclei visualization, and DAB (Agilent Technologies, Singapore) served as a substrate. Tissue slides were finally photographed and quantified with the PICO system. The *H-*score was analyzed by two experienced pathologists.

### TUNEL staining

TUNEL staining was performed on paraffin-embedded sections using a commercial kit (Abcam) with Proteinase K treatment and DAB detection, followed by methyl green counterstaining. The detailed procedure was provided in Method S2.

### Animal experiments

Animal experiments were approved by the Institutional Animal Care and Use Committee of Taipei Medical University (LAC-2023–0022, LAC-2023–0047, LAC2025-0232, SHLAC2023-0081, and SHLAC2023-0096). Male C57BL/6 (IMSR_JAX:000664), *NOD.CB17-Prkdc*^*scid*^*/NCrCrl* mice, and Sprague–Dawley rats were purchased from BioLASCO Taiwan Co., Ltd. (Taipei, Taiwan) and randomly divided into groups.

#### In vivo model to assess the tolerability and toxicity 

C57BL/6 mice were randomly divided into 6 groups and intraperitoneally (i.p.) administered dimethyl sulfoxide (DMSO) (control group) or the potential compound (treatment groups) with increasing doses three times per week. Mice were closely monitored for any abnormalities such as behavior, daily activity, eating, drinking, fur, fecal and urinary excretion. Afterwards, mice were sacrificed, and blood samples were collected for biochemical analysis. The life expectancy of mice was recorded, the mice body weight was observed during the experiment, and the mice blood was collected for biochemical testing.

#### Allograft glioma model

CT-2A cells (5 × 10^4^ in 4 μL of DMEM per mouse) were orthotopically injected into the brains of C57BL/6 mice at a depth of 3 mm. Starting on day 11, mice were i.p. exposed to DMSO or the potential compound two times a week.

#### Xenograft GBM models

Luciferase-expressed U87MG cells (3 × 10^5^ in 4 μL of DMEM per mouse) were implanted into *NOD.CB17-Prkdc*^*scid*^*/NCrCrl* mice brains at 3 mm deep. After 10 days, twice-weekly i.p. injections were performed to administer either the potential compound or SAHA to the experimental mice.

Pt#3-R cells (2 × 10^5^ in 4 μL of DMEM per mouse) were injected into *NOD.CB17-Prkdc*^*scid*^*/NCrCrl* mice brains at 3 mm deep. Subsequently, from day 9 onward, twice-weekly i.p. injections were performed to deliver the potential compound and/or TMZ or DMSO to the experimental mice.

#### Xenograft orthotopic patient-derived GSC model

Pt#3 stem-like cells were cultured as described above. 15-day Pt#3 spheroids were harvested for the experiment. 10^5^ stem-like cells were inoculated into *NOD.CB17-Prkdc*^*scid*^*/NCrCrl* mouse brains. Commencing on day 9, mice were treated i.p. with either DMSO or the potential compound twice weekly.

The mice survival periods were recorded, the tumor growth was monitored using the IVIS imaging system, and the mice body weight was taken note during the experiment. Blood was taken from the mice for biochemistry tests, and the mouse brain, liver, and kidney tissues were subjected to hematoxylin and eosin (H&E) and IHC staining after being sacrificed.

#### In vivo* pharmacokinetic study*

The dosing solution was prepared by dissolving cp8 in a vehicle consisting of DMA, PEG400, and Tween 80 (3:6:1, v/v/v). Male Sprague–Dawley rats (7 weeks old) were administered a single dose of 2 mg/kg cp8 via a jugular vein cannula.

Following administration, plasma and brain samples were harvested at 5, 15, and 30 min, and at 1, 2, and 4 h post-dose (n = 4 per time point). To eliminate residual blood from the cerebral vasculature, animals were transcardially perfused with PBS prior to brain excision.

Plasma was isolated by centrifugation at 5,000 rpm for 10 min at 4℃ and stored at -80 °C until analysis. For sample extraction, 50 μL of thawed plasma was spiked with finasteride (internal standard) and then mixed with 200 μL of methyl tert-butyl ether (MTBE). The mixture was vortexed for 1 min, sonicated for 5 min, and centrifuged at 12,000 rpm for 10 min at 4℃. The resulting supernatant was collected for UPLC-MS/MS analysis. For brain tissues, samples were homogenized in four volumes of PBS *(w/v)* using a probe-type homogenizer. After centrifugation at 8,000 rpm for 10 min, 100 μL of the supernatant was extracted with 400 μL of MTBE following the same procedure described for plasma.

Quantitative analysis was performed using a Waters Xevo TQ-XS triple quadrupole mass spectrometer coupled with a UPLC system. The analytes were detected in positive electrospray ionization (ESI +) mode using multiple reaction monitoring. The specific mass transition monitored for cp8 was *m/z* 524.35 → 332.23. Optimized mass spectrometric parameters included a collision energy of 28 eV and a cone voltage of 40 V. The desolvation temperature was maintained at 350 °C, with desolvation and collision gas flows set at 650 L/h and 25 L/h, respectively. All bioanalytical data were processed, and non-compartmental pharmacokinetic parameters were subsequently calculated using Phoenix WinNonlin software (Version 8.x, Certara, Princeton, NJ, USA).

### Statistics

Data are presented as mean ± standard error of the mean (SEM) from three independent experiments. Two-group comparisons used an unpaired Student t-test, while one-way ANOVA was used for comparisons of more than two groups in GraphPad Prism 10.0.2 (GraphPad Software Inc.). The log-rank test was applied to compare survival analyses. *P* < 0.05 was considered a statistically significant difference.

## Results

### Chemical synthesis and the determination of the most potent abiraterone-derived compound to inhibit GBM cell growth

**Fig. 1:**
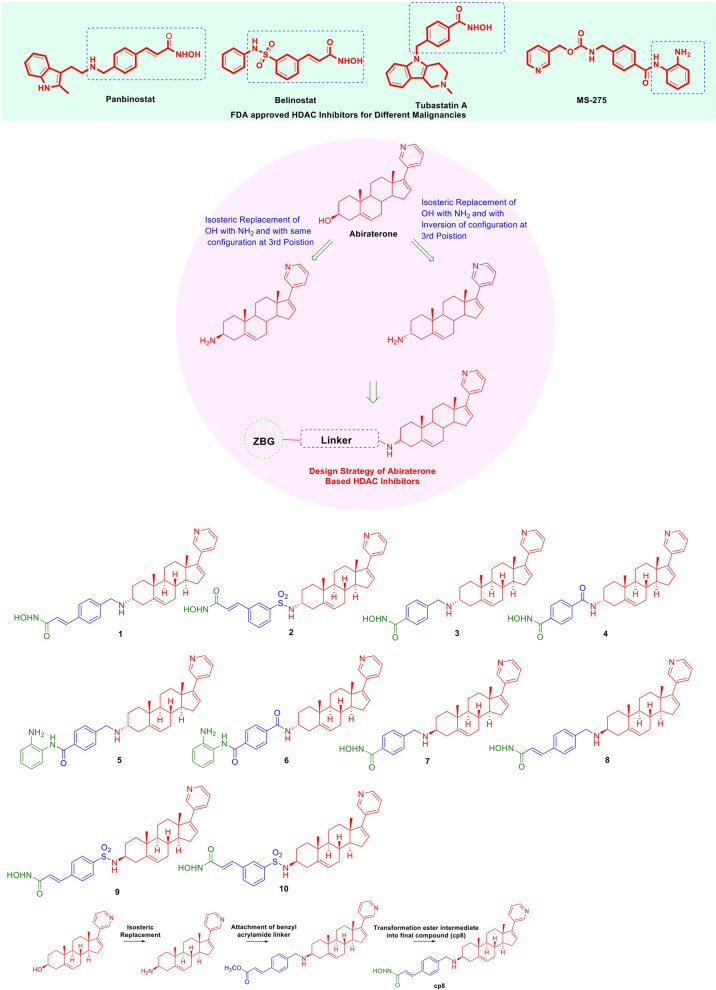
Design strategy and structural framework of abiraterone-based HDAC inhibitors

**Fig. 2:**
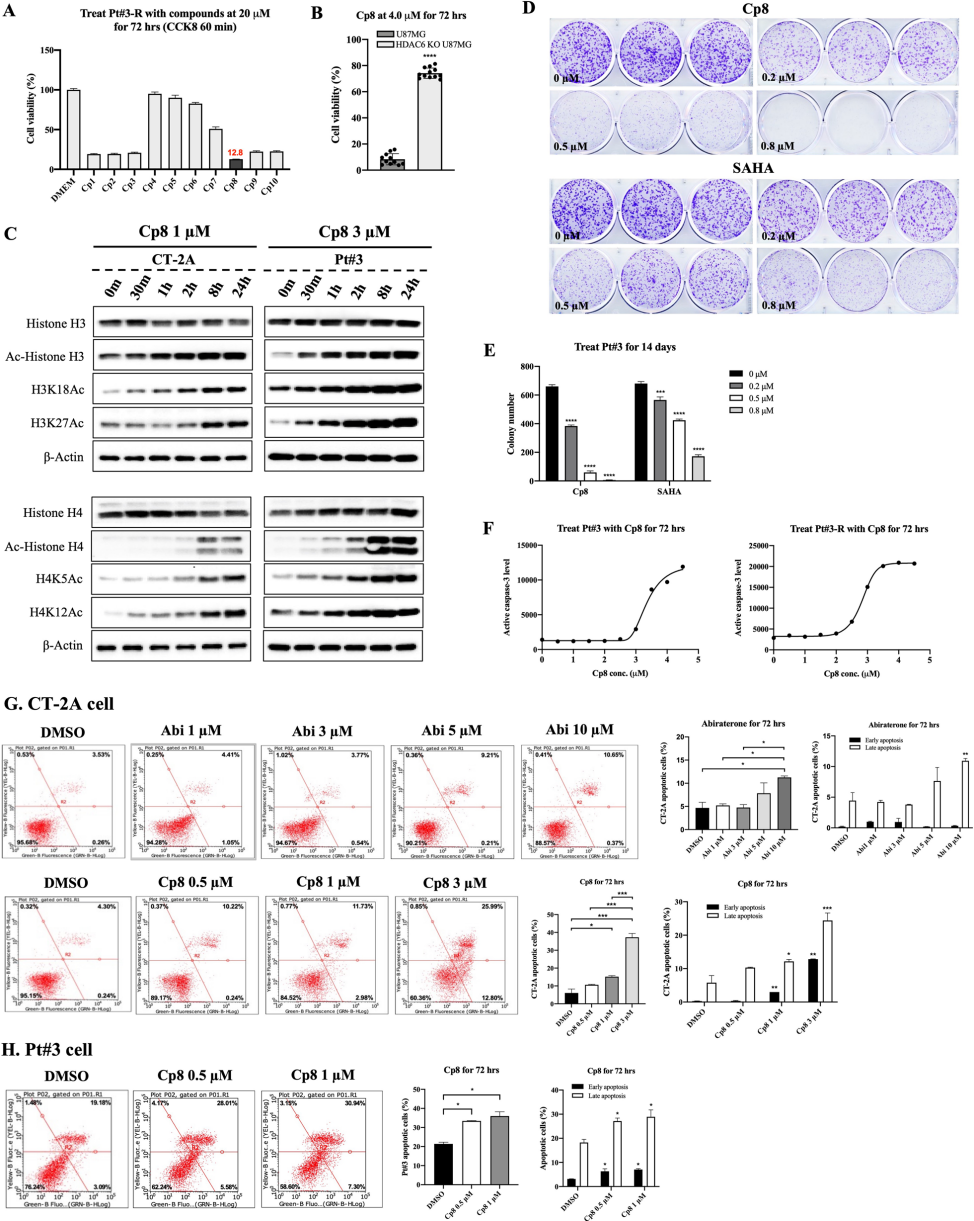
Drug screening and GBM cell proliferation inhibitory effect of cp8. **A** Cell growth inhibitory activity of the synthetic compounds on TMZ-resistant GBM cell, Pt#3-R at 20 µM. **B** Cell viability of wild-type U87MG and HDAC6-knockout U87MG when the cells were treated with cp8 at 4 µM for 72 h. **C** HDAC-inhibitory activity of cp8 on histone H3 and H4 acetylation, respectively. **D**, **E** The cell proliferative inhibitory ability of cp8 on Pt#3 cells at multiple doses was evaluated utilizing the colony formation assay compared to SAHA, and the number of colonies in each group, respectively. **F** Active caspase 3 levels were determined when GBM cells were treated with SAHA for 72 h. **G**, **H** Cp8-treated GBM cell population, including apoptotic cells, was detected and compared to abi-treated groups by Annexin V/PI staining, followed by flow cytometry analysis. Mean ± S.E.M of triplicate experiments. *, P < 0.05; **, P < 0.01; ***, P < 0.001; ****, P < 0.0001

To generate the potential abiraterone derivatives, we used an HDAC inhibitory framework in which abiraterone was used as a surface recogintion part (SRP) unit (Fig. 1, Scheme S1–S4). We demonstrated that compounds incorporating sulfonyl, benzyl acrylamide, or benzyl linkers within their structural architecture, particularly those exhibiting an alpha–beta configuration at the 3-position of abiraterone, displayed pronounced inhibitory activity, reducing cell viability to below 50%. The data from the cell viability assay on Pt#3-R, a TMZ-resistant GBM cell line, indicated that the most powerful Pt#3-R cell growth inhibition at 20 µM was observed in compound 8 (cp8), characterized by an N-benzyl acrylamide linker and a β configuration at the 3-position, with cell viability of 12.8% (Fig. 2A). The remaining compounds, with linkers including an N-benzyl acrylamide linker with an inverted configuration (compound cp1), an N-sulfonyl linker with both identical and inverted configurations (compounds cp2, cp9, and cp10), an N-benzoyl linker (compound cp4), a benzamide linker (compounds cp5 and cp6), an N-benzyl linker with a β configuration (compound cp7), or an N-benzyl linker but with an α configuration at the 3-position (compound cp3), showed a lower ability to inhibit GBM cell growth than cp8 (Fig. 2A). This underscores the significance of the β configuration for the potent anti-GBM efficacy of cp8.

To broaden the applicability of our findings, we have added more glioma cell lines to the experiments. For the drug screening, we tested our compound series on CT-2A, U87MG, and P1S cell lines in addition to the previously used model. The results from these models were consistent with our original finding, confirming the reproducibility of our observation (data are provided in Table S4).

Furthermore, IC50 values of cp8 against multiple human and murine wild-type and TMZ-resistant GBM cells were examined and compared with those of abiraterone and TMZ. Species-matched pairs U87MG/U87MG-R, Pt#3/Pt#3-R, CT-2A/CT-2A-R) were used for primary comparative analyses, while additional resistant lines such as T98G and P1S were included to assess reproducibility and cross-species generalizability. Cp8 stood out due to its prominent GBM cell growth inhibitory activity, with IC50 values of less than or equal to 3 μM observed in TMZ-resistant cells, including MGMT-positive GBM cells (T98G and P1S) (Table 1 and Fig. S2). These results demonstrated that cp8 has a pronounced cytotoxicity on GBM cell growth and shows the potential to suppress GBM.

**Table 1:** IC50 values of cp8 (72-h exposure) against multiple GBM cells compared to abiraterone and TMZ

	Species	GBM cell	Cp8 (μM)	Abiraterone (μM)	TMZ (μM)
Wild type cell line	Human	A172	1.13 ± 0.07	50.05 ± 3.92	ND
		U87MG	1.99 ± 0.16	21.35 ± 2.48	230
		Pt#3	1.88 ± 0.11	19.52 ± 0.45	250
	Murine	CT-2A	0.74 ± 0.04	15.74 ± 0.77	ND
TMZ-resistant cell line	Human	T98G (MGMT +)	1.88 ± 0.88	7.60 ± 0.2	940
		U87MG-R (MGMT-)	3.00 ± 0.12	2.44 ± 3.72	ND
		Pt#3-R (MGMT-)	2.77 ± 0.07	20.87 ± 0.68	550
		P1S (MGMT +)	2.139 ± 0.09	ND	ND
	Murine	CT-2A-R (MGMT-)	1.34 ± 0.1	7.86 ± 0.39	600

*IC50* half-maximal inhibitory concentration, *cp8* compound 8, *GBM* glioblastoma, *TMZ* temozolomide, *MGMT* O^6^-methylguanine-DNA methyltransferase, *ND* not determined

Parallelly, the HDAC-inhibitory activity of cp8 was also validated. As shown in Fig. 2B, the percentage of alive HDAC6-knocked out U87MG after 72-h treatment with cp8 at 4 µM was significantly high, approaching 75%, whereas the cell viability of U87MG was nearly 10%, indicating that HDAC6 is involved in the pathway of cp8-mediated GBM suppression. Additionally, a substantial rise of histone H3 and H4 acetylation was detected when CT-2A and Pt#3 cells were treated with cp8 for 24 h (Fig. 2C and Fig. S3). The expression of histone H3 and histone H4 remained unchanged, whereas there were remarkable increases in the levels of acetylated histone H3 (Ac-histone H3) and acetylated histone H4 (Ac-histone H4). Also, the expression of H3K18Ac, H3K23Ac, H3K27Ac, H4K5Ac, H4K12Ac, and H4K20Ac, was significantly upregulated by cp8 over time (Fig. S3).

In the colony formation assay, SAHA, a well-known HDAC inhibitor, was utilized as the reference drug. The number of colonies was inversely proportional to the concentrations of cp8, dropping to 6 colonies at 0.8 μM from 660 colonies of the control group, whereas at the same doses, SAHA showed a weaker potency in inhibiting colony formation than cp8 (Fig. 2D, E). These findings indicated that cp8 is capable of suppressing GBM cell proliferation, and its effect outstands that of SAHA. Besides, treatment with cp8 induced GBM cell death through the apoptosis pathway, as evidenced by a significant increase in active caspase 3 level (Fig. 2F) and the population of apoptotic GBM cells (Fig. 2G, H) in a dose-dependent manner. Particularly, cp8 showed a stronger apoptosis-inducing effect on CT-2A cells than abiraterone at the same dose of 3 µM. Taken together, cp8 effectively inhibited GBM cell growth and proliferation and triggered apoptosis in a dose-related manner. The findings implied it may be a promising treatment option for GBM.

### Compound 8 strengthens the FLG gene, leading to the attenuation of GBM

**Fig. 3:**
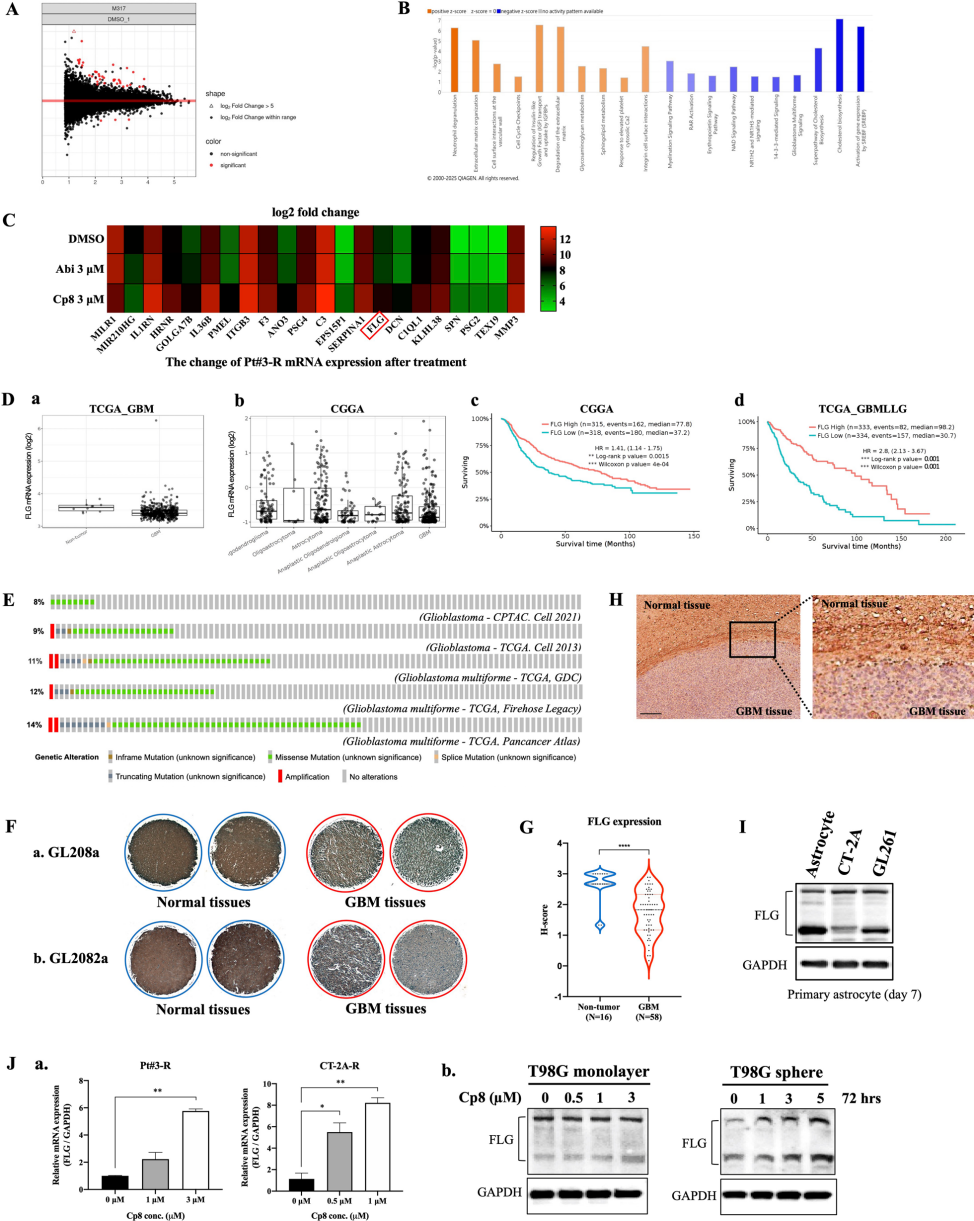
RNA sequencing analysis of cp8-influenced gene expression. **A** Global depiction of fold-change differences on a log scale of cp8-modulated genes represented by a volcano scatter plot. **B** Top biological pathways that are upregulated (orange bars) and downregulated (blue bars) by cp8. **C** Heatmap illustrating the mRNA expression patterns of selected genes significantly regulated by cp8 after different treatment conditions, including DMSO, abi, and cp8. **D** *a* FLG mRNA expression in GBM patients compared to non-tumor tissues, *b* in different brain tumors, *c* the correlation between FLG expression and patient survival outcome from TCGA and CGGA databases. **E** The proportion of mutated FLG in GBM from different datasets. **F** The representative images of FLG expression in normal tissues and patient GBM specimens from tissue arrays GL208a (*a*) and GL2082 (*b*) were examined by IHC staining. G. *H*-score was calculated from IHC tissue array results. **H** IHC staining images of FLG expression in experimental mouse normal and GBM tissues. **I** FLG protein expression in primary mouse normal astrocytes (on day 7) and glioma cells, including CT-2A and GL261, was determined by western blotting. **J** GBM cells, including TMZ-resistant cells and MGMT-positive monolayer and stem-like cells, were treated with cp8 for 72 h, then a. FLG mRNA levels were quantified by qPCR and b. FLG protein expression was analyzed using immunoblotting. Mean ± S.E.M of triplicate experiments. *, P < 0.05; **, P < 0.01; ****, P < 0.0001. Scale bar: 100 μm

To achieve a better understanding underlying cp8-mediated GBM repression, RNA-seq on Pt#3-R was conducted to identify the cp8-regulated genes. Data analysis from the volcano plot indicated that cp8 significantly altered gene expression, with forty genes upregulated and three genes downregulated compared to the control group (Fig. 3A). Specifically, cp8 induced significant changes in various biological pathways, affecting both immune response and cellular metabolism (Fig. 3B). The upregulation of immune-related pathways and downregulation of cholesterol biosynthesis highlight potential therapeutic mechanisms or side effects of the compound. As shown in Fig. 3C, the heatmap showed a noticeable downregulation of FLG expression under the treatment condition with cp8 compared to the control. The significance of survival difference based on gene expression was also verified by The Cancer Genome Atlas (TCGA) and Chinese Glioma Genome Atlas (CGGA) database, in which higher expression of FLG was correlated with better survival outcomes (Fig. 3D, Fig. S4).

Among the genes that exhibited expression changes similar to the FLG gene in the RNA-seq analysis, the consistency between qPCR results and RNA-seq data and the clinical relevance of mRNA expression and patient survival from TCGA and CGGA datasets were further explored (Fig. S5). As a result, FLG was prioritized based on its consistent regulation across datasets, biological relevance to GBM-associated pathways, and its novelty in the context of GBM. Given the limited existing knowledge regarding the functional role of FLG in GBM, we focused subsequent analyses on the FLG gene to provide novel mechanistic insight, while clearly acknowledging the relevance of previously characterized cancer-associated genes.

To gain deeper insights into the role of FLG in GBM as well as the relationship between FLG-mediated GBM and the impact of cp8 on this gene, FLG expression in GBM from different datasets was analyzed, in which the proportion of mutated FLG was enhanced in GBM, ranging from 8 to 14% (Fig. 3E). Findings from IHC staining on GBM patient-derived specimens from tissue arrays demonstrated a remarkable reduction of FLG expression in patient GBM tumors compared to non-tumor samples (Fig. 3F, G). Besides, IHC staining results from experimental mouse GBM tumours also showed that FLG expression was significantly lower than in normal tissue (Fig. 3H), and immunoblotting finding obviously showed a downregulation in FLG expression in CT-2A and GL261, two mouse glioma cell lines, compared to mouse primary astrocytes (Fig. 3I). Validation of gene expression after treatment with cp8 showed consistent findings when FLG mRNA and protein expression were notably upregulated in cp8-treated GBM cells compared to untreated cells, whereas this was not observed when using SAHA, a pan-HDAC inhibitor, to treat GBM cells (Fig. 3J, Fig. S6). This indicates that FLG expression-enhancing activity might be specific to cp8, potentially leading to suppressed GBM cell growth and proliferation.

**Fig. 4:**
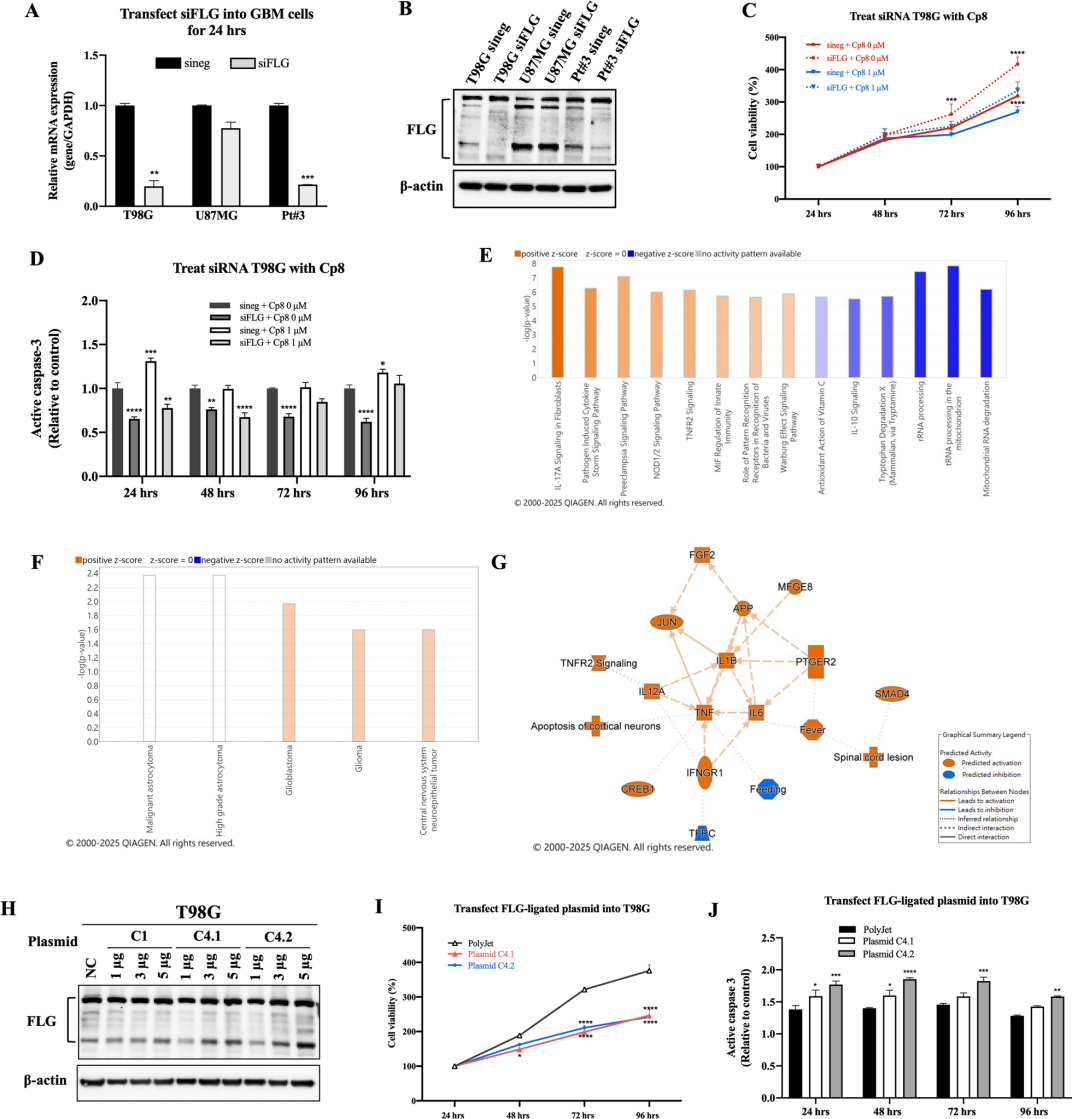
The functional significance of FLG in GBM and the impact of cp8 on FLG gene. **A**, **B** The FLG gene of GBM cells was knocked down using siFLG transfection, and the FLG mRNA and protein expression was validated after being knocked down. **C** Cell viability assay and active caspase-3 determination analysis were performed to evaluate the effects of FLG and cp8 on GBM cells after treatment with cp8, respectively. **E**–**G** RNA-seq of siFLG Pt#3 cells was performed and compared with the control, followed by IPA analysis, as shown by a conconical pathway analysis, a disease and function analysis, and a graphical summary network, respectively. **H** Immunoblotting-confirmed FLG overexpression by the CRISPR-Cas9 method, followed by the plasmid-driven transfection. **I**, **J**. Cell viability assay and active caspase-3 measurement were conducted to examine the significance of FLG upregulation on GBM cell proliferation, respectively

Furthermore, after confirming the knockdown of FLG in GBM cells via siRNA transfection by qPCR and immunoblotting (Fig. 4A, B), to ensure accurate interpretation, we continued to perform downstream analyses primarily in T98G and Pt#3. We demonstrated that siFLG T98G cells without treatment exhibited the highest survival rate, along with the lowest levels of activated caspase 3 after 96 h. In contrast, in the presence of both normal FLG and 1 µM of cp8, the T98G cell count was substantially reduced, as illustrated by the lowest cell viability and the highest level of active caspase 3 (Fig. 4C, D).

Additionally, after knocking down FLG on GBM cells, RNA-seq was further analyzed. IPA pathway analysis reveals significant alterations in inflammatory and innate immune-related signaling, including IL-10 signaling, TLR/NOD pathways, and interferon- and cytokine-associated responses, indicating broad immune and stress response activation (Fig. 4E) [35–38]. In addition, disease and function shows enrichment of cancer-related categories, particularly glioblastoma and glioma, suggesting that loss of FLG gene may influence transcriptional programs linked to glioblastoma- and glioma-associated pathways and cellular dysregulation (Fig. 4F). These results imply that FLG depletion interferes with immune regulation and pathways involved in cancer development or cellular stress. Specifically, the factors shown in the graphical summary network, particularly IL1B, TNF, IL6, FGF2, JUN, PTGER2, and CREB1, form a pro-inflammatory and growth-factor signaling network that enhances GBM plasticity by sustaining transcriptional reprogramming, stem-like states, and adaptive stress responses. These signals drive phenotypic switching, invasion, and therapy resistance, thereby promoting aggressive GBM progression (Fig. 4G). The results revealed the contribution of the FLG gene in GBM development and highlighted cp8 as a promising treatment option for FLG-targeted GBM.

Beside silencing FLG, the FLG overexpression by transfecting FLG-ligated plasmid into GBM cells using CRISPR approach was also performed. FLG overexpression reduced T98G viability, and the levels of active caspase 3 of these groups were also increased compared to the control (Fig. 4H-J). Of importance, FLG showed the ability to boost the susceptibility of GBM cells to TMZ. T98G cells overexpressing FLG had significantly lower viability and higher caspase 3 levels than the control group (Fig. S7) when the cells were treated with TMZ 600 µM. These results show that FLG accelerates the vulnerability of MGMT-positive GBM cells to the cytotoxic effect of TMZ, and enhancing FLG though cp8 offers a promising strategy in suppressing TMZ-resistant GBM.

### Compound 8 possesses suppressive competency in MGMT expression and glioma stem cell development

**Fig. 5:**
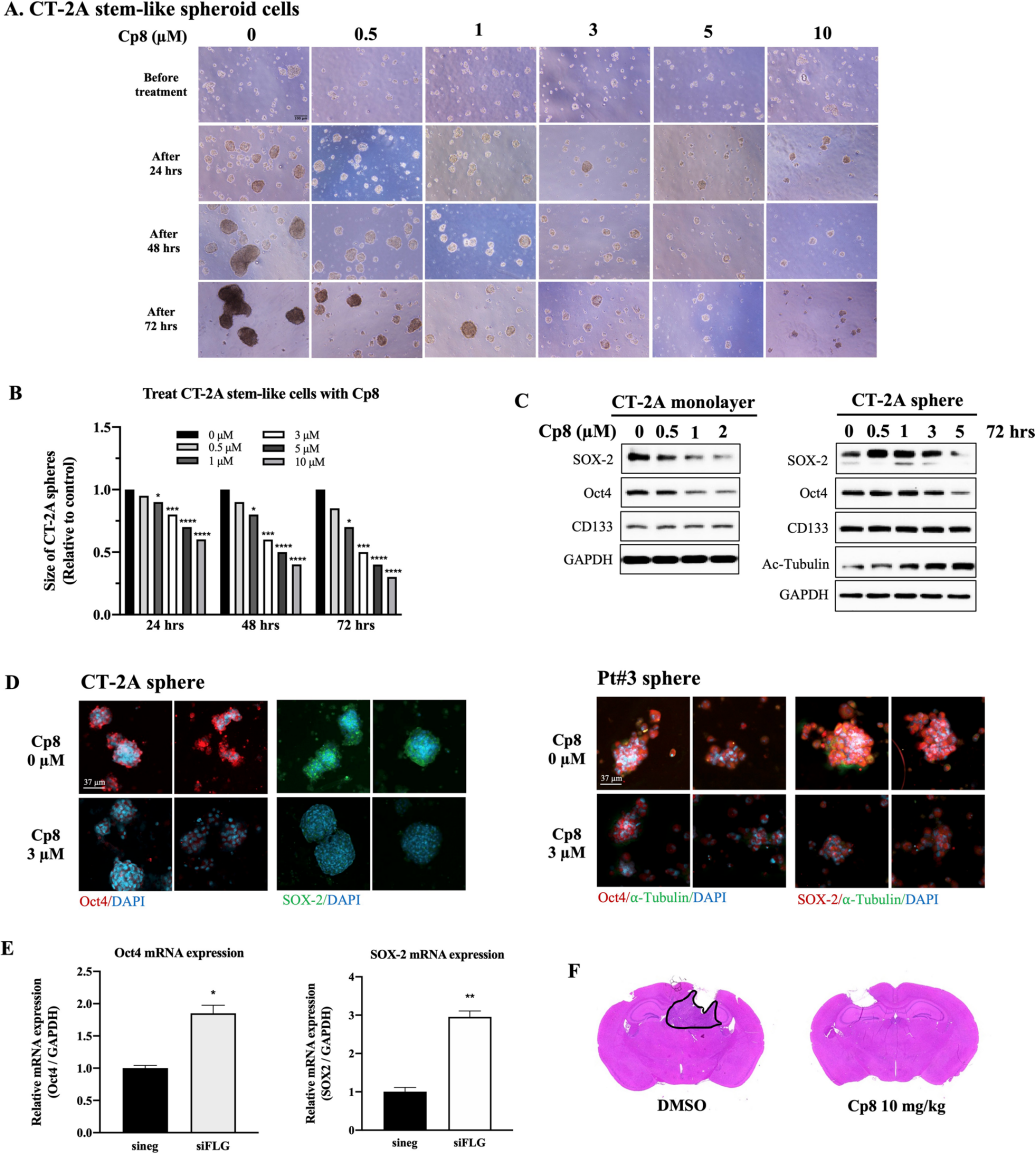
The glioma stem cell suppressive competency of cp8. **A** Stem-like spheroid cells were cultured and then treated with cp8 at multiple doses for 72 h. Scale bar: 100 μm. **B** The size of cp8-treated spheres at different concentrations after 72-h treatment was measured by ImageJ software. **C** GSC marker expression of cp8-treated GBM monolayer and stem-like cells was detected using western blotting. **D** GSC marker expression, including Oct4 and SOX-2 of different cp8-treated GBM stem-like cells, was examined by IF staining. Scale bar: 37 μm. **E** The mRNA expression of Oct4 and SOX-2 after knocking down FLG on GBM cells was measured using qPCR. **F** H&E staining result of Pt#3 sphere-induced tumor mice brains after being injected with Cp8 10 mg/kg compared to the DMSO group. Mean ± S.E.M of triplicate experiments. *, P < 0.05; **, P < 0.01; ****, P < 0.0001

MGMT expression and GSC enrichment are known to significantly contribute to TMZ resistance and GBM recurrence [39], presenting substantial challenges in the development of anti-GBM therapies. In this study, there was a notable reduction in MGMT protein and mRNA levels of MGMT-positive GBM cells, T98G, when cells were treated with cp8 at 3 µM (Fig. S8). Besides, compared to controls, cp8 significantly reduced the size of CT-2A spheres at multiple doses (Fig. 5A, B). Additionally, the expression of SOX-2 and Oct4 proteins was significantly reduced when adherent cells and spheres were treated with cp8 (Fig. 5C). The inhibitory effect of cp8 on HDAC6 was confirmed by the accumulation of acetylated tubulin in CT-2A spheres after treatment (Fig. 5C). IF-stained sphere images showed that the expression of Oct4 and SOX-2 was remarkably weakened after exposure to cp8 (Fig. 5D). These findings demonstrated that cp8 manifested the ability to suppress the aggregation and development of GSCs. Specifically, knocking down FLG resulted in a significant increase in mRNA expression levels of Oct4 and SOX-2 compared to the negative control (Fig. 5E), suggesting that FLG depletion may promote GBM growth associated with GSC development.

To investigate whether MGMT status modulates the inhibitory effect of cp8 in GBM cells, MGMT was overexpressed in Pt#3 cells followed by cell viability and CellROX assays. Cell viability analysis demonstrated that MGMT overexpression attenuated the antiproliferative effect of cp8, with a significant increase in viability observed in Pt#3-MGMT cells compared with control cells, particularly at 72 h of treatment (Fig. S9). In contrast, CellROX analysis showed comparable levels of cp8-induced ROS production in Pt#3 and Pt#3-MGMT cells (Fig. S9). These results indicate that while MGMT overexpression reduces the growth-inhibitory efficacy of cp8, it does not significantly affect cp8-mediated ROS generation.

More importantly, in a xenograft orthotopic model using Pt#3 stem-like cells to induce tumors in NOD-SCID mice, it can be seen that cp8 exhibits the potential to inhibit the tumor growth and reduce the tumor size compare to DMSO group (Fig. 5F). Collectively, these findings indicate that cp8 exhibits significant antitumor activity and has the potential to suppress GBM growth targeting stemness in both in vitro cell models and in vivo tumor settings.

### Compound 8 disrupts reactive oxygen species metabolism

**Fig. 6:**
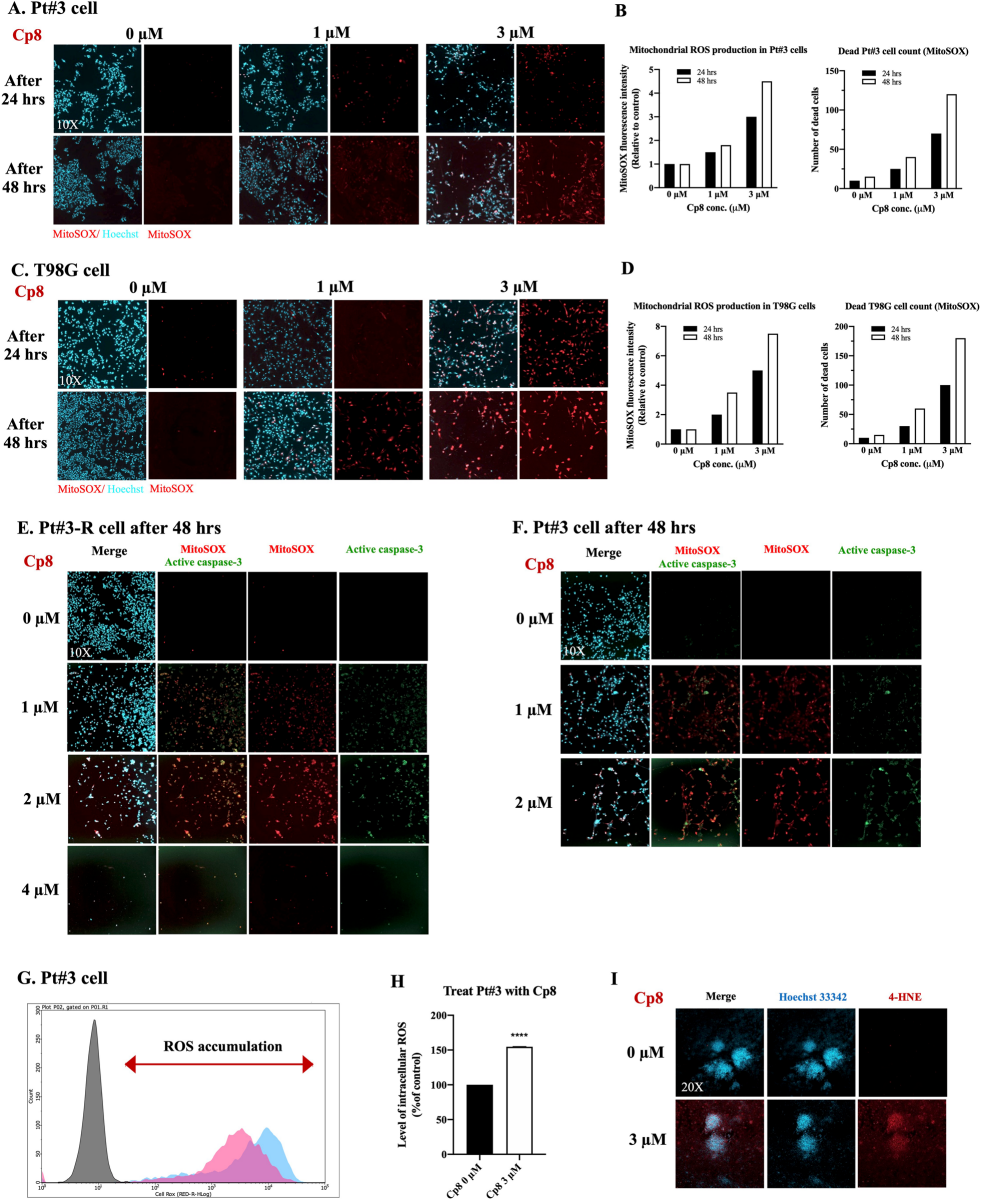
The disruption effect of cp8 on GBM ROS metabolism. **A** and **C** Mitochondrial ROS metabolism of different GBM cells that were treated with multiple doses of cp8 for 72 h was labelled with MitoSOX reagent, then detected with the red signals under the fluorescence microscope. **B** and **D** MitoSOX fluorescence intensity of cp8-treated GBM cells and the number of dead cells were determined by ImageJ software. **E**, **F** Simultaneous visualization of mitochondrial ROS production (MitoSOX red fluorescence) and apoptotic cell populations (active caspase-3 green fluorescence) when Pt#3 and Pt#3-R were treated with cp8 for 48 h. **G** Endogenous ROS production in cp8-treated GBM cells was stained by CellROX kit, followed by flow cytometric analysis. **H** The bar graph of intracellular ROS accumulation quantification in GBM cells that were treated with cp8 compared to the control. **I** Accumulated toxic lipid peroxidation damage of GBM spheres was determined using 4-HNE staining after the spheres were exposed to cp8 at 3 µM. Mean ± S.E.M of triplicate experiments. ****, P < 0.0001

Previously, we showed that ROS clearance is required by GBM to develop TMZ resistance, and ROS accumulation is able to sensitize GBM to TMZ treatment. Herein, compared to controls, cells treated with 3 µM of cp8 for 48 h showed the highest fluorescence intensity of mitochondria-derived ROS and superoxide in Pt#3 and T98G cells (Fig. 6A–D). In addition, MitoSOX assay and co-staining Pt#3 and Pt#3-R cells with active caspase-3, a well-established marker of apoptosis, and Hoechst 33342 for nucleus staining were performed. This approach enables simultaneous visualization of mitochondrial ROS production (MitoSOX red fluorescence) and apoptotic cell populations (active caspase-3 green fluorescence) within the same cells. As shown in Fig. 6E and F, both the mitochondrial ROS and active caspase-3 signals of the cells increased markedly in a dose-dependent manner following 48-h treatment with cp8. Specifically, it can be observed that Pt#3-R cells also exhibit better resistance to cp8 than Pt#3 cells, with a few alive Pt#3-R cells being observed after cp8 treatment at 48 h, while surviving Pt#3 cells are almost completely inhibited after exposure to cp8 at 4 µM. These results confirm that the observed MitoSOX signal corresponds to mitochondria-associated ROS and is closely associated with apoptotic cell death caused by cp8 rather than nonspecific staining or unrelated oxidative events. Treatment of Pt#3 cells with cp8 3 µM resulted in a pronounced increase in intracellular ROS levels as measured by the CellROX assay. Flow cytometric analysis demonstrated a clear rightward shift in CellROX fluorescence intensity in cp8-treated cells compared with the control, indicating ROS accumulation. Quantification confirmed a significant elevation of intracellular ROS in cp8-treated cells relative to control (Fig. 6G, H). These results demonstrate that cp8 induces potent oxidative stress in patient-derived GBM cells.

Moreover, 4-HNE was also used to evaluate the effect of cp8 on the oxidative damage process of GBM cells. As shown in Fig. 6I, there is a higher expression of 4-HNE signals in the treatment group with cp8 than in the control, demonstrating that cp8 induces accumulated toxic lipid peroxidation damage, and this compound affects downstream oxidative stress in GBM mitochondria.

These findings determined the effect of cp8 on boosting ROS production and causing ROS leakage from GBM mitochondria in a dose- and time-dependent manner, leading to ROS metabolism dysfunction in GBM cells.

### Compound 8 suppresses GBM tumor growth and prolongs the survival period while also demonstrating its safety in vivo

**Fig. 7:**
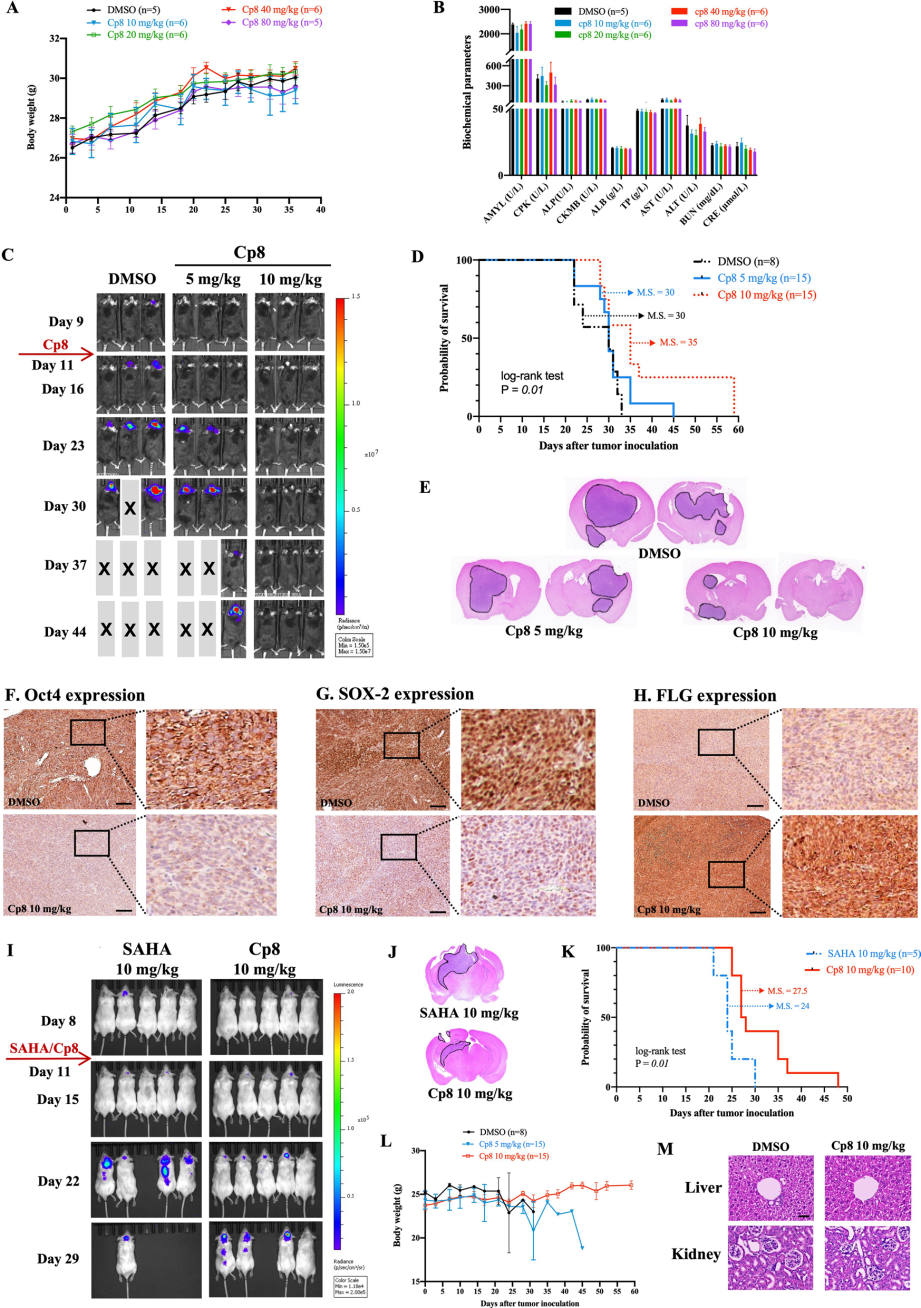
The tolerability of cp8, and the tumor suppressive efficacy of cp8 in mouse experiments. **A** Mice were administered DMSO or cp8 with doses at 10–80 mg/kg, i.p., three times per week, and the body weight was noted during the experiment. **B** Mice were sacrificed, and blood was taken for biochemical analysis. **C** Representative images of experimental mice when they were i.p. administered with cp8 at different doses, and the tumor growth was monitored by the IVIS imaging system weekly. **D** After tumor cells were implanted, the mouse survival days were recorded and analyzed using a log-rank test. **E** Mouse brain tissues were subjected to H&E staining after being sacrificed, followed by the tumor size examination. **F**, **G**, **H** IHC staining results of Oct4, SOX-2, and FLG expression in experimental mouse GBM tumors after they were sacrificed, respectively. **I** Representative images of SAHA-treated and cp8-treated mice at 10 mg/kg, and the tumor growth was detected using IVIS imaging. **J** Mouse brain tumors were excised and subjected to H&E staining to examine the tumor size. **K** Log-rank test was applied to the recorded and analyzed survival data of mice. **L** Mouse body weight was taken note during the experiment. **M** Representative images illustrating H&E staining performed on liver and kidney tissues collected from mice in experimental groups. Scale bar: 100 μm

To evaluate the tolerability and potential toxicity of cp8, C57BL/6 mice were treated with escalating doses of cp8 ranging from 10 to 80 mg/kg (n = 5–6 per group). Among all dose levels, mice maintained stable or gradually increasing body weight throughout the study, with no evidence of dose-dependent weight loss, indicating good general tolerability (Fig. 7A). Blood biochemical analysis demonstrated that key chemical parameters, including markers of hepatic function (ALT, AST, and ALP), renal function (BUN and creatinine), and metabolic status, remained comparable among the treatment groups and the control group (Fig. 7B). These findings suggest that cp8 was well tolerated at multiple doses up to 80 mg/kg under the conditions tested, with no clear dose-dependent abnormalities of systemic toxicity.

Results from the CT-2A-induced brain tumor allograft model showed that cp8 at 10 mg/kg injected i.p. twice a week significantly inhibited GBM tumor growth and prolonged the mouse survival time up to 59 days compared to the DMSO group (34 days) (Fig. 7C, D and Table S5). Notably, i.p. administration with 10 mg/kg of cp8 markedly reduced the tumor size in experimental mice compared to the control group (Fig. 7E). Furthermore, IHC staining was conducted on the experimental mice brains to investigate how cp8 affected GSC development and FLG level in vivo. Consistently, as shown in Fig. 7F and G, it is evident that the treatment group using cp8 at 10 mg/kg exhibited a significant decrease in Oct4 and SOX-2 expression in brain tumor tissues compared to the DMSO group. Besides, there was a noticeable increase in FLG expression in the tumor of the mice brains treated with cp8, as opposed to the DMSO group (Fig. 7H). These results greatly supported our findings on GSC enrichment-reducing and FLG-augmenting effects of cp8.

To compare the potency of cp8 with the control substance SAHA, an orthotopic xenograft U87MG-luc-induced brain tumor model was conducted. IVIS imaging results demonstrated that cp8 outperformed SAHA at the same dose of 10 mg/kg in reducing tumor size (Fig. 7I, J). Furthermore, i.p. administration of cp8 also extended the survival period of experimental mice compared with SAHA (49 days *vs.* 30 days) (Fig. 7K and Table S5). Importantly, mice given cp8 at 10 µM displayed normal eating, movement, and behavior (data not shown), and maintained their body weight throughout the experiment (Fig. 7L). In addition, H&E staining of mouse liver and kidney sections revealed no evidence of liver and kidney injury, necrosis, and inflammation in the treatment group compared to the DMSO group, indicating its low toxicity (Fig. 7M).

The findings from animal experiments highlighted the potential of cp8 to increase the survival rate and decrease the tumor size while also proving its safety in vivo*.*

**Fig. 8:**
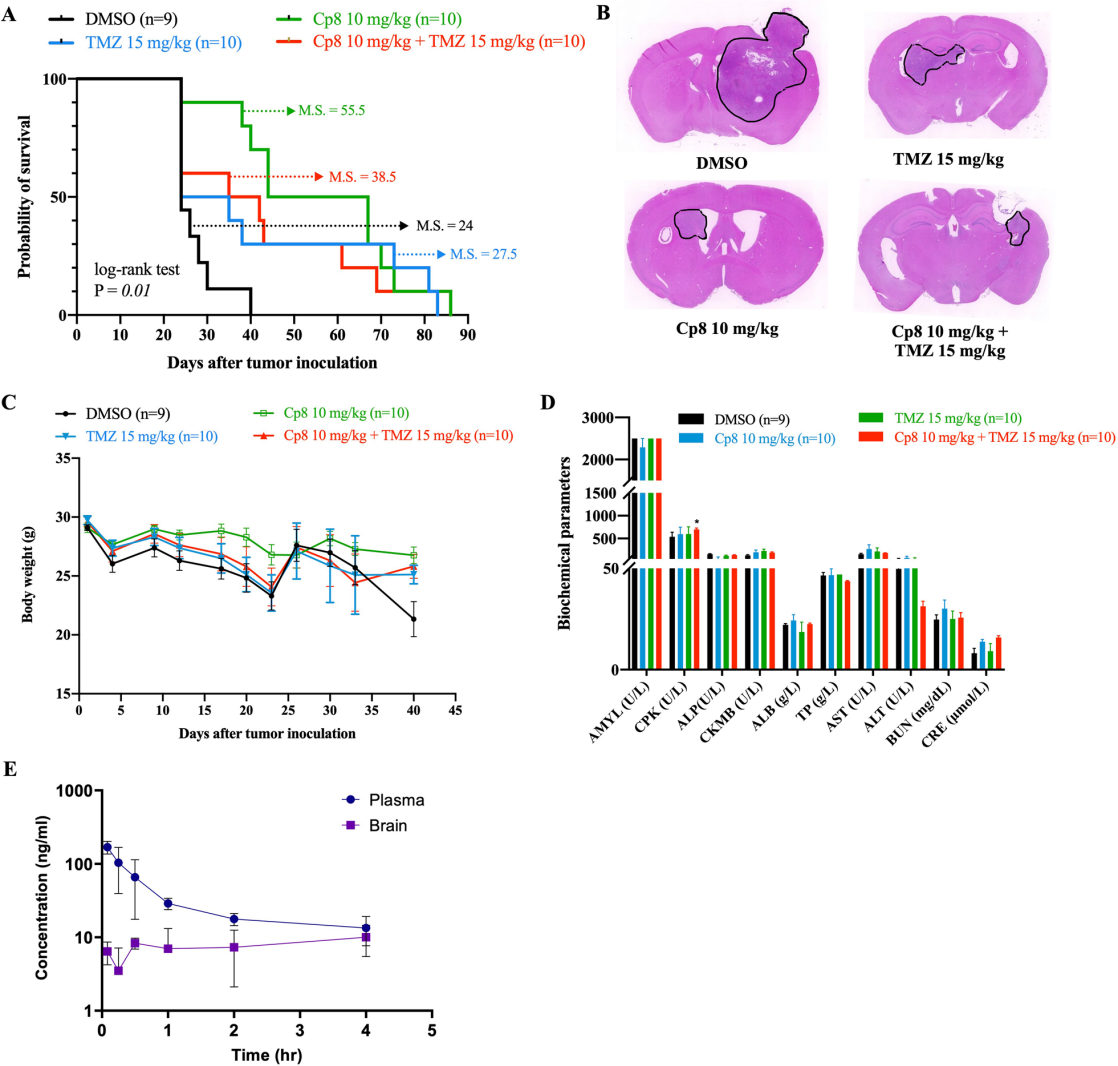
The cp8-TMZ synergy, and the concentration–time profile of cp8 in brain and plasma. **A** The mice survival period was analyzed using the log-rank test. **B** Mouse brain tumors were excised and subjected to H&E staining to examine the tumor size of the experimental groups. **C** Mice body weight was recorded throughout the experiment. **D** Mice were sacrificed, and the mice blood was collected for biochemical testing. **E** Plasma and brain concentration–time profile of cp8 following a single i.v. administration of 2 mg/kg in rats

An orthotopic xenograft model using Pt#3-R cells on NOD-SCID mice was used to investigate whether there is a synergistic effect of cp8 and TMZ. Consistent with previous findings, cp8 significantly prolonged the mean survival day (55.5 days vs. 24 days compared to DMSO) and reduced tumor growth as a single agent (Fig. 8A, B). However, no statistically significant synergistic benefit, including changes in mice body weight, was observed when combined with TMZ (Fig. 8C). The strong efficacy of cp8 alone, especially on TMZ-resistant cells, may have limited the dynamic range to detect additional benefit from the combination treatment. In addition, the combination dosing and scheduling were selected based on tolerability rather than optimization for synergy, and alternative regimens may be required to evaluate combinatorial effects more fully. Moreover, biochemical analysis showed no significant changes in most tested parameters, with only a slight increase in CPK in the combination group, suggesting that the regimen was generally well tolerated with minimal additional systemic toxicity (Fig. 8D).

Not only does cp8 demonstrate the ability to inhibit GBM by decreasing the tumor size caused by monolayer glioma cells, but it also shows the ability to inhibit tumors caused by Pt#3 sphere-induced tumors (Fig. 5F). TUNEL staining results of experimental mice tumor brains showed that there was an obvious increased TUNEL-positive cells in the treatment group, indicating enhanced apoptosis in vivo (Fig. S10). In addition, 4-HNE staining in Pt#3 spheres in vitro demonstrated increased lipid peroxidation following cp8 treatment, supporting a mechanism involving oxidative stress (Fig. 6I). These complementary in vivo and in vitro data indicate that cp8 effectively induces oxidative stress-associated apoptosis in glioma stem cells and suppresses tumor growth in an orthotopic model.

To analyse the pharmacokinetic profile of cp8 on brain, an experiment study was carried out using normal Sprague–Dawley rats. Following a single i.v. administration of cp8 at 2 mg/kg, the concentration–time profiles in plasma and brain tissue were characterized (Fig. 8E and Table S6). In plasma, cp8 exhibited rapid elimination with an initial concentration C_0_ of 0.23 ± 0.06 μg/mL and a terminal half-life *T*_1/2_ of 0.48 ± 0.18 h. Cp8 was detected in brain tissue shortly after dosing, yielding a brain exposure AUC_0-inf_ of 0.08 ± 0.05 h × g/mL. The calculated brain-to-plasma ratio was approximately 21.4%, demonstrating moderate blood–brain barrier permeability. Notably, brain and plasma concentrations converged toward the 4-h time point, both reaching approximately 10 ng/mL. The pharmacokinetic analysis confirms that cp8 successfully penetrates the blood–brain barrier. The comparable half-lives observed in both compartments (approximately 0.5 h) suggest a rapid equilibrium between the systemic circulation and the brain parenchyma. The high volume of distribution (*V*_d_ = 30.10 L/kg in plasma) indicates extensive tissue partitioning beyond the blood compartment. Interestingly, while the initial plasma-to-brain gradient was steep, the convergence of concentrations at later time points suggests a slower clearance rate from the brain compartment relative to the plasma. These findings indicate that cp8 possesses the necessary profile for central nervous sytem-targeted candidates; however, its high systemic clearance (*Cl* = 12.60 L/hr/kg) and short half-life may necessitate further lead optimization or specialized drug delivery strategies to prolong the therapeutic window within the brain.

## Discussion

Our drug discovery team has been employing a multifaceted approach to design and synthesize novel anti-GBM chemical architectures by structurally enabling HDAC inhibitory pharmacophore templates, which consist of three key components: a surface recognition part (SRP), a linker, and a zinc-binding group (ZBG) [40]. Previous studies have demonstrated that abiraterone has emerged as a structurally and functionally compelling scaffold, indicating its potent anti-GBM activity in both in vitro and in vivo models [27, 41, 42]. Additionally, HDAC inhibitors are attracting significant attention as potential GBM therapeutics due to their epigenetic gene expression regulation and disruption of oncogenic signalling pathways crucial for tumor growth and survival [43–46]. In this study, abiraterone was strategically utilized as the SRP unit, capitalizing on its established anti-GBM efficacy, and a series of abiraterone-based HDAC inhibitors were meticulously designed and synthesized, incorporating structurally diverse linker moieties such as sulfonyl, benzyl acrylamide, benzyl, and benzamide linkers paired with a hydroxamic acid functional group as the ZBG. These linker motifs were selected based on their prevalence and demonstrated efficacy within the structural frameworks of several FDA-approved HDAC inhibitors [47, 48]. Among the derivatives developed, cp8 exhibited a significant inhibitory effect against HDAC isoforms (Table S7). This integration resulted in stronger GBM growth inhibition compared to abiraterone, with an IC50 of 2.77 μM *vs.* 20.87 μM (Table 1), respectively. These findings demonstrate that this consolidation enhanced the anti-GBM potential, with cp8 emerging as a promising candidate for further preclinical development. Its superior efficacy in inhibiting GBM progression and inducing apoptosis, along with its HDAC-inhibitory activity, suggests its potential as a dual-function therapy to overcome GBM resistance to conventional treatments.

In GBM development, ROS has a double-edged effect on tumor progression and therapeutic resistance. While excessive ROS can induce cell death and tumor regression, GBM cells can adapt by enhancing antioxidant defences and suppressing mitochondrial ROS through mechanisms like prohibitin-mediated regulation [49, 50]. This adaptation enables therapy resistance and tumor malignancy, particularly in GSCs, which enhance ROS scavenging to evade apoptosis and sustain growth [8, 49]. ROS modulation is a key factor in TMZ resistance, further complicating treatment [8]. To optimize GBM therapy, a strategic approach is needed, either by boosting ROS-induced cytotoxicity or blocking adaptive antioxidant responses to overcome resistance. Furthermore, the combination of blocking GSC enrichment and reducing MGMT expression further augments the potential to suppress GBM through three important factors contributing to TMZ resistance. In addition, elevated ROS can promote lipid peroxidation, DNA damage, and activation of apoptotic signaling pathways, consistent with the increased 4-HNE staining and enhanced TUNEL positivity. In this study, our novel cp8 was shown to significantly increase mitochondrial and intracellular ROS levels, ultimately inducing GBM cell death and tumor repression. Importantly, in vivo data clearly demonstrated the efficacy of cp8, underscoring its potential therapeutic value.

Although many genes controlled by cp8 are linked to cancer, particularly brain tumors, the precise impact and underlying mechanisms of FLG in the context of GBM remain unclear. Consequently, our research would center on the function of this gene in the progression and development of GBM. FLG is a critical element in the development and maintenance of the skin barrier [11]. Several studies have shown that loss-of-function FLG gene mutations compromise skin barrier integrity, thereby increasing the risk of UV penetration. Lower levels of FLG degradation products and UV chromophores in the stratum corneum reduce protection against chronic UV exposure, potentially increasing skin cancer risk [14]. It is recognized as one of the common risk factors for atopic dermatitis and skin cancer [14, 51, 52]. Moreover, targeting FRA1:c-JUN:HDAC1 complex to inhibit HDAC1 activity results in elevated FLG levels, indicating a potential therapeutic approach for inflammatory skin diseases like atopic dermatitis and psoriasis [53]. Our findings revealed lower FLG expression and higher FLG mutation rates in GBM from TCGA and CGGA data correlated with worse survival. The mutated FLG resulted in a loss-of-function FLG, meaning FLG lost its main function. This suggests that GBM may also feature inactivating FLG mutations, resulting in a decrease in FLG expression. Therefore, we aimed to investigate the effects of FLG by manipulating FLG expression levels to uncover the functional significance of FLG in GBM progression. Silencing of FLG in GBM cells led to activation of inflammatory and growth-associated genes, which are key regulators of tumor plasticity. This transcriptional shift is consistent with enhanced stem-like properties, adaptive signaling, and phenotypic flexibility, suggesting that FLG typically limits pathways that drive aggressive GBM progression [35–38]. Besides, while FLG knockdown significantly reduced FLG expression in GBM cells, leading to elevated cell viability and decreased active caspase 3, we encountered challenges in achieving FLG overexpression. The FLG gene encodes the FLG protein, which is initially produced as a large precursor protein called profilaggrin. Genetic variation affects FLG protein length; however, profilaggrin generally comprises about 4,061 amino acids [54, 55]. Due to the large size of the FLG gene, FLG overexpression proved challenging for conventional cloning methods, necessitating innovative gene editing techniques to optimize elevated FLG activity, such as CRISPR-Cas9. To achieve FLG overexpression, various FLG promoter sequences (Table S1) were designed and inserted into plasmid coded #99,698 (https://www.addgene.org/99698/) by the CRISPR-Cas9 approach. The modified plasmids were cloned and introduced into GBM cells to drive FLG overexpression. The plasmid effectively drove FLG overexpression in GBM cells, leading to increased FLG levels compared to the control group. The significant reduction in GBM cell viability and increase in caspase 3 levels clearly showed how higher FLG expression affects GBM cell growth and proliferation. Notably, the findings also demonstrated that FLG significantly enhanced the vulnerability of MGMT-positive GBM cells to the cytotoxic effect of TMZ, a critical observation for potential therapeutic advancements. More importantly, silencing HDAC1, HDAC2, or HDAC6 in GBM cells led to concomitant higher FLG expression and lower SOX-2 expression, suggesting a correlation among HDACs, FLG, and GSC markers (Fig. S11). Cp8, functioning as an HDAC inhibitor, simultaneously boosts FLG and diminishes GSC enrichment, strongly demonstrating a synergistic inhibitory effect on GBM tumorigenesis. In GBM, FLG appears to play a pivotal yet mysterious role in tumor development. Thus, our findings shed light on the role of FLG in GBM pathogenesis and provide insights into the potential therapeutic implications of modulating FLG expression in GBM treatment strategies. Enhancing FLG expression through cp8 offers a promising strategy in suppressing TMZ-resistant glioma, presenting a potential candidate for future FLG-targeted anti-GBM intervention.

Besides the apparent impact of cp8 on GBM suppression, it is indisputable that our study still has unaddressed limitations. Although our research targeted mutated and functional FLG, we lacked antibodies to distinguish them, limiting the analysis to comparing general FLG levels. This suggests the need for a special antibody to detect two kinds of proteins to further elucidate the role of FLG in GBM.

## Conclusion

In summary, our study demonstrated the new abiraterone-based cp8 inhibits GBM growth via multiple mechanisms involving GSC/MGMT suppression, ROS induction, and FLG enhancement. The discovery of FLG, a newly key factor in GBM development, further sheds light on GBM pathogenesis and lays the groundwork for future anti-GBM drug discovery and therapy.

## Supplementary Information

Additional file 1.

Additional file 2.

## Data Availability

The datasets generated and/or analysed during the current study are available from the corresponding author on reasonable request. Due to data use agreements, the data are not publicly available. Where appropriate, de-identified data can be shared upon approval by the relevant ethics committee and completion of a data-sharing agreement.

## Abbreviations

TMZ: Temozolomide
GBM: Glioblastoma
GSC: Glioma stem cell
HDAC: Histone deacetylase
MGMT: O^6^-methylguanine-DNA methyltransferase
FLG: Filaggrin
ROS: Reactive oxygen species
ROI: Region of interest
DMEM: Dulbecco’s Modification of Eagle’s Medium
siRNA: Small interfering RNA
CCK8: Cell counting kit-8
qPCR: Polymerase chain reaction
PBS: Phosphate-buffered saline
RT: Room temperature
IF: Immunofluorescence staining
IHC: Immunohistochemical staining
i.p.: Intraperitoneal/ intraperitoneally
DMSO: Dimethyl sulfoxide
H&E: Hematoxylin and eosin
SRP: Surface recognition part
cp1: Compound 1
cp2: Compound 2
cp3: Compound 3
cp4: Compound 4
cp5: Compound 5
cp6: Compound 6
cp7: Compound 7
cp8: Compound 8
cp9: Compound 9
cp10: Compound 10
IC50: Half-maximal inhibitory concentration
TCGA: The Cancer Genome Atlas
CGGA: Chinese Glioma Genome Atlas
ZBG: Zinc-binding group
