# Supplementary material for: Dual suppression of stemness and redox adaptation in glioblastoma through filaggrin upregulation by an abiraterone-based HDAC inhibitor
Source: J Biomed Sci. 2026 Apr 6;33:38. doi: 10.1186/s12929-026-01241-2 (PMC13051497; doi:10.1186/s12929-026-01241-2)

^1^H NMR of Compound (cp1)


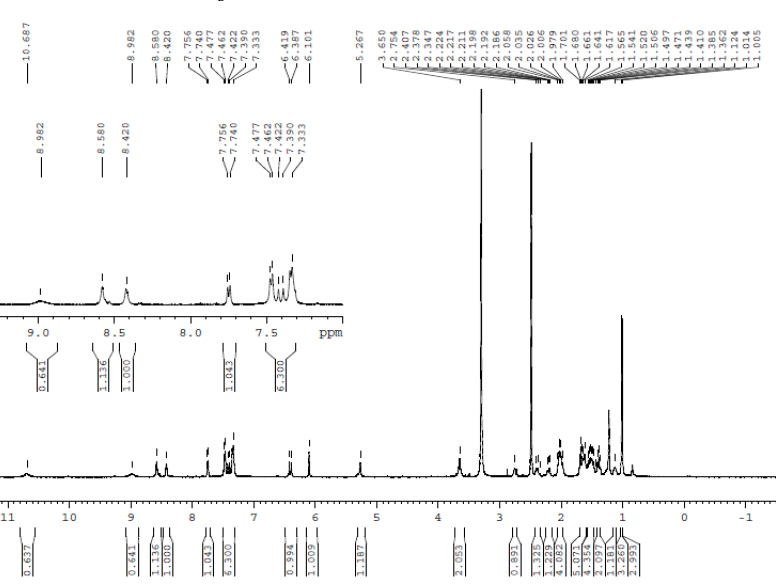


^1^H NMR of Compound (cp2)


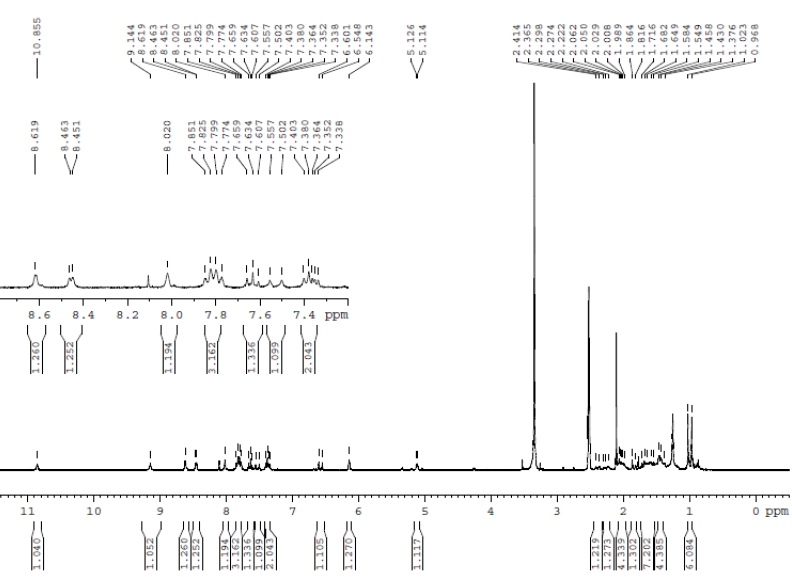


^1^H NMR of Compound (cp3)


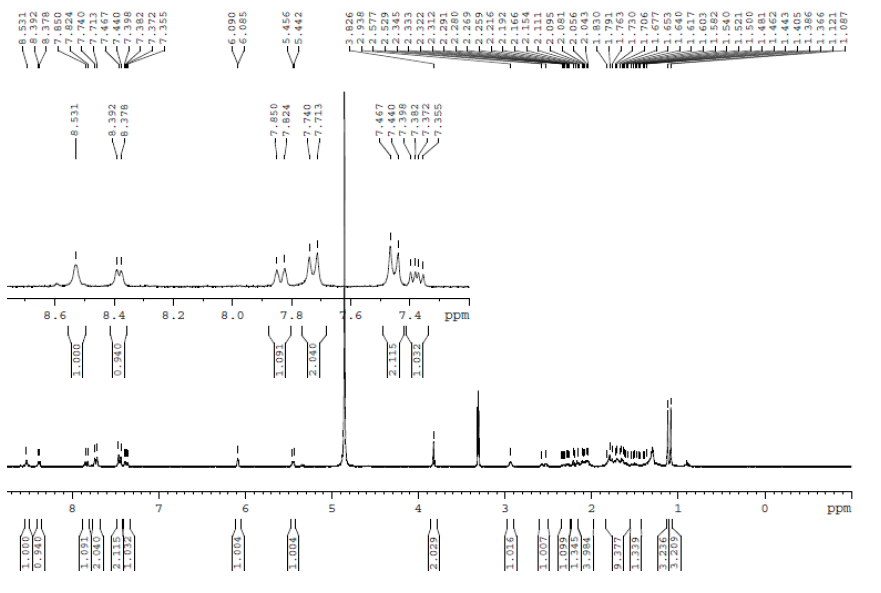


^1^H NMR of Compound (cp4)


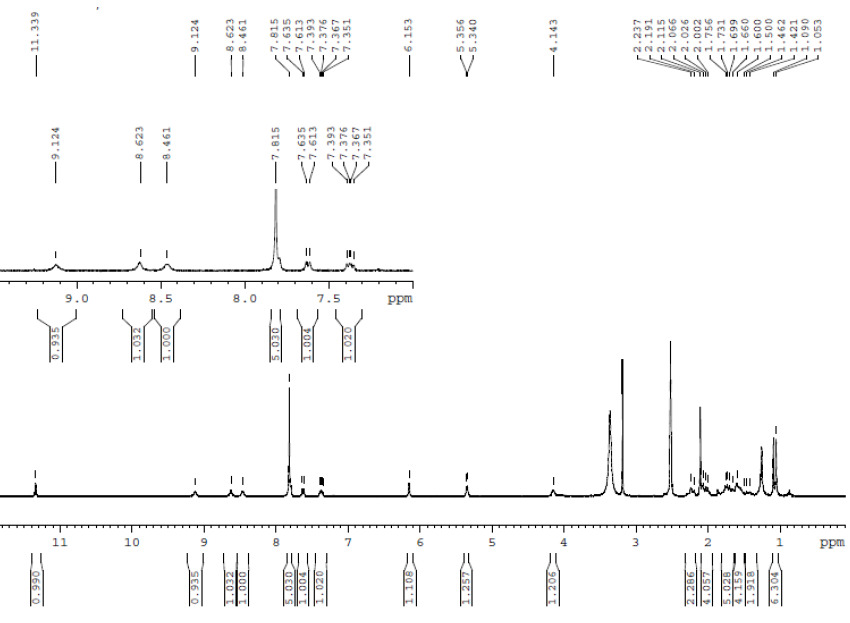


^1^H NMR of Compound (cp5)


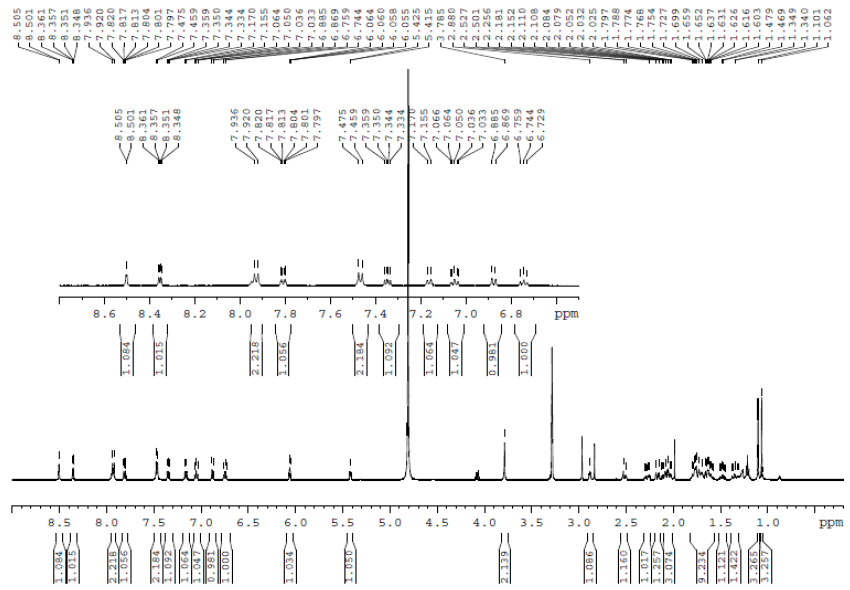


^1^H NMR of Compound (cp6)


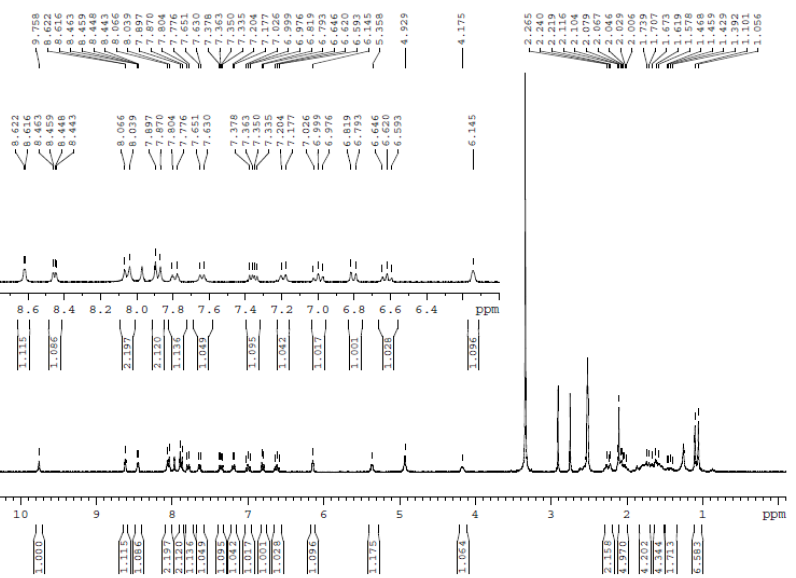


^1^H NMR of Compound (cp7)


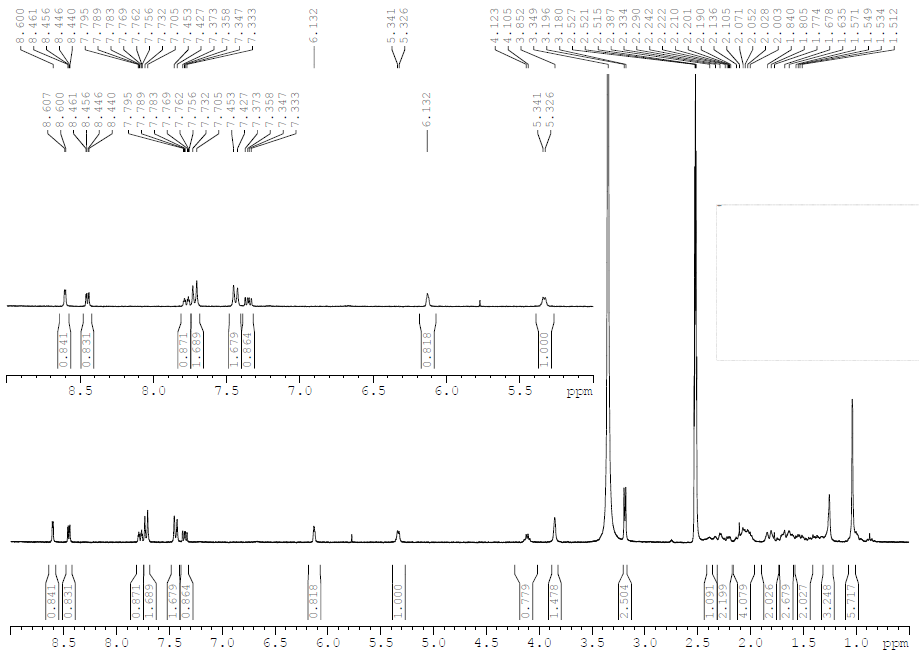


^1^H NMR of Compound (cp8)


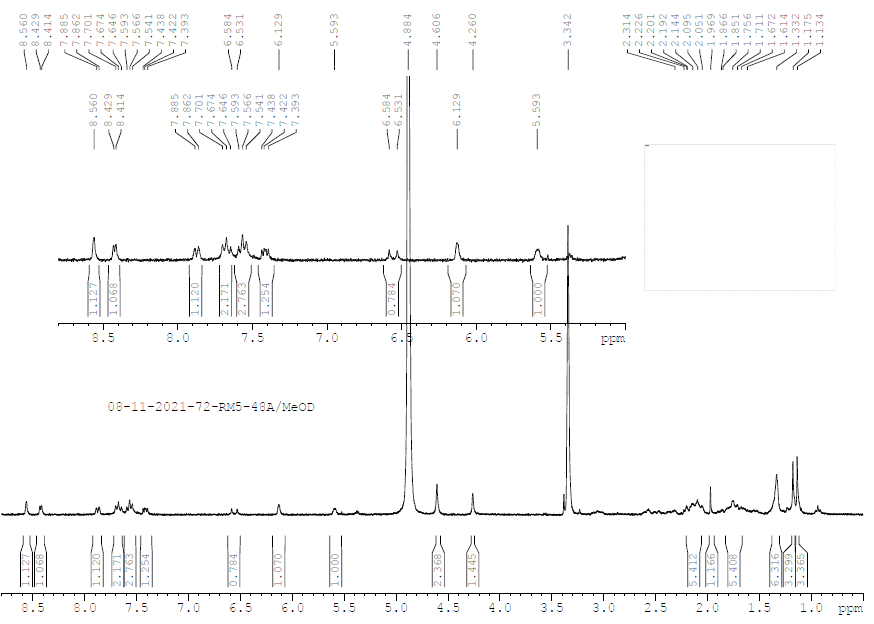


^1^H NMR of Compound (cp9)


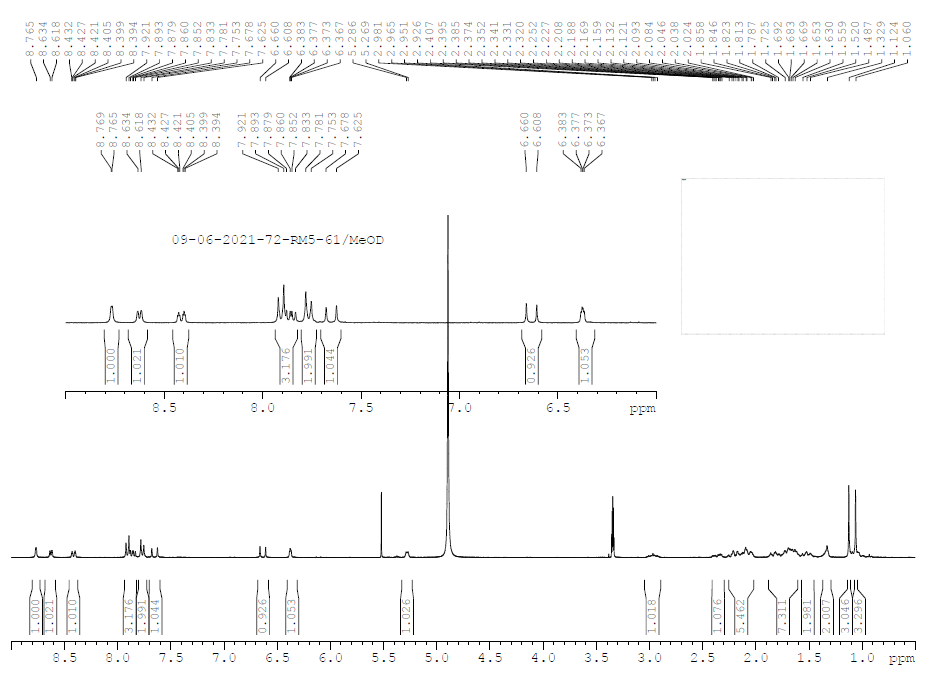


^1^H NMR of Compound (cp10)


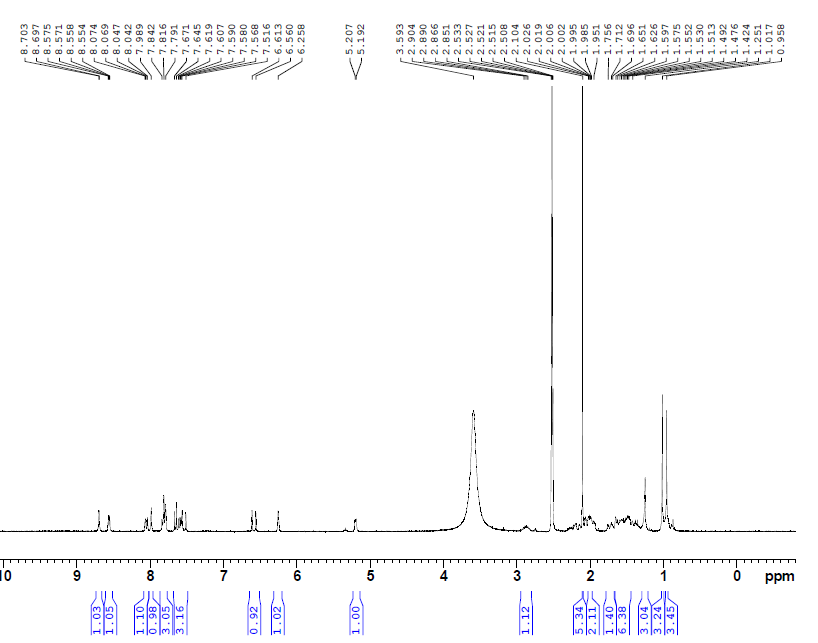


^13^C NMR of Compound (cp1)


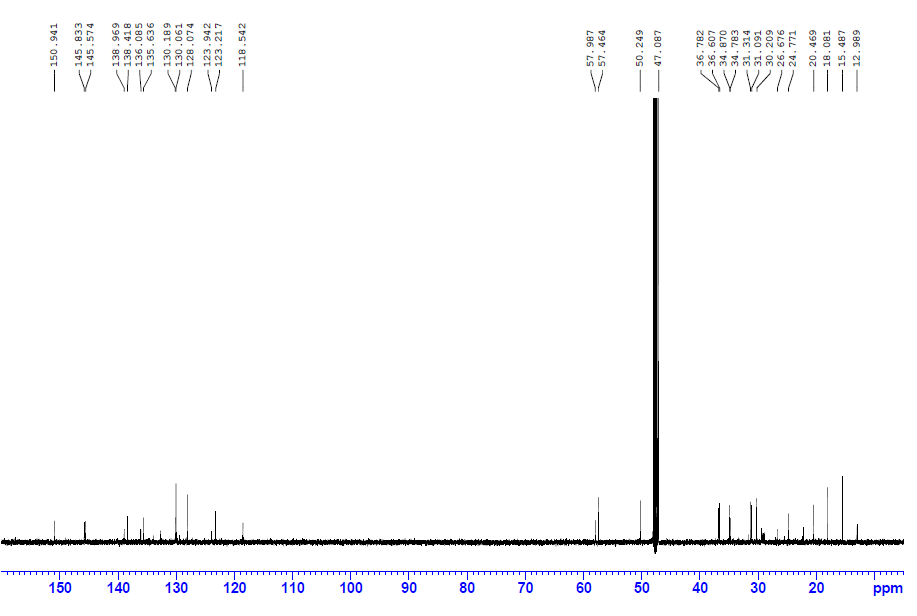


^13^C NMR of Compound (cp2)


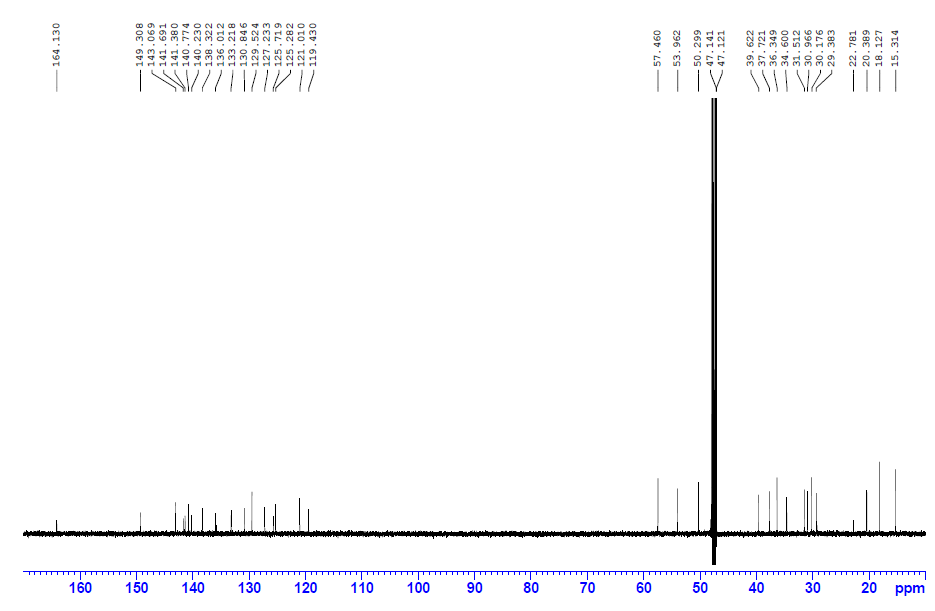


^13^C NMR of Compound (cp3)


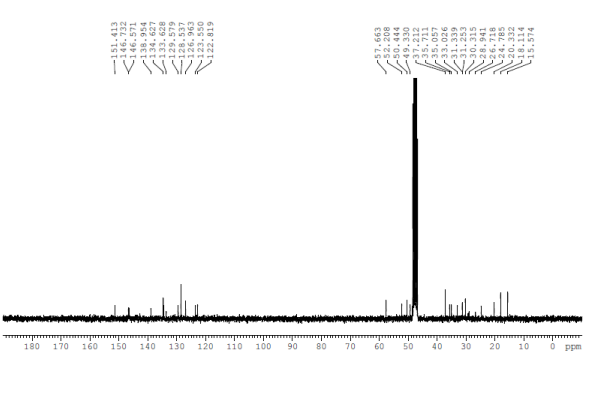


^13^C NMR of Compound (cp4)


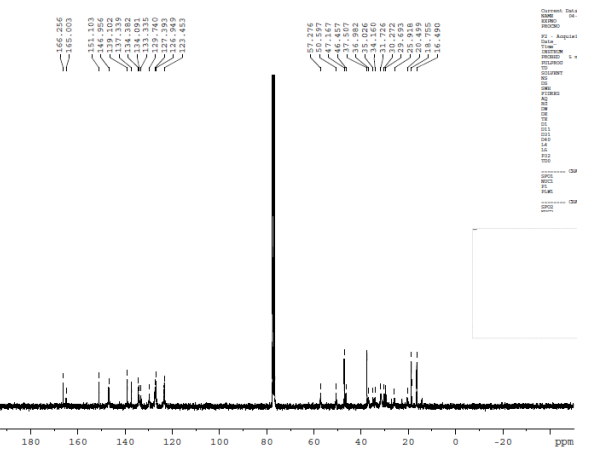


^13^C NMR of Compound (cp5)


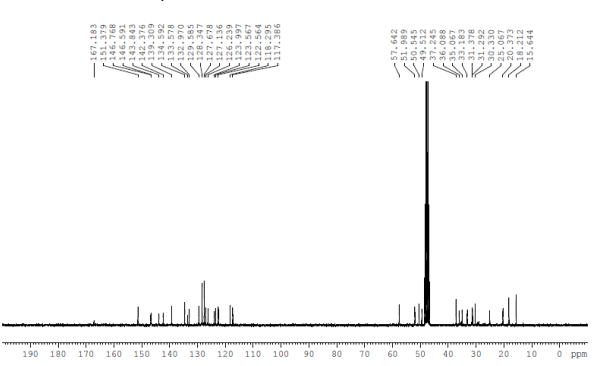


^13^C NMR of Compound (cp6)


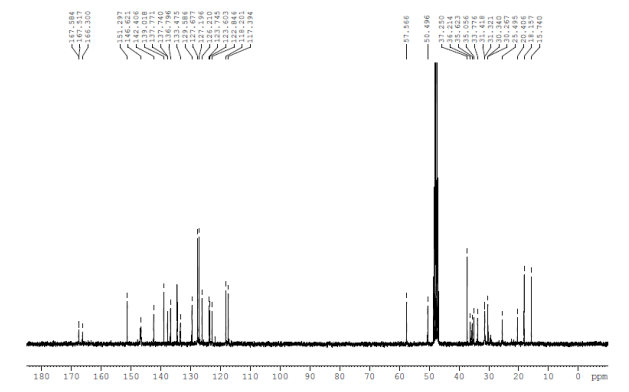


^13^C NMR of Compound (cp7)


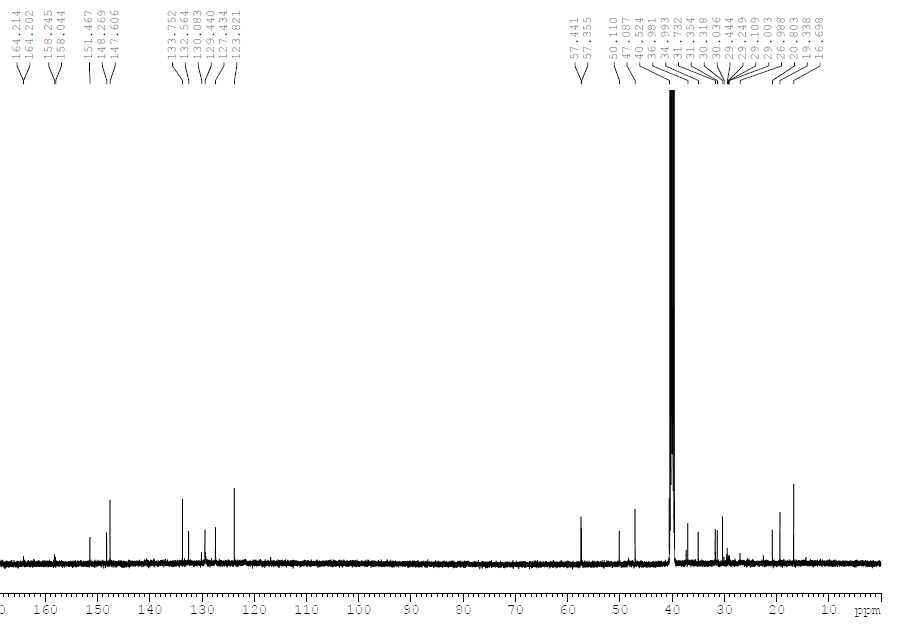


^13^C NMR of Compound (cp8)


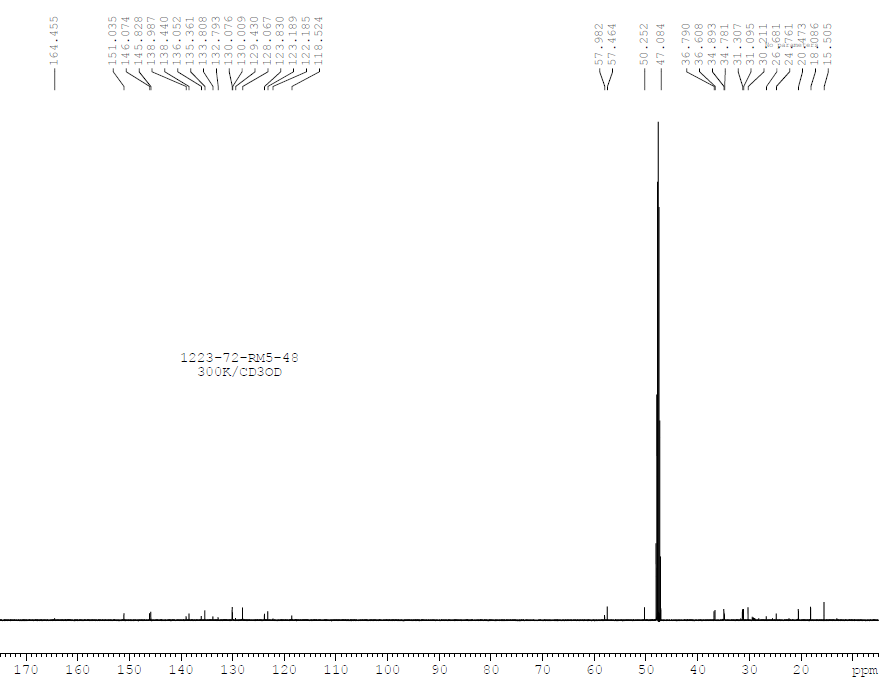


^13^C NMR of Compound (cp9)


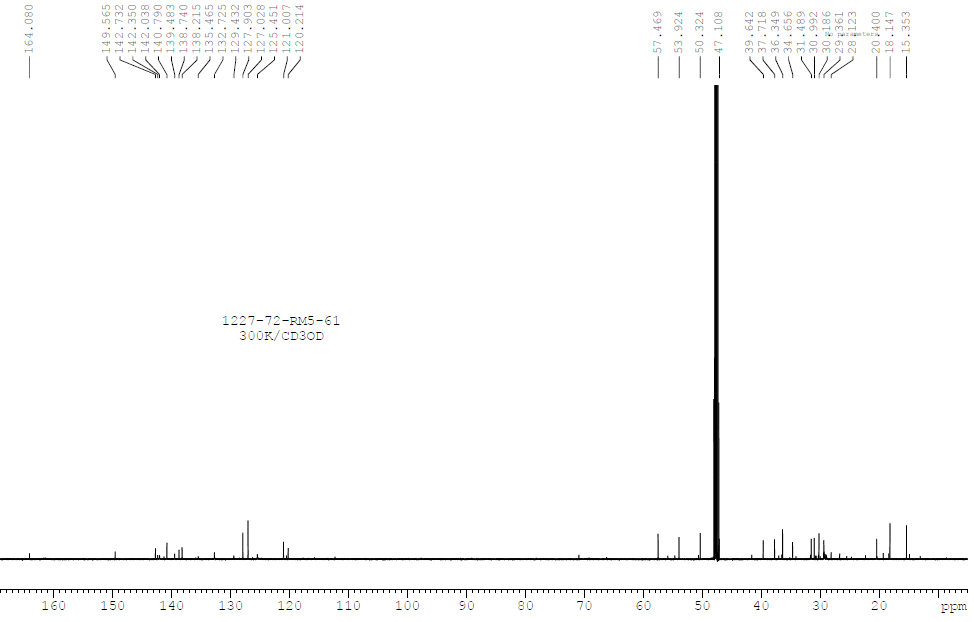


^13^C NMR of Compound (cp10)


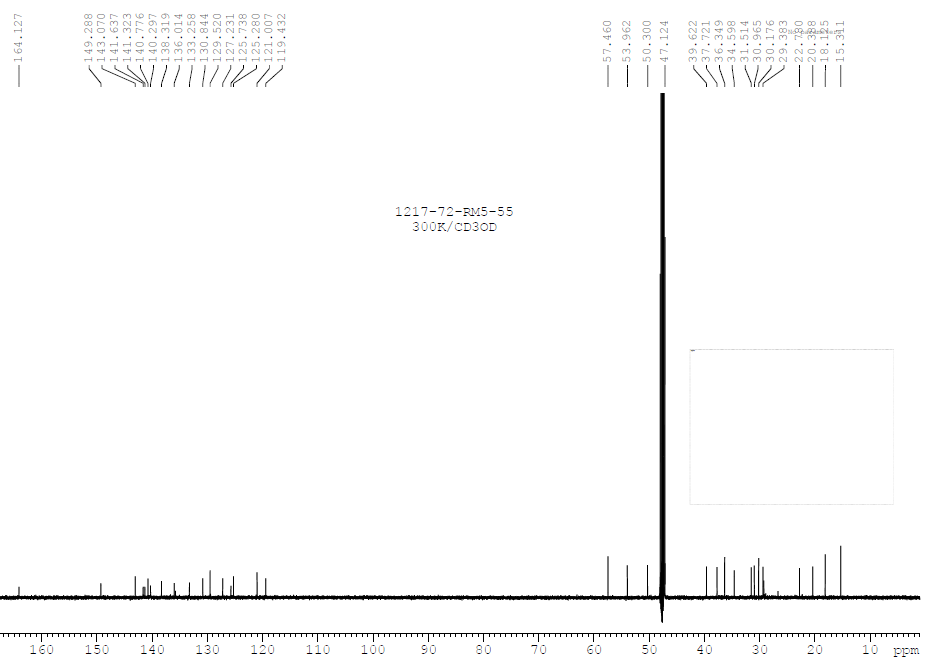


HRMS Data of Compound (cp1)


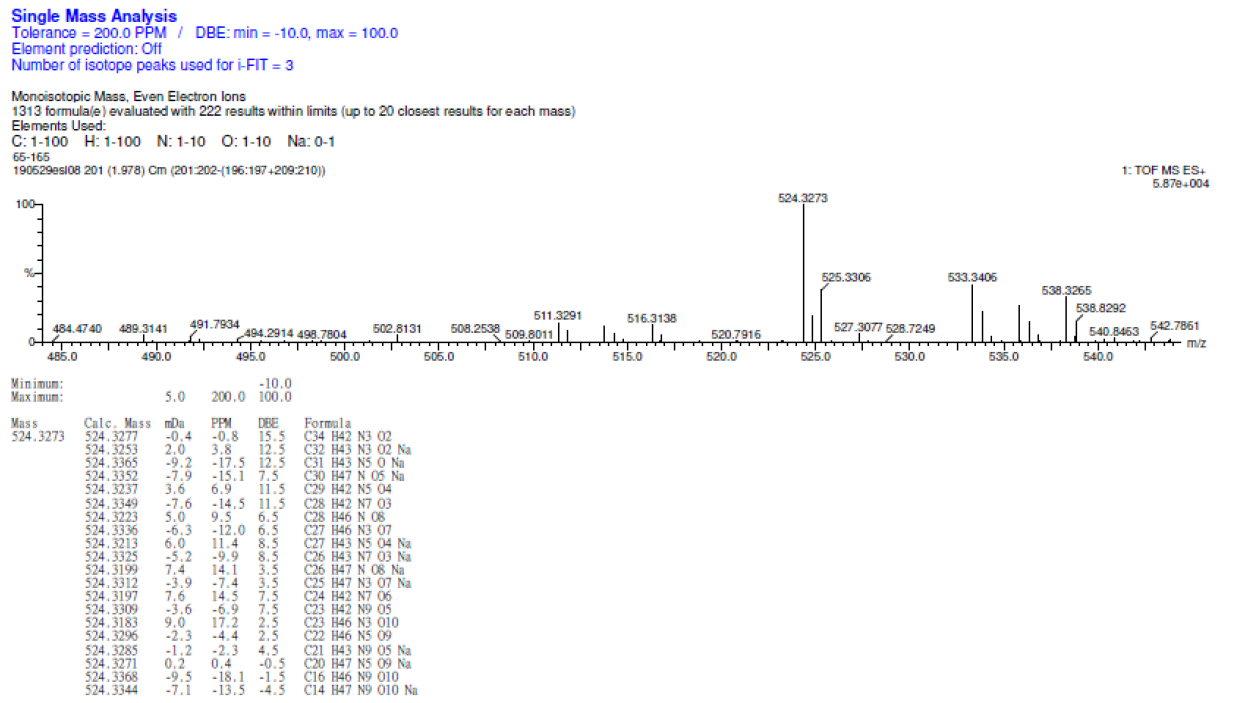


HRMS Data of Compound (cp2)


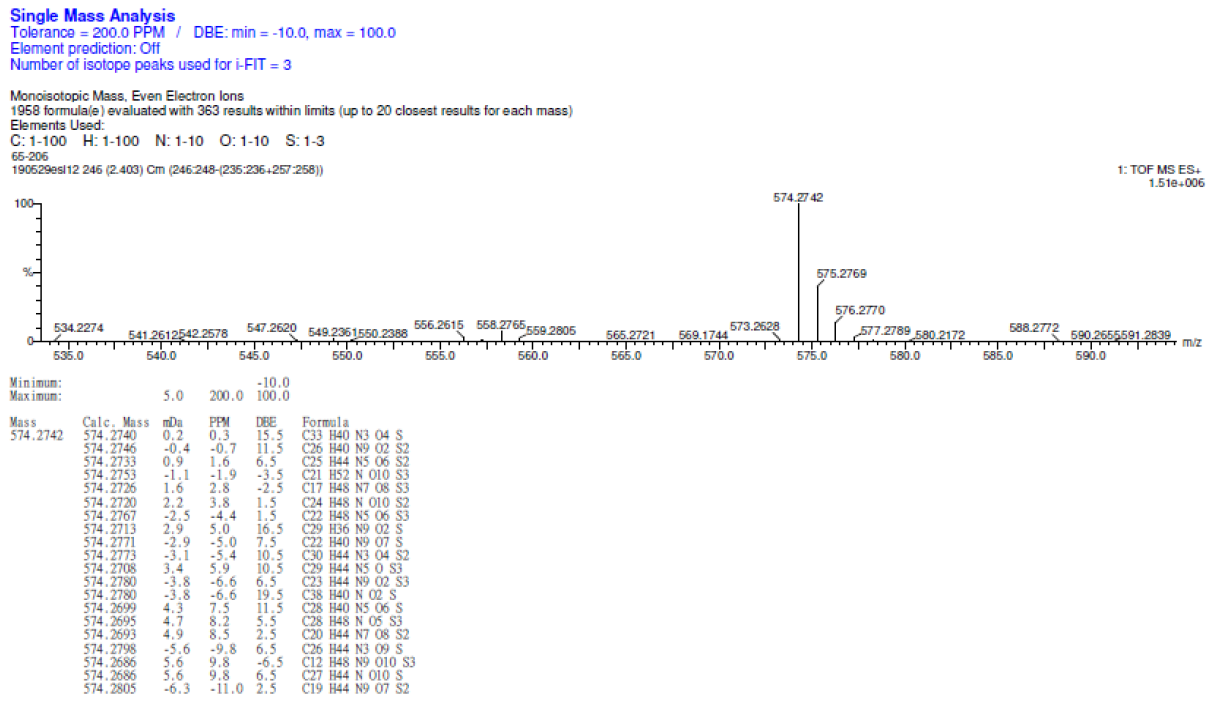


HRMS Data of Compound (cp3)


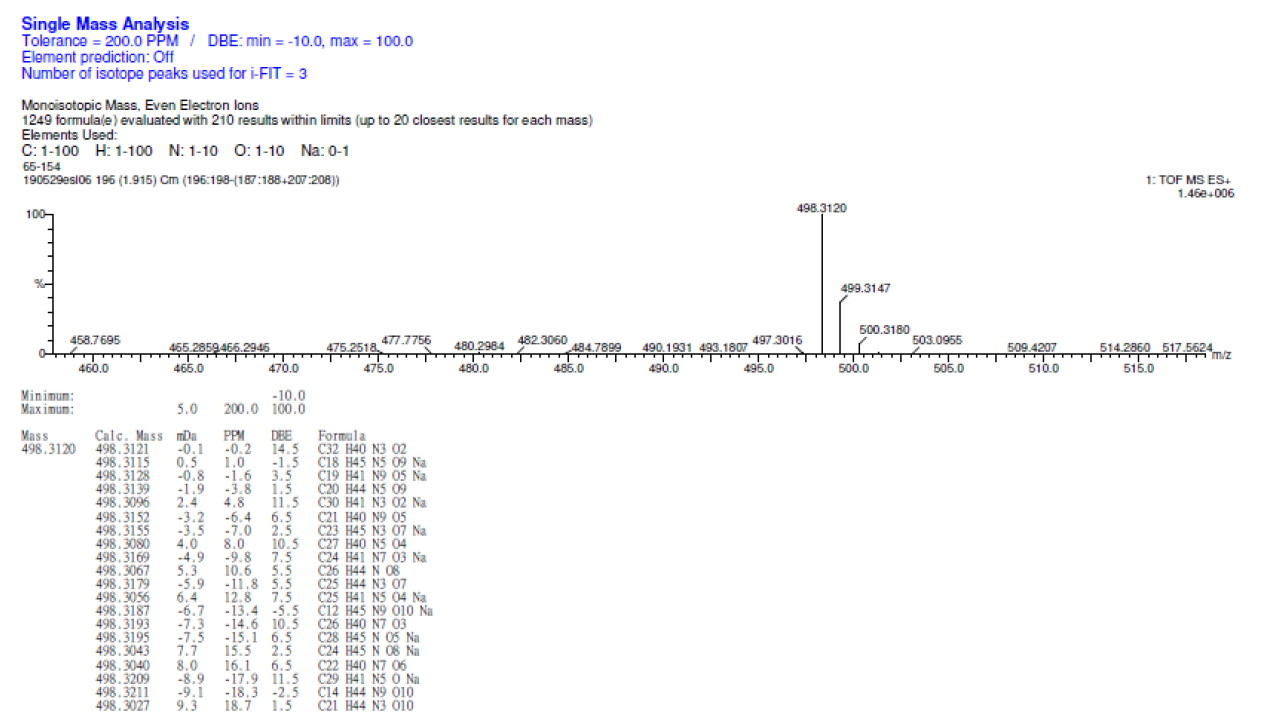


HRMS Data of Compound (cp4)


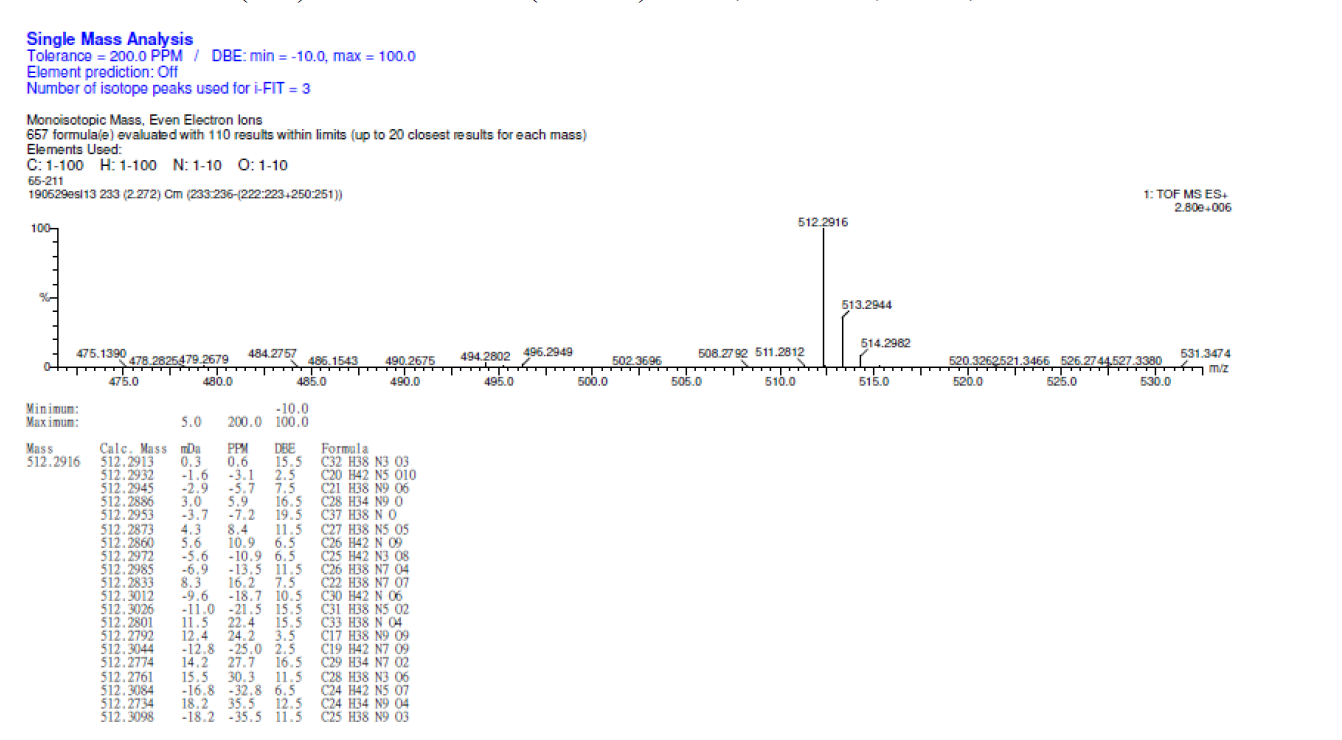


HRMS Data of Compound (cp5)


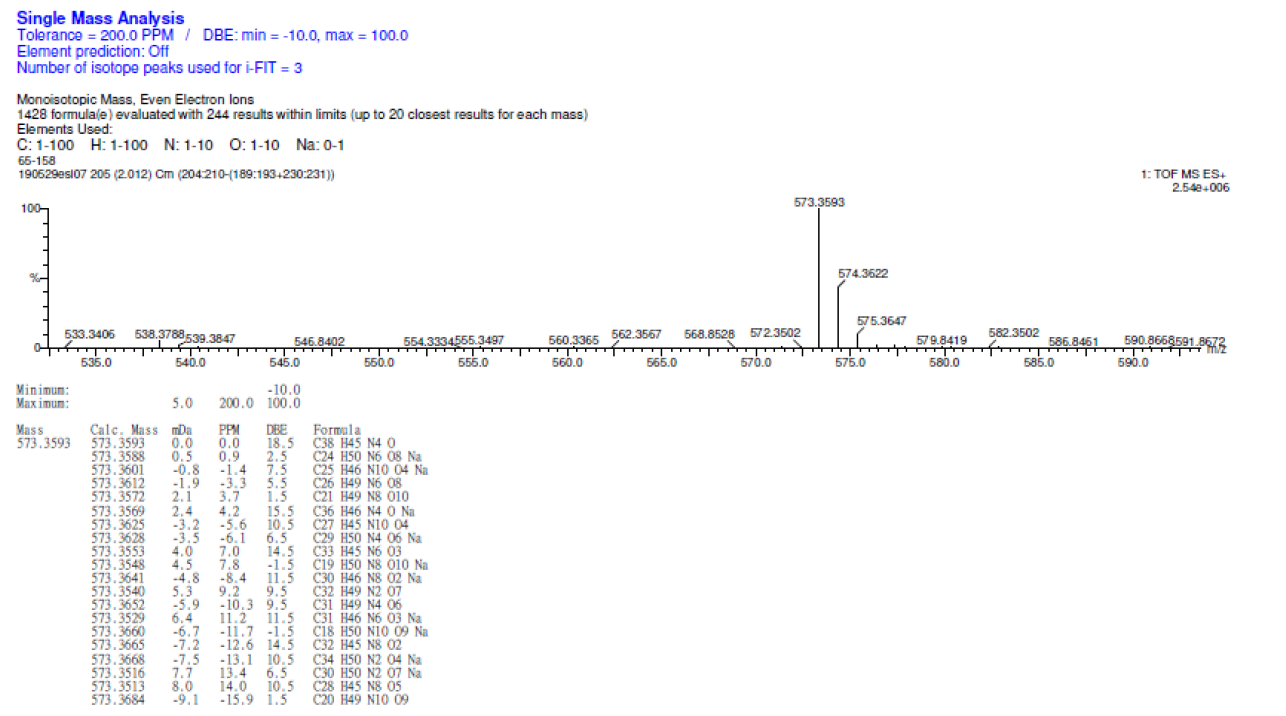


HRMS Data of Compound (cp6)


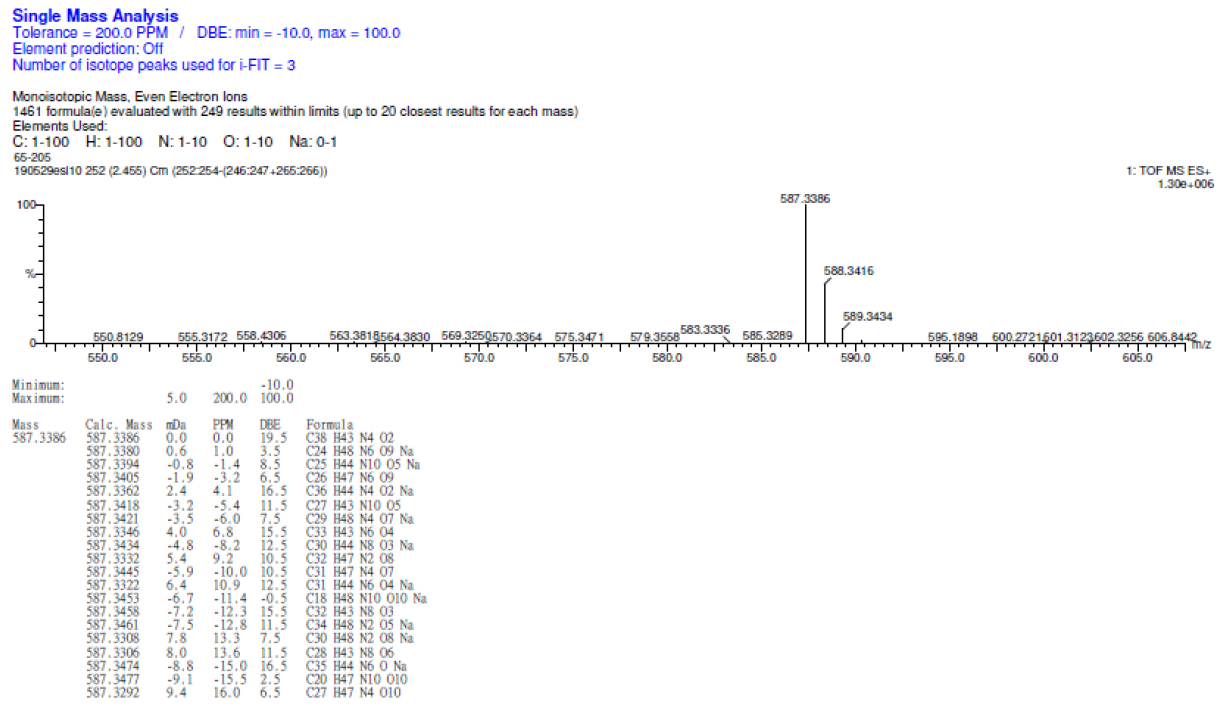


HRMS Data of Compound (cp7)


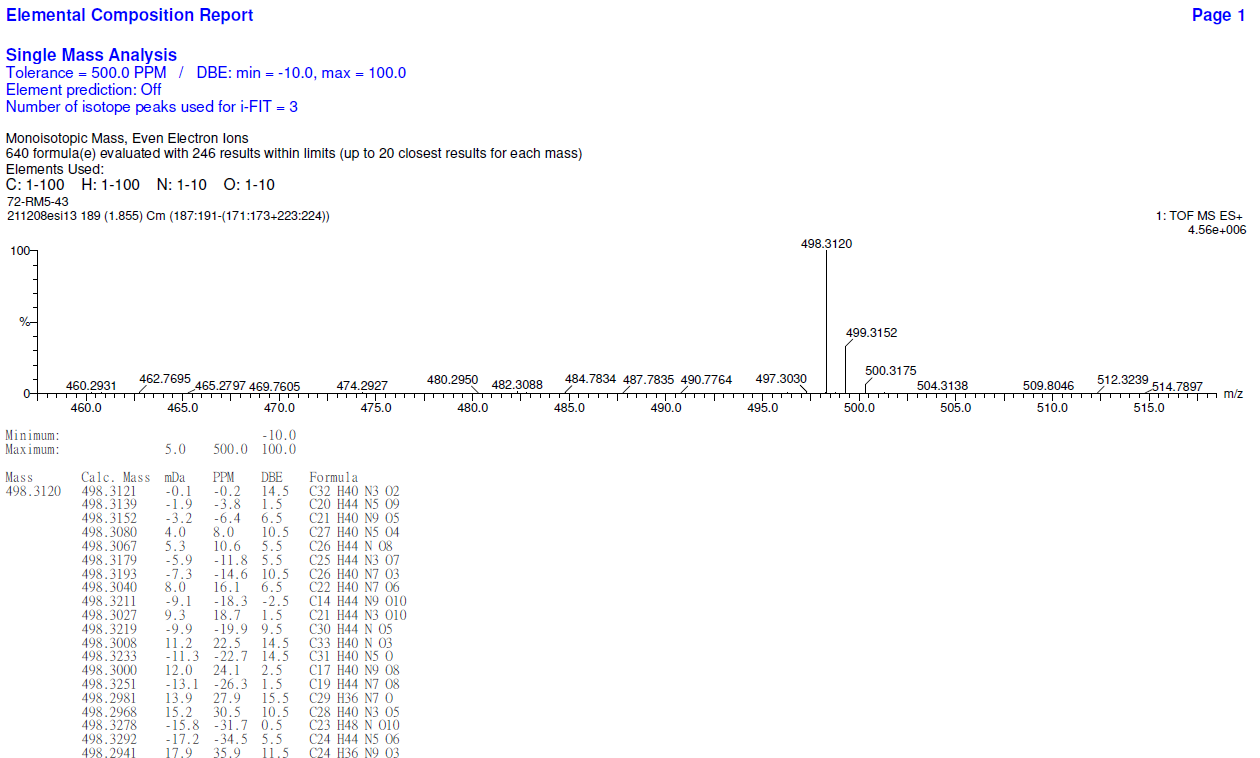


HRMS Data of Compound (cp8)


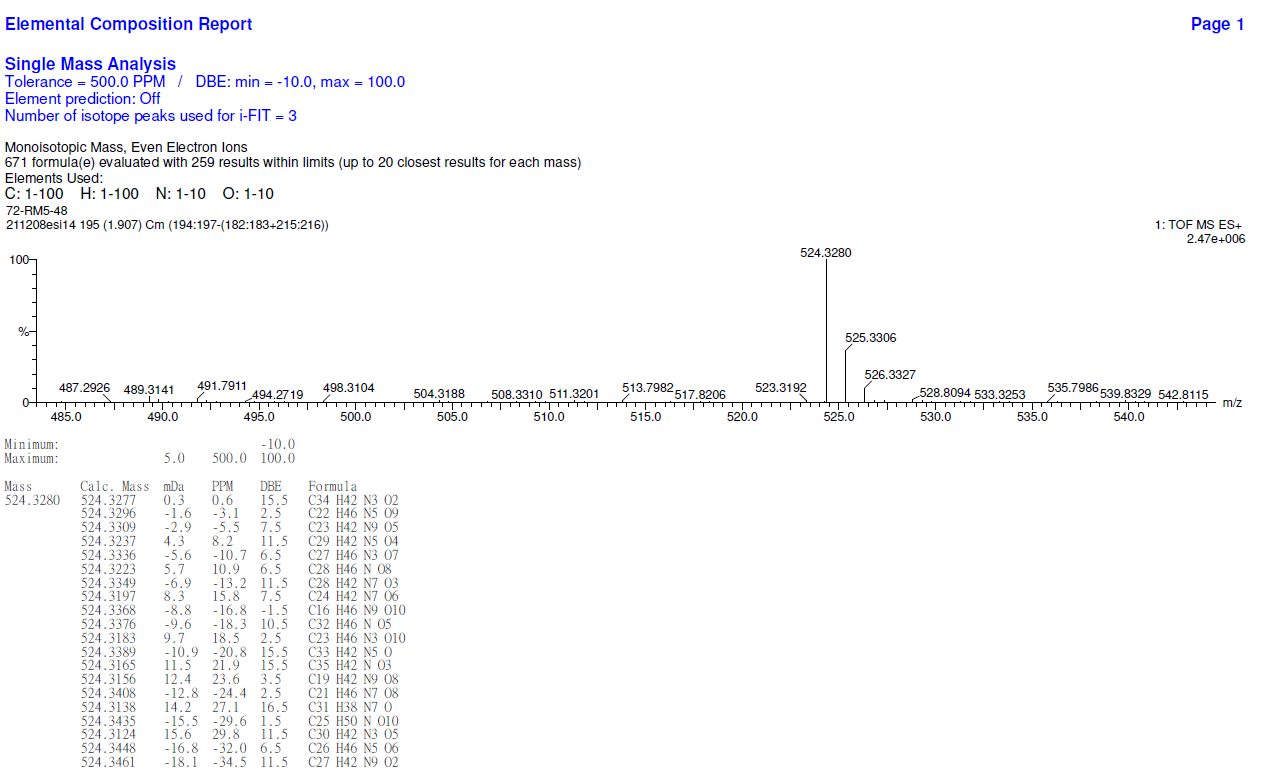


HRMS Data of Compound (cp9)


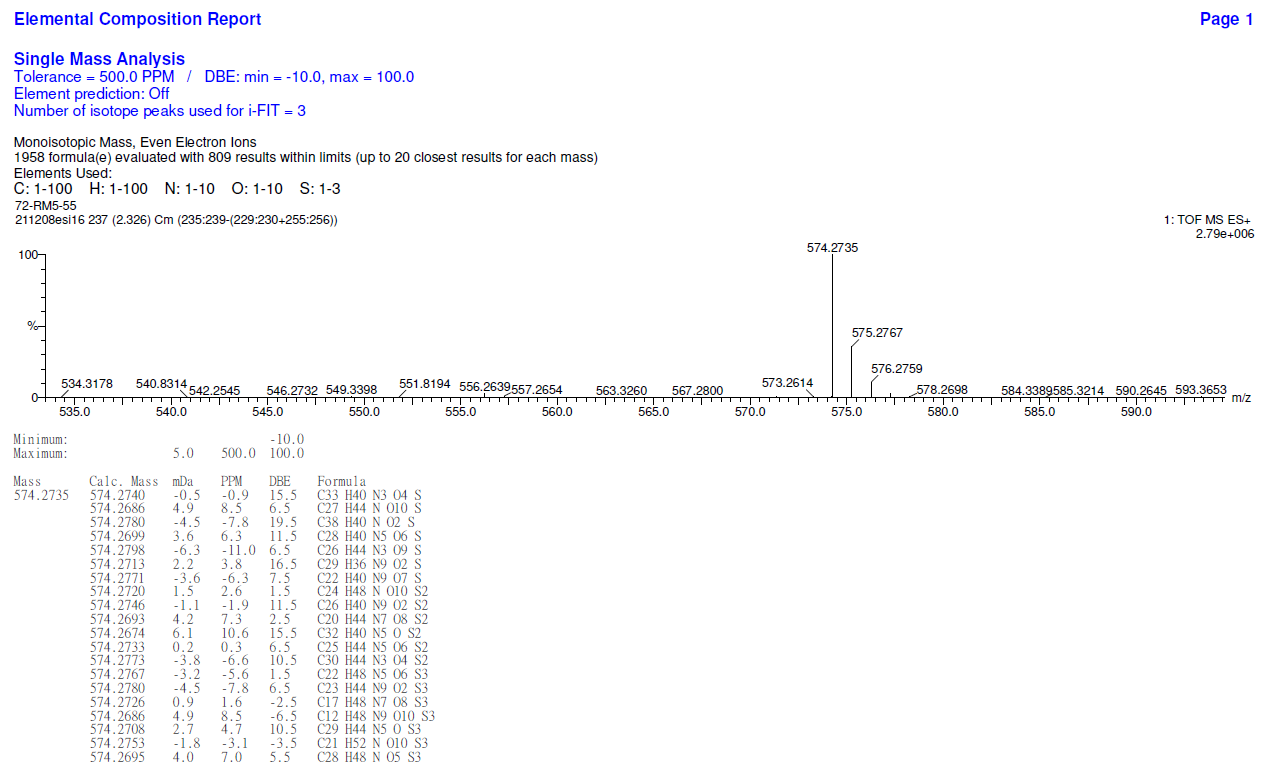


HRMS Data of Compound (cp10)


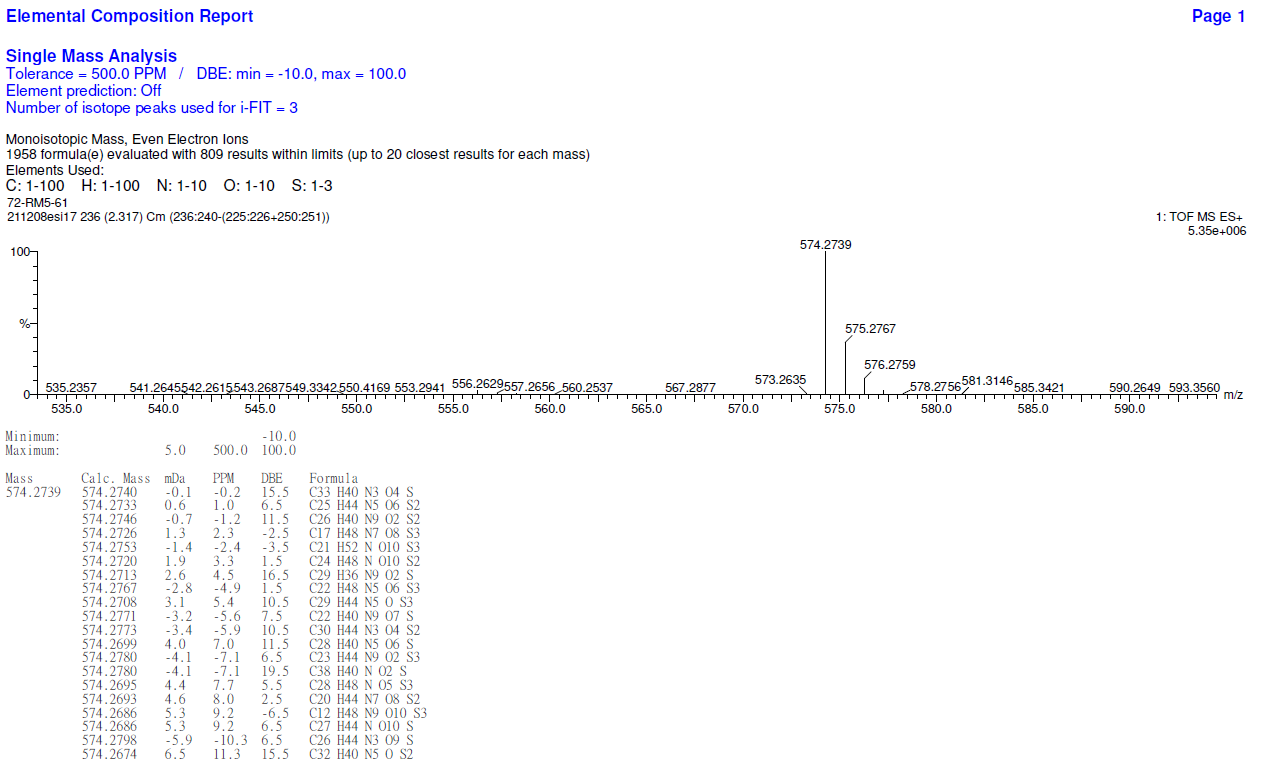


HPLC Purity Data of Compound (cp1)


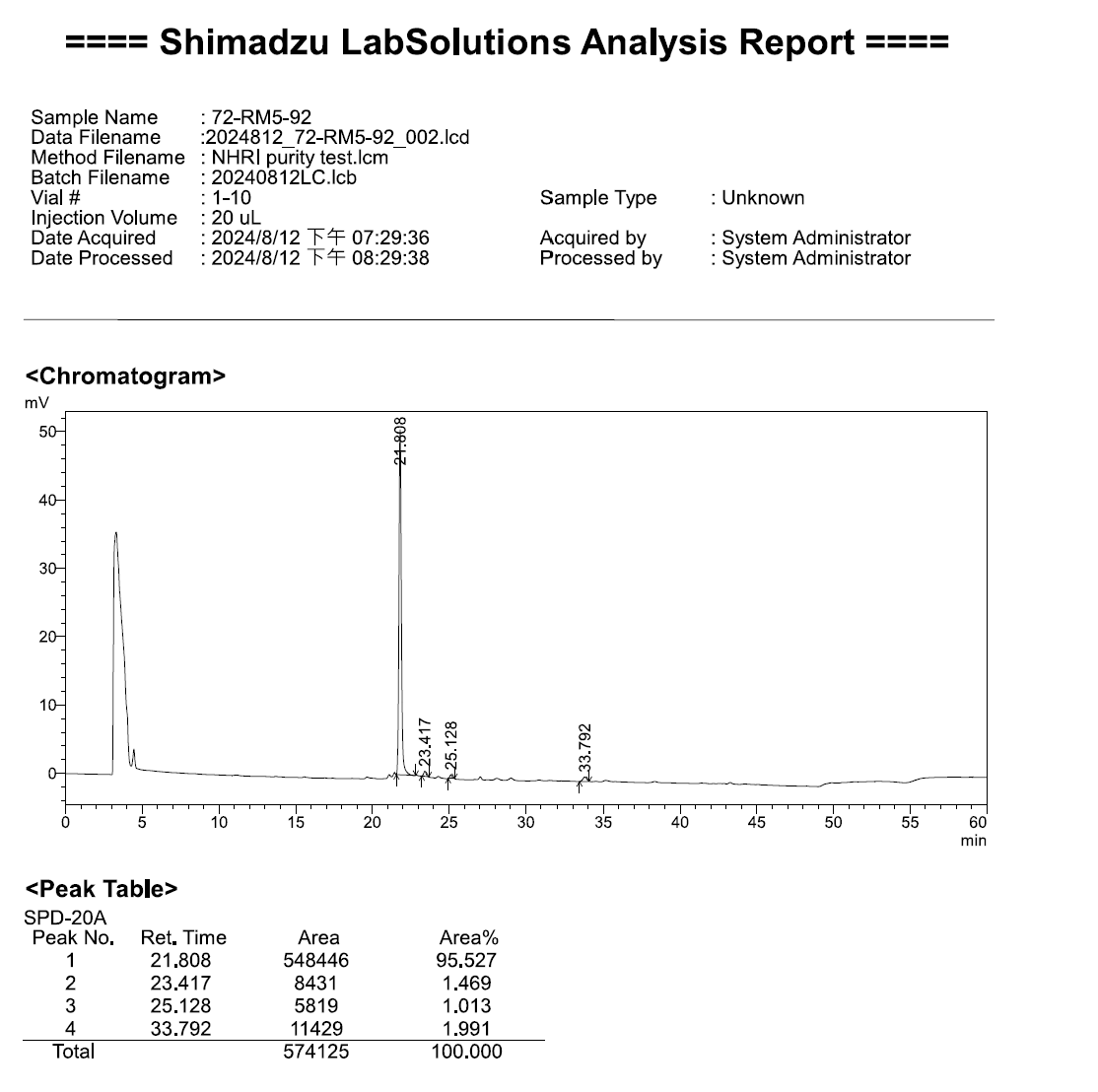


HPLC Purity Data of Compound (cp2)


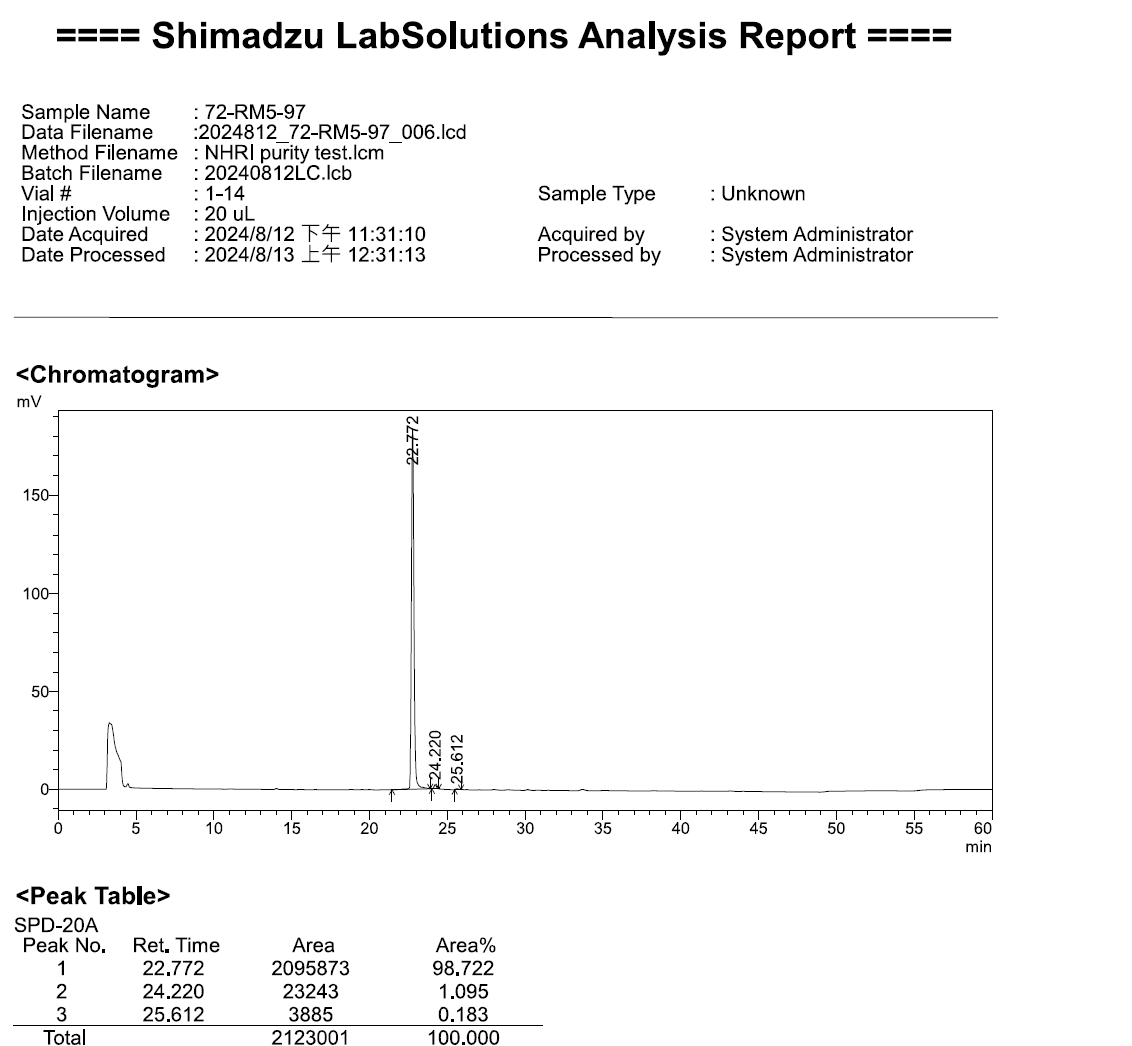


HPLC Purity Data of Compound (cp3)


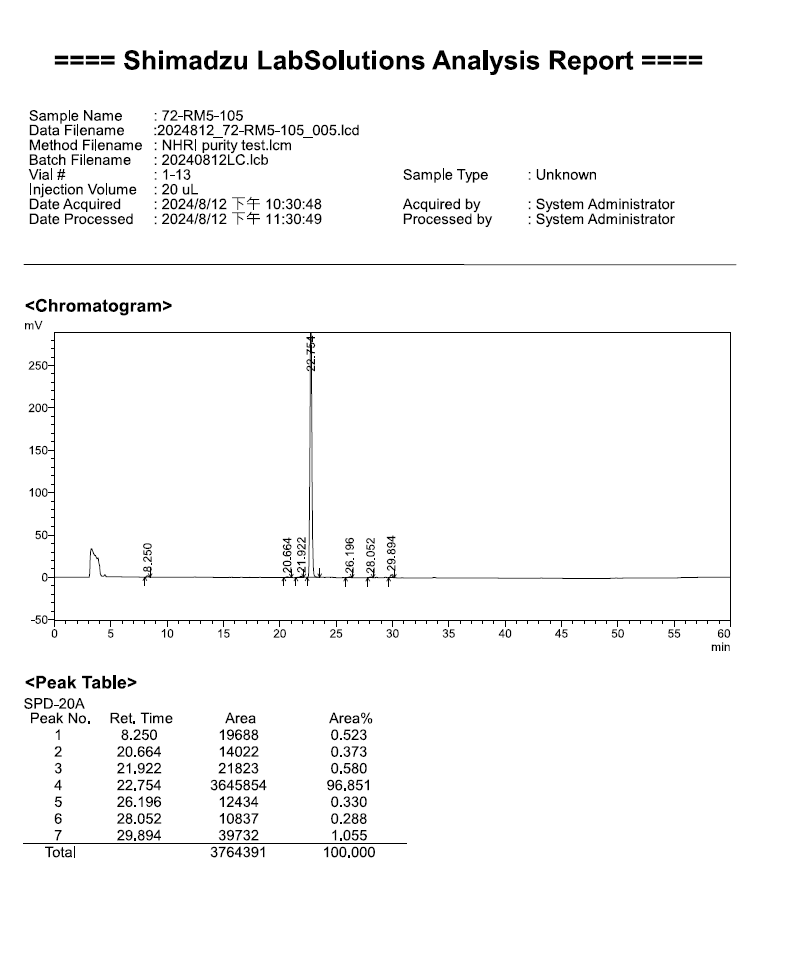


HPLC Purity Data of Compound (cp4)


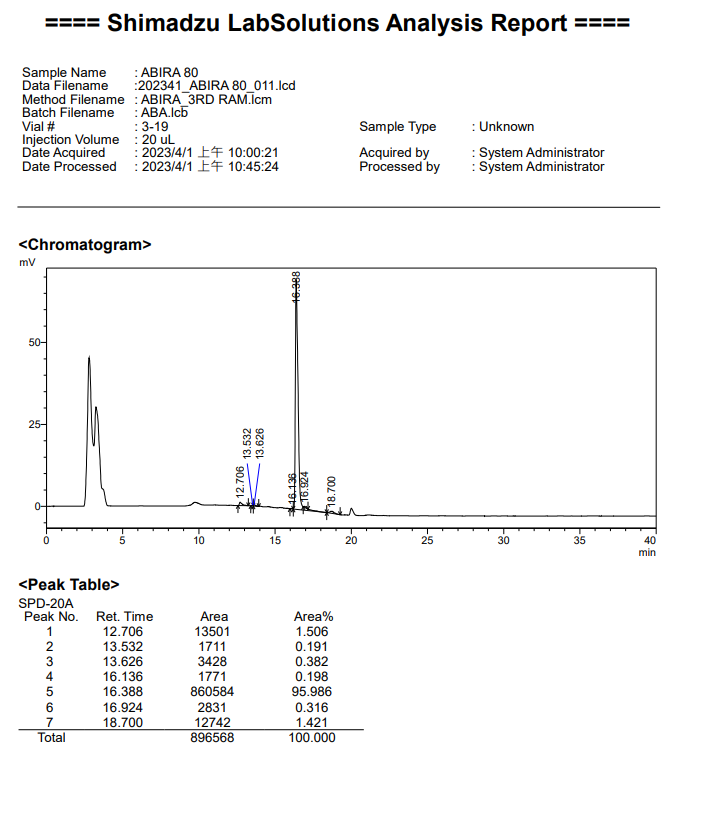


HPLC Purity Data of Compound (cp5)


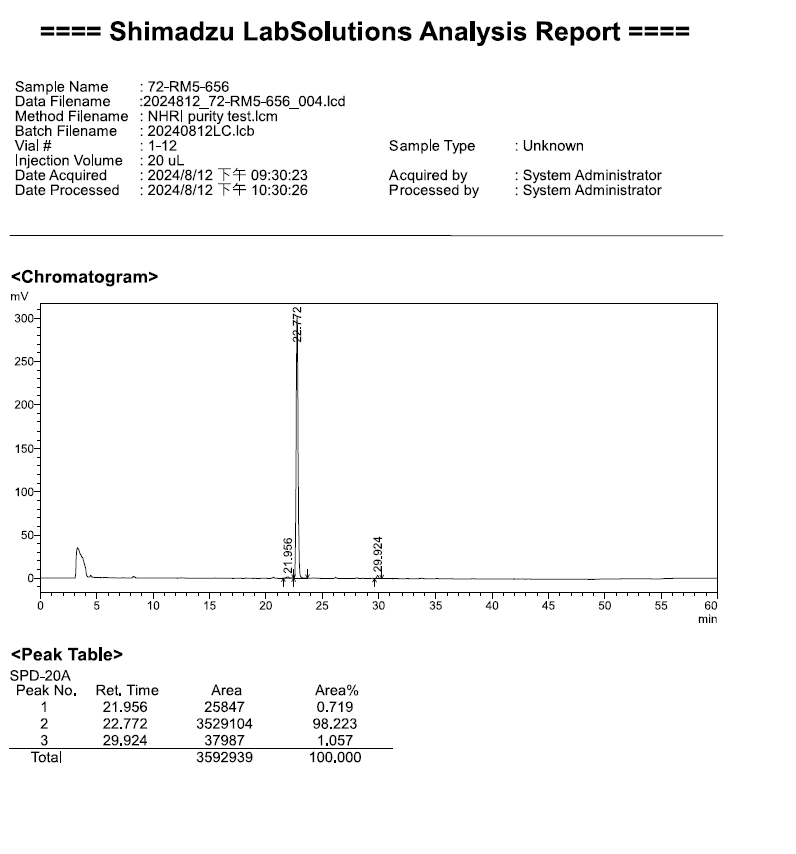


HPLC Purity Data of Compound (cp6)


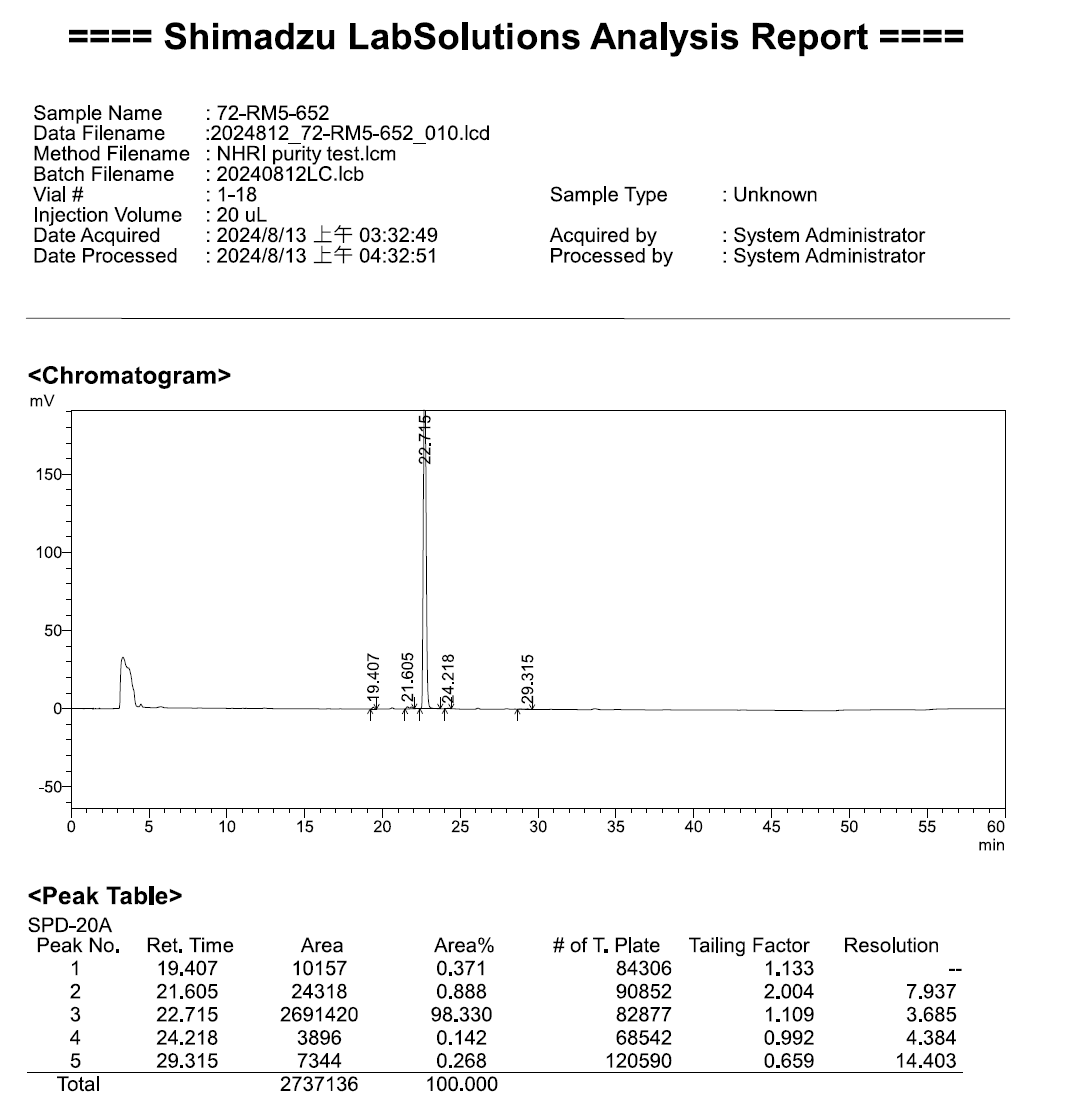


HPLC Purity Data of Compound (cp7)


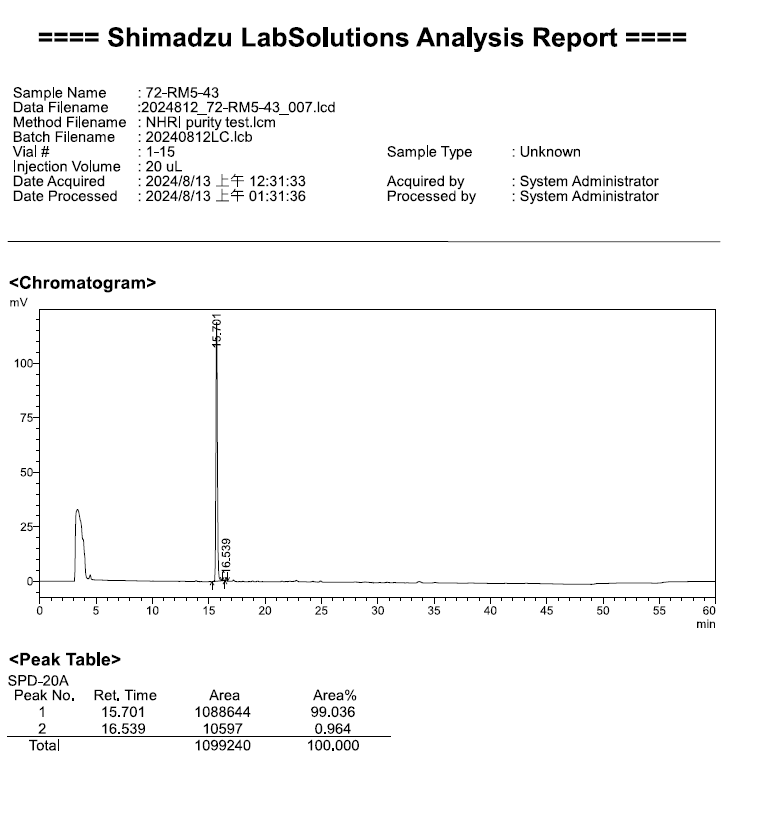


HPLC Purity Data of Compound (cp8)


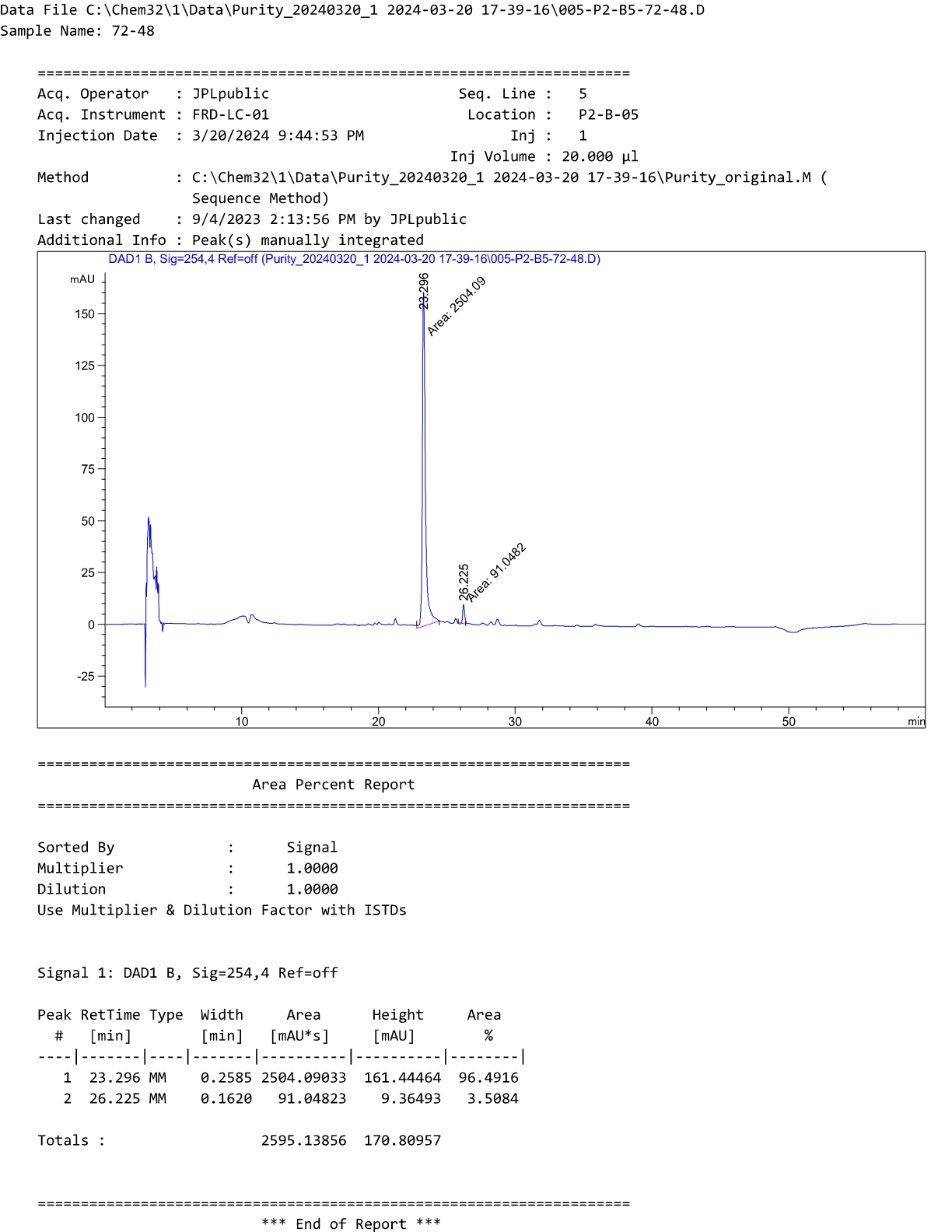


HPLC Purity Data of Compound (cp9)


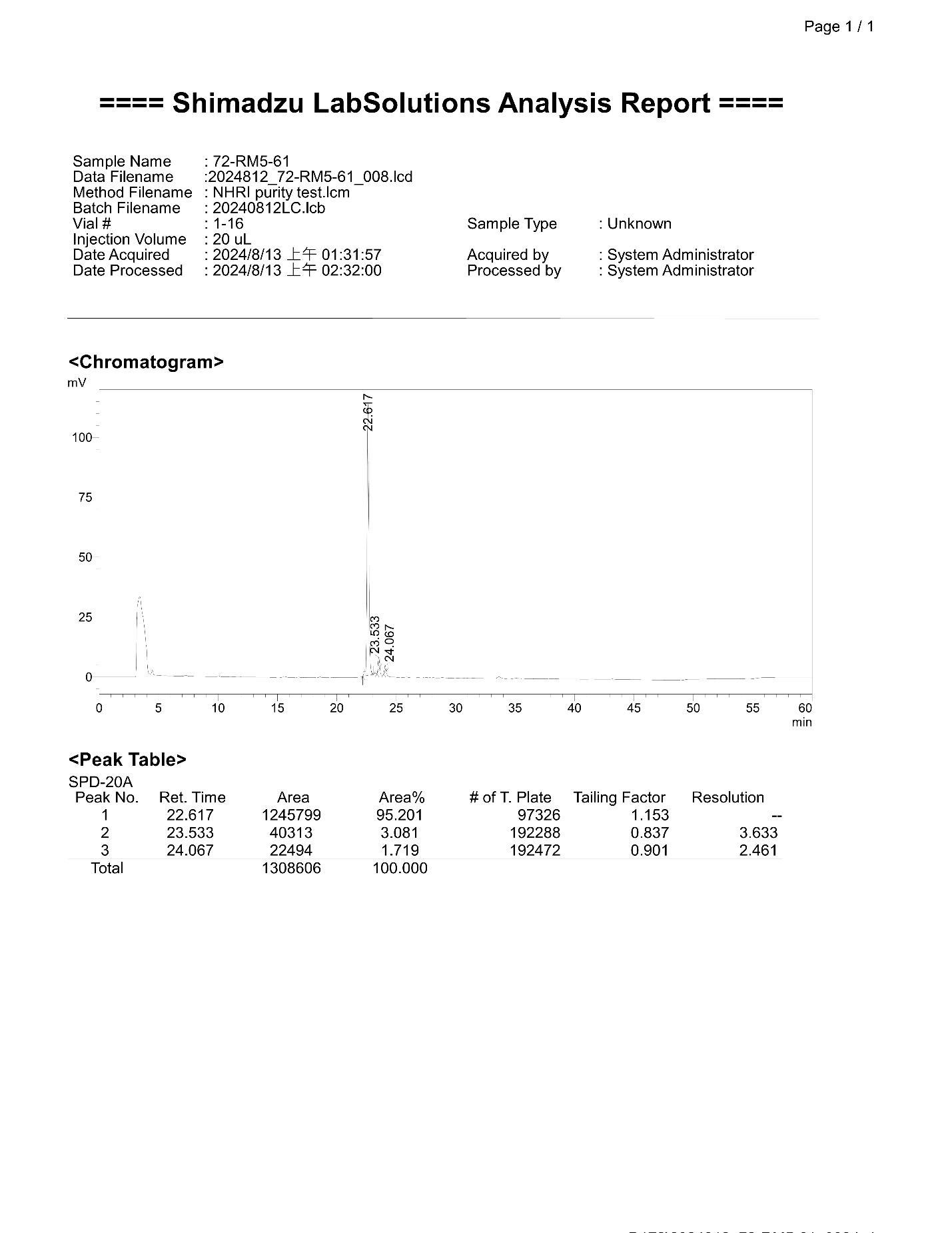


HPLC Purity Data of Compound (cp10)


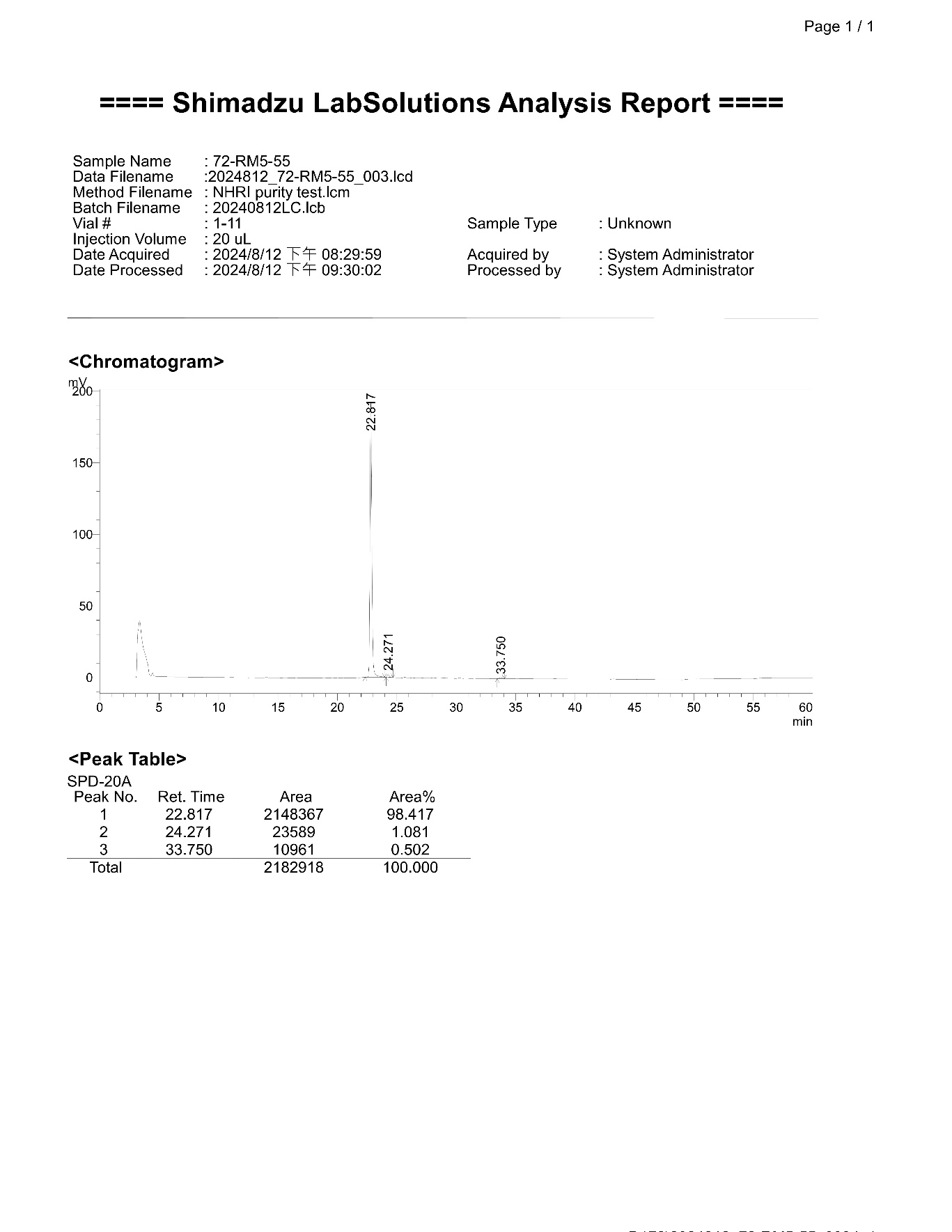

Supplement: Supplementary file 1 — Additional file 1. [file 12929_2026_1241_MOESM1_ESM.docx]
