# Supplementary material for: Dual suppression of stemness and redox adaptation in glioblastoma through filaggrin upregulation by an abiraterone-based HDAC inhibitor
Source: J Biomed Sci. 2026 Apr 6;33:38. doi: 10.1186/s12929-026-01241-2 (PMC13051497; doi:10.1186/s12929-026-01241-2)

**Supplementary materials**

**Method S1. Mycoplasma testing procedure**

Mycoplasma contamination was evaluated using the MycoStrip® detection kit (InvivoGen) according to the manufacturer’s instructions. Briefly, cell culture supernatant was collected from actively growing cultures and processed using the kit-provided sample preparation reagents. Samples were mixed with the Reaction Mix and incubated at 65°C for approximately 40 minutes to allow isothermal amplification of conserved mycoplasma 16S rRNA gene sequences. Following amplification, reactions were applied to immunochromatographic detection strips and results were visualized within 2 - 5 minutes. The presence of a single control band indicated a negative result, whereas two bands indicated a positive result for mycoplasma contamination.

**Method S2. TUNEL staining**

Paraffin-embedded tissue sections (5 μm) were deparaffinized in xylene and rehydrated through a graded ethanol series. Endogenous peroxidase activity was quenched by incubation in 0.3% hydrogen peroxide for 10 minutes. Apoptotic nuclei were detected using a commercial TUNEL assay kit (ab206386, Abcam) according to the instructions of the manufacturer, with Proteinase K treatment included to enhance tissue permeability and probe access. Colorimetric signal was developed using a DAB substrate, and sections were counterstained with methyl green prior to dehydration and mounting.

**Table S1.** **Description of the derivation source, selection steps, TMZ exposure and MGMT status, and mycoplasma testing of patient-derived Pt#3, Pt#3-R, and P1S cell lines**

|  | **Pt#3** | **Pt#3-R** | **P1S** |
| --- | --- | --- | --- |
| **Derivation source** | Pt#3 cells were isolated from the GBM tissue of a male patient who was treated and cared in Taipei Medical University Hospital (Taipei, Taiwan). The consent of the patient was obtained and approved by Taipei Medical University IRB protocols, No. 201006011 [1]. | Pt#3-R cells were generated by treating Pt#3 with 50 μM TMZ for 1 day and divided among 96-well plates (1 cell per well). Cells were subsequently incubated with 50 μM TMZ for 21 days, and surviving cells were cultured in the media containing 100 μM TMZ for additional 39 days. These cells were maintained in complete DMEM containing 50 μM TMZ for at least 60 days. TMZ resistance was confirmed by colony formation assay [2]. | PtR#1 (or P1S) TMZ-resistant GBM cells were purified from a female recurrent GBM patient exhibiting radio- and chemotherapeutic resistance. The patient was treated and cared in Taipei Medical University Hospital (Taipei, Taiwan). The consent of the patient was obtained and approved by Taipei Medical University IRB protocols, No. 201402018 [1]. |
| **Selection step** | The freshly resected tissues were digested by 0.05% collagenase type IV (Sigma-Aldrich, St. Louis, MO, USA) and 5 units/mL DNase I (Sigma-Aldrich) at 37 °C for 2 hrs. After removing undigested tissues by centrifugation, the supernatant was mixed with complete DMEM and transferred to 6-well plates. Cells were maintained in complete DMEM [1]. |  | P1S cell preparation process is identical to Pt#3 selection procedure [1]. |
| **TMZ exposure** | Pt#3 cells are cultured and maintained in DMEM without TMZ | Pt#3-R cells are cultivated and maintained in DMEM with 50 μM of TMZ | P1S cells are cultivated and maintained in DMEM with 50 μM of TMZ |
| **MGMT status** | Negative [1], [3] | Negative [1], [3] | Positive  The MGMT protein expression of P1S was identified through immunoblotting and shown in the revised Figure S1. |
| **Authentication** | Cell line Pt#3 DNA typing report is shown in the revised Figure S12. |  |  |
| **Mycoplasma testing** | The process to test Mycoplasma contamination is added in the revised Method S1. | | |
|  | 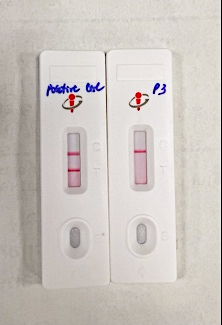 | 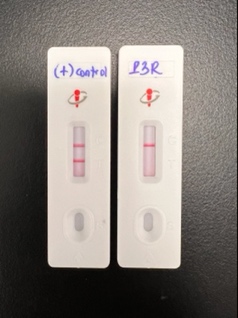 | 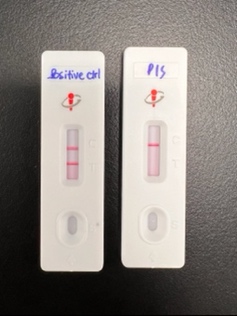 |
| **References** | [1], [3] | [1], [2], [3] | [1] |

**Table S2. Gene sequences for quantitative PCR and FLG overexpression using CRISPR-Cas9 approach**

| MGMT PCR | F-TCCAGCAAGAGTCGTTCACC, R-CAGTCCTCCGGAGTAGTTGC |
| --- | --- |
| Oct4 PCR | F-CTTGCTGCAGAAGT GGGTGGAGGAA  R-CTGCAGTGTGGGTTTCGGGCA |
| SOX-2 PCR | F-AAATGGGAGGGGTGCAAAAGAGGAG  R-CAGCTGTCATTTGCTGTGGGTGATG |
| FLG PCR | F-AGGAATACAGTCACGTGGCA, R-CTTCCGTGCTGAGAGTGTCT |
| MIR210HG PCR | F-GCTTGGTAGAGTGTCACGCC, R-CATCTGACCGAGCCAGTTTG |
| TEX19 PCR | F-GTCCTACCTCTACGCCTCCT, R-TGCCTCAGTGTGCTCCATC |
| GAPDH PCR | F-CCATCACCATCTTC CAGGAG, R-CCTGCTTCACCACCTTCTTG |
| MGMT PCR | F-TCCAGCAAGAGTCGTTCACC, R-CAGTCCTCCGGAGTAGTTGC |
| FLG knock-in | F-ACCGGGAATGAATCAGACCATCCCACA,  R-AAACTGTGGGATGGTCTGATTCATTCC |

**Table S3. Antibodies for western blotting**

| **Antibody** | **Company** | **Catalog** | **Dilution** |
| --- | --- | --- | --- |
| Anti-Histone H3 | iReal Biotechnology | IR4-10 | 1:1000 |
| Anti-Acetyl-Histone H3 | GeneTex | GTX122148 | 1:1000 |
| Anti-Acetyl-Histone H3K18 | ABclonal | A7257 | 1:1000 |
| Anti-Acetyl-Histone H3K27 | ABclonal | A7253 | 1:1000 |
| Anti-Histone H4 | Merck Millipore | 04-858 | 1:1000 |
| Anti-Acetyl-Histone H4 | Merck Millipore | 06-598 | 1:1000 |
| Anti-Acetyl-Histone H4K5 | ABclonal | A19525 | 1:1000 |
| Anti-Acetyl-Histone H4 (Lys12)  (Anti-Acetyl-Histone H4K12) | Cell Signaling | 2591 | 1:1000 |
| Anti-MGMT | ABclonal | A0052 | 1:1000 |
| Anti-Oct4 | GeneTex | GTX100622 | 1:1000 |
| Anti-SOX-2 | GeneTex | GTX101507 | 1:1000 |
| Anti-CD133 | Proteintech | 18470-1-AP | 1:1000 |
| Anti-FLG | Thermo Fisher Scientific | PA5-116911 | 1:1000 |
| Anti-Acetylated tubulin | Merck Millipore | T7451 | 1:1000 |
| Anti-GAPDH | GeneTex | GTX627408 | 1:20000 |
| Anti-β-actin | GeneTex | GTX629630 | 1:20000 |
| Anti-⍺-tubulin | Sigma Aldrich | T9026 | 1:20000 |

**Table S4. IC50 values of abiraterone derivatives on mouse CT-2A, human U87MG, and patient-derived TMZ-resistant GBM cell lines**

| **Compound** | **CT-2A (MGMT-)** | **U87MG (MGMT-)** | **P1S (MGMT+)** |
| --- | --- | --- | --- |
| **1** | 4.55 ± 0.31 μM | 7.50 ± 0.46 μM | 5.66 ± 0.52 μM |
| **2** | 2.46 ± 0.2 μM | 4.08 ± 0.6 μM | 2.14 ± 0.17 μM |
| **3** | 4.95 ± 0.66 μM | 13.47 ± 0.76 μM | 7.55 ± 0.52 μM |
| **4** | 15.09 ± 1.13 μM | 43.3 ± 2.56 μM | 21.68 ± 2.2 μM |
| **5** | 12.35 ± 8.11 μM | > 90 μM | > 90 μM |
| **6** | 7.64 ± 2.74 μM | > 90 μM | > 90 μM |
| **7** | insoluable in DMSO | | |
| **8** | 0.74 ± 0.04 μM | 1.99 ± 0.16 μM | 2.139 ± 0.09 μM |
| **9** | 2.02 ± 0.11 μM | 3.73 ± 0.95 μM | 2.23 ± 0.14 μM |
| **10** | 0.59 ± 0.13 μM | 2.7 ± 0.18 μM | 1.89 ± 0.12 μM |

**Table S5. The hazard ratios and 95% confidance intervals among groups of the animal experiments.**

|  | **Hazard ratio (HR)** | **95% confidence interval (95% CI)** |
| --- | --- | --- |
| **Figure 7D.**  Cp8 5 mg/kg *vs.* DMSO | 0.70 | 0.26 - 1.88 |
| **Figure 7D.**  Cp8 10 mg/kg *vs.* DMSO | 0.37 | 0.11 - 1.20 |
| **Figure 7K.**  Cp8 10 mg/kg *vs.* SAHA 10 mg/kg | 0.32 | **0.08 - 1.40** |

**Table S6. Pharmacokinetic parameters of cp8 in plasma and brain tissue after a 2 mg/kg IV dose (n=4).**

| **Cp8** | **Plasma samples**  **(n=4)** | **Brain samples**  **(n=4)** |
| --- | --- | --- |
| Dosage | 2 mg/kg | 2 mg/kg |
| C_o_ (µg/mL) | 0.23 ± 0.06 | 0.01 ± 0.01 |
| *T*_1/2_ (hr) | 0.48 ± 0.18 | 0.46 ± 0.39 |
| AUC_0-last_ (hr*µg/mL) | 0.14 ± 0.04 | 0.03 ± 0.01 |
| AUC_0-inf_ (hr*µg/mL) | 0.17 ± 0.05 | 0.08 ± 0.05 |
| *V*d (L/kg) | 30.10 ± 15.84 | 126.66 ± 89.37 |
| *Cl* (L/hr/kg) | 12.60 ± 3.21 | 35.33 ± 21.41 |

**Table S7. HDAC isoform inhibition of compound cp8**

|  | **IC50 (μM)** | | | |
| --- | --- | --- | --- | --- |
|  | **HDAC1** | **HDAC2** | **HDAC6** | **HDAC8** |
| **Testing concentration (μM)** | 10 | 10 | 10 | 10 |
| **Cp8** | 0.359 | 0.751 | 0.175 | 3.93 |
| **Trichostatin A** | 0.00208 | 0.00629 | 0.00201 | 0.456 |

**Figure S1.**

**GAPDH**

**MGMT**

**T98G**

**Pt#3**

**Pt#3-R**

T98G: positive control


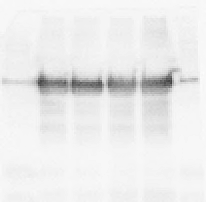


**P1S**


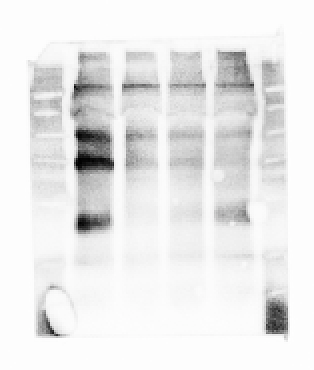


MGMT expression of patient-derived Pt#3, Pt#3-R, and P1S cells compared to positive control T98G, a MGMT-positive GBM cell line.

**Figure S2.**

The antiproliferative effect of cp8 against multiple GBM cell lines, following 72-hour exposure to cp8 using MTT assay.

**Figure S3.**

**A.**


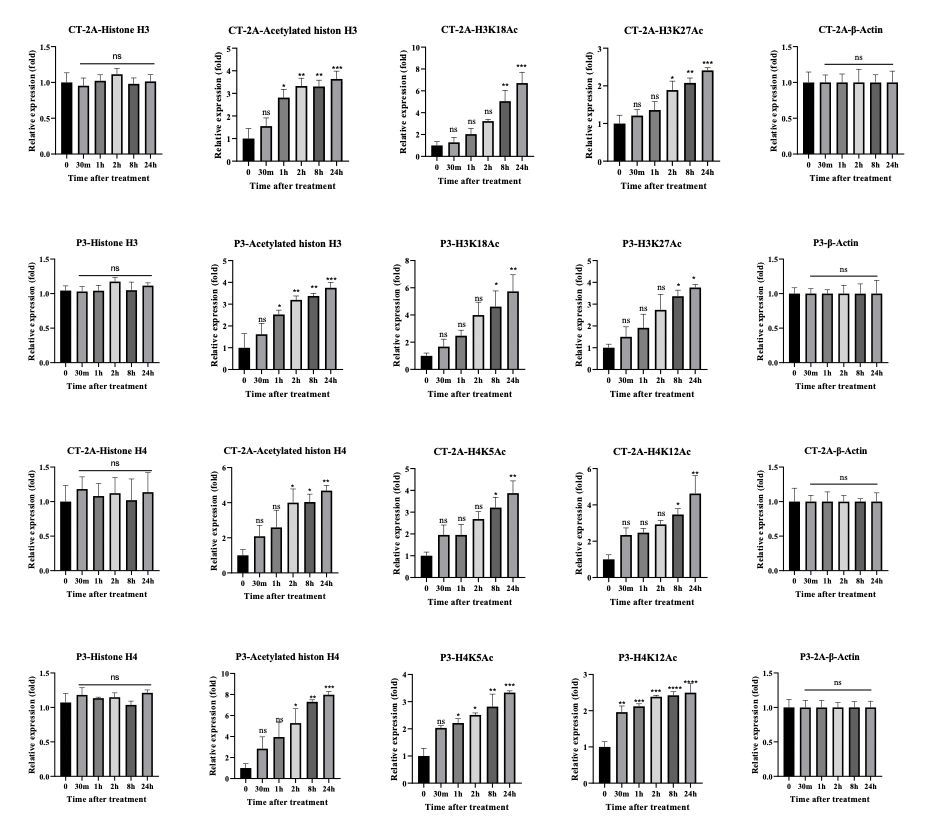


**B.**

| **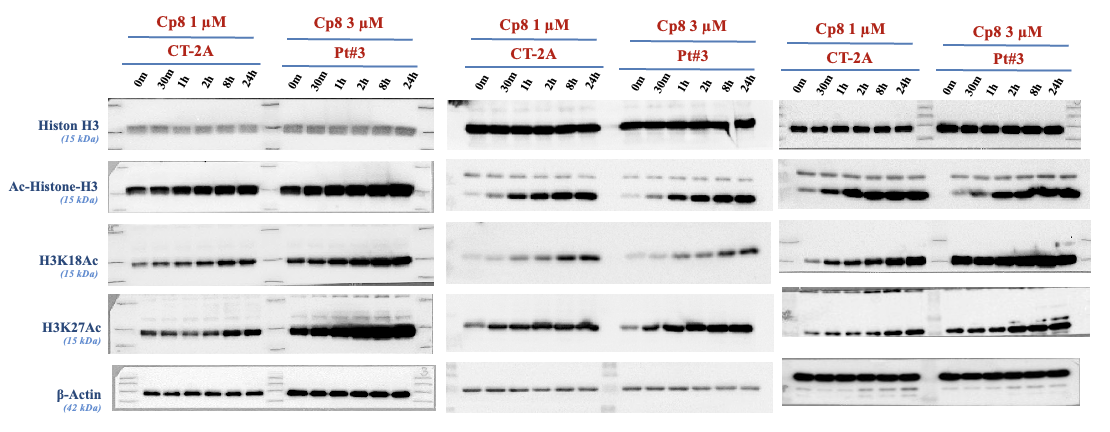** |
| --- |
| **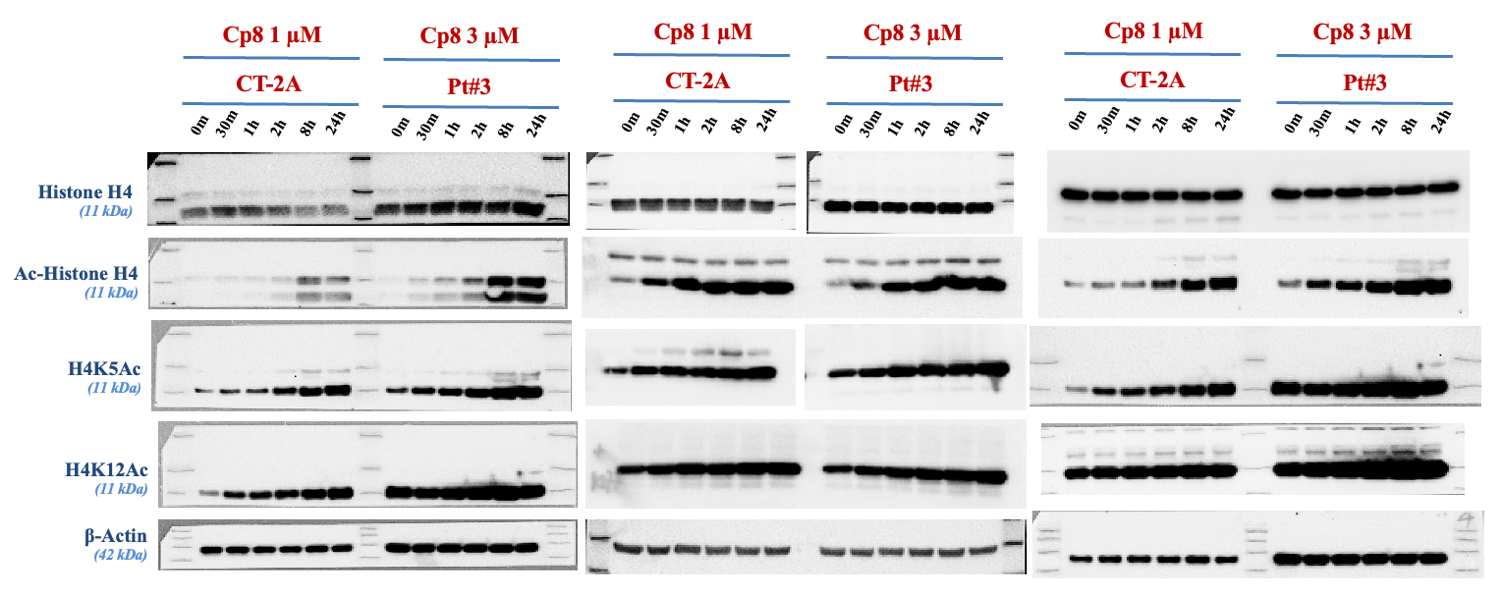** |

**Alterations in histone acetylation-related proteins following cp8 treatment.** A. Densitometric quantification of proteins normalized to total H3/H4 and the loading control β-actin. B. Uncropped full-length immunoblots corresponding to the quantified proteins.

The changes of proteins normalised to the total form of H3 or H4, and the internal control protein β-actin were quantified using ImageJ software. For each sample, signals were normalized to β-actin as the loading control, using the mean of three β-actin measurements as the normalization reference. Quantified data (triplicate) were plotted using GraphPad Prism, and statistical significance was evaluated by one-way ANOVA, with p < 0.05 considered statistically significant.

**Figure S4.**


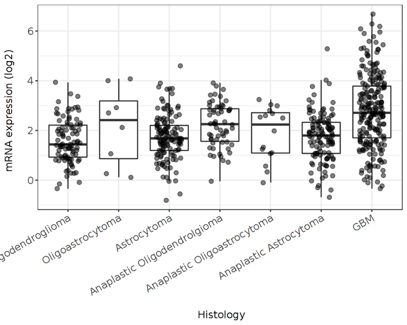


**MIR210HG**


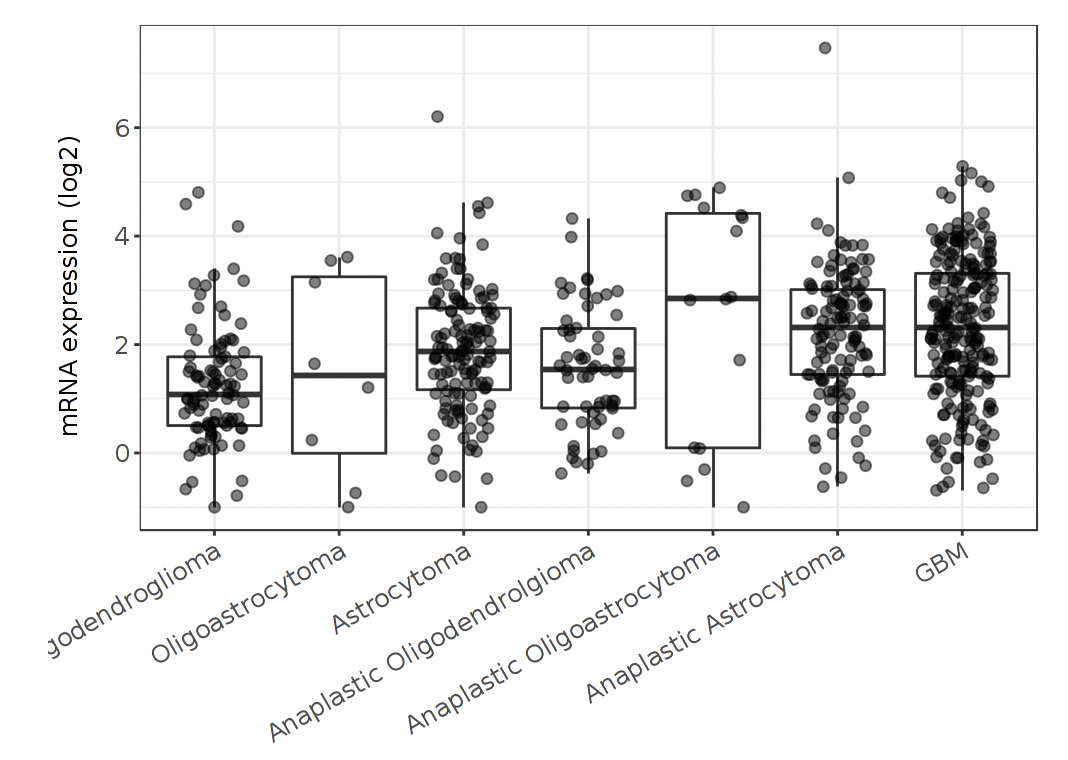


**MILR1**


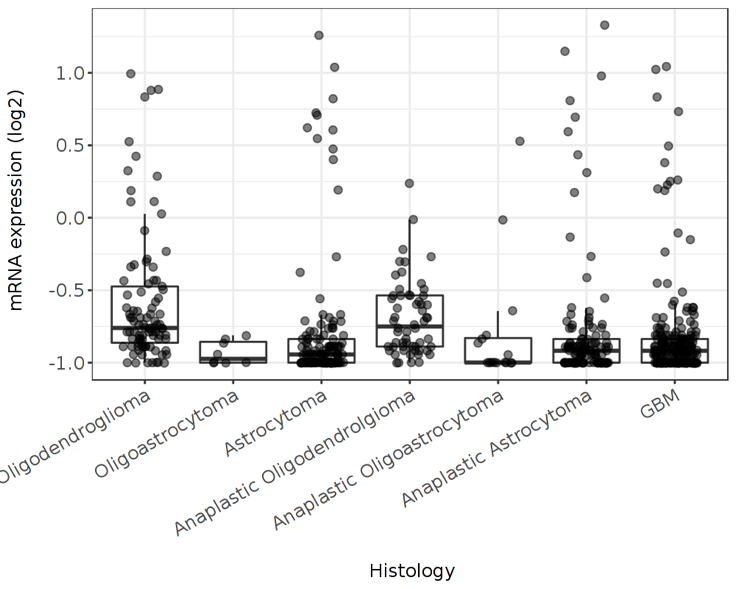


**TEX19**

**A.**


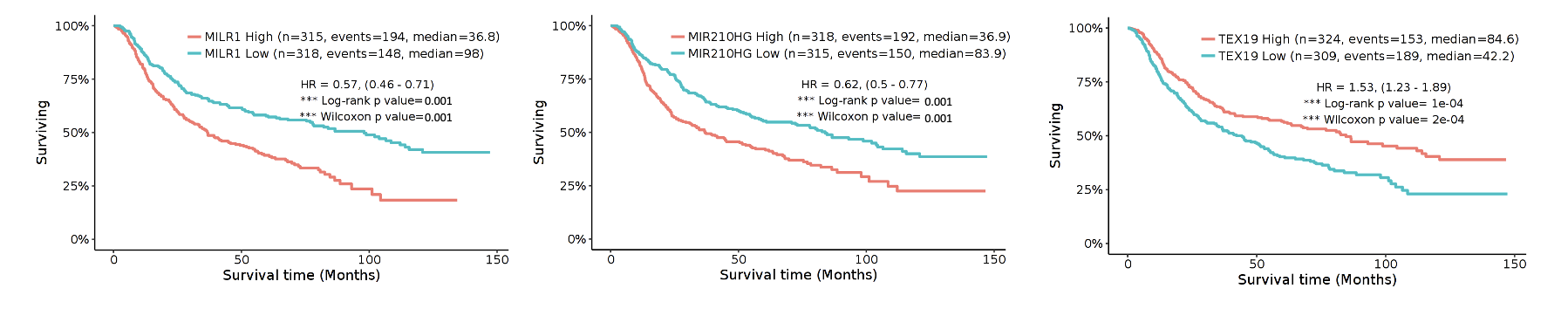


**C.**

**B.**

**The expression of cp8-regulated gene.** A. The mRNA expression of certain genes that are modulated by cp8, including MILR1, MIR210HG, and TEX19 from TCCA and CGGA databases. B. The correlation between gene levels and clinical outcomes from TCCA and CGGA databases. C. The mRNA expression of cp8-influenced genes in wild-type and TMZ-resistant GBM cells at different concentrations were validated using qPCR. Mean ± S.E.M of triplicate experiments. *, P < 0.05; **, P < 0.01; ***, P < 0.001.

**Figure S5.**

| **Gene** | **TGGA_GBM** | **CGGA** | **GEPIA** |
| --- | --- | --- | --- |
| **PMEL** | 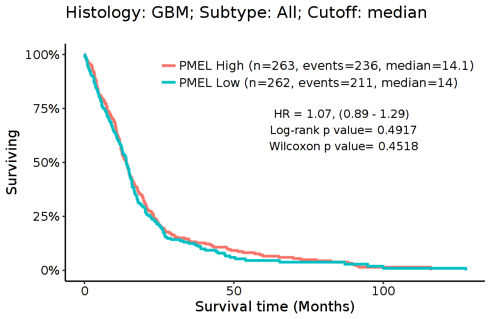 | 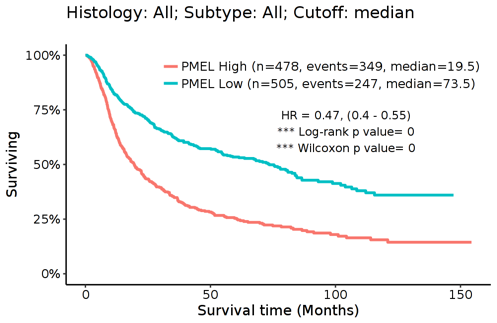 | 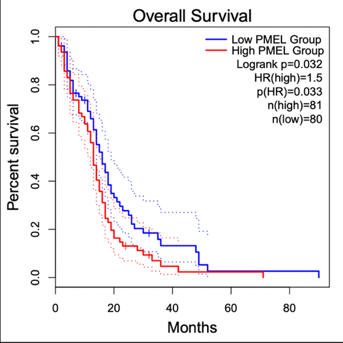 |
| **ANO3** | 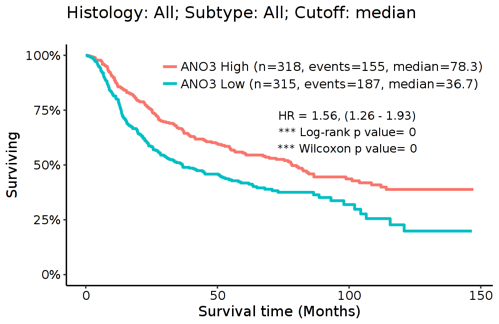 | 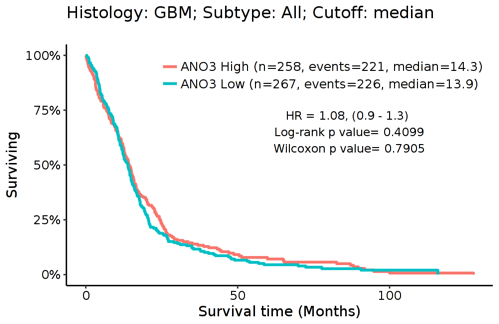 |  |
| **DCN** | 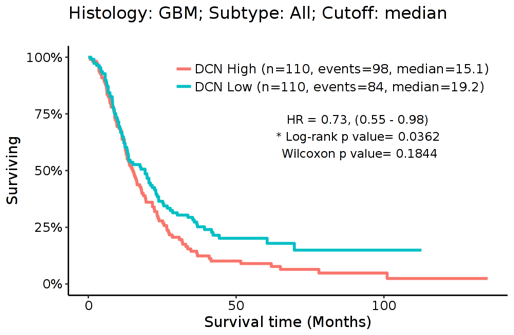 | 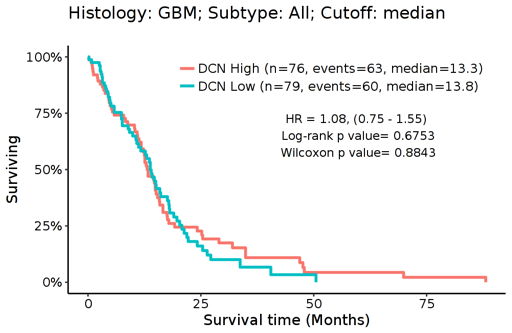 |  |

**Validation of gene expression of PMEL, ANO3, and DCN in GBM cells.** A. Gene alterations after treatment with cp8, assessed by qpcr. B. Correlation between mRNA expression and patient survival outcome from the TCGA and CGGA databases. *, P < 0.05; **, P < 0.01; ****, P < 0.0001.

**Figure S6.**

**SAHA (µM)**

**U87MG**

**72 hrs**

**0**

**2**

**5**

FLG


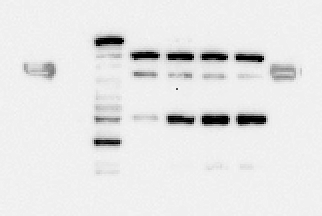


β-Actin


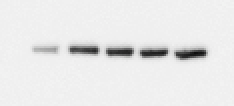


FLG expression of U87MG cells was detected by western blotting when the cells were treated with SAHA, a pan-HDAC inhibitor, at multiple doses for 72 hrs.

**Figure S7.**

**B.**

**A.**

**The sensitivity towards TMZ of FLG-overexpressed GBM cells.** CRISPR-Cas9 and plasmid transfection were used to overexpress FLG in GBM cells, which were then exposed to 600 µM TMZ for 96 hours. A. The cell viability assay and B. The active caspase-3 determination were conducted to assess the effect of TMZ on FLG-enhanced GBM cells.

**Figure S8.**

**Cp8 (µM)**

**72 hrs**

**T98G**

**0**

**0.5**

**1**

**3**

⍺-Tubulin

GAPDH

MGMT


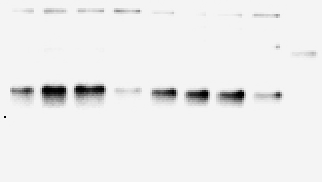

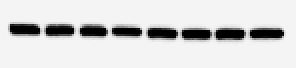

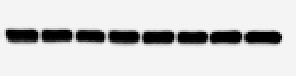


**B.**

**A.**

**The suppressive ability of cp8 on MGMT expression.** A. MGMT protein, and B. mRNA expression of MGMT-positive T98G cells when the cells were treated with cp8 at different doses for 72 hrs using immunoblotting and qPCR analysis, respectively.

**Figure S9.**

**A.**

**B.**


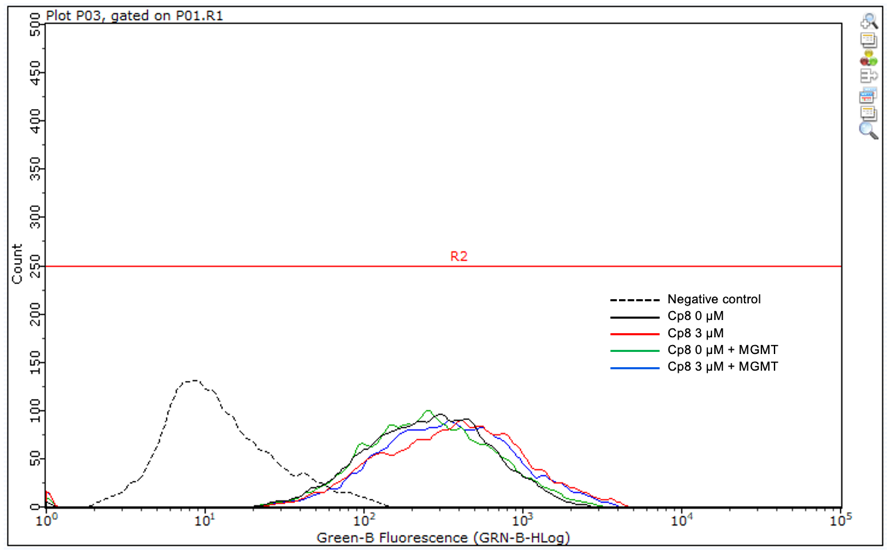


**The modulation of MGMT status on the inhibitory effect of cp8 on GBM cells.** A. MGMT overexpression was performed by MGMT transfection into Pt#3, followed by the cell viability assay after cells were treated with cp8 at different doses for 72 hours. B. ROS production in Pt#3 and MGMT-overexpressed Pt#3 cells after exposure to cp8 was measured using CellROX analysis.

**Figure S10.**


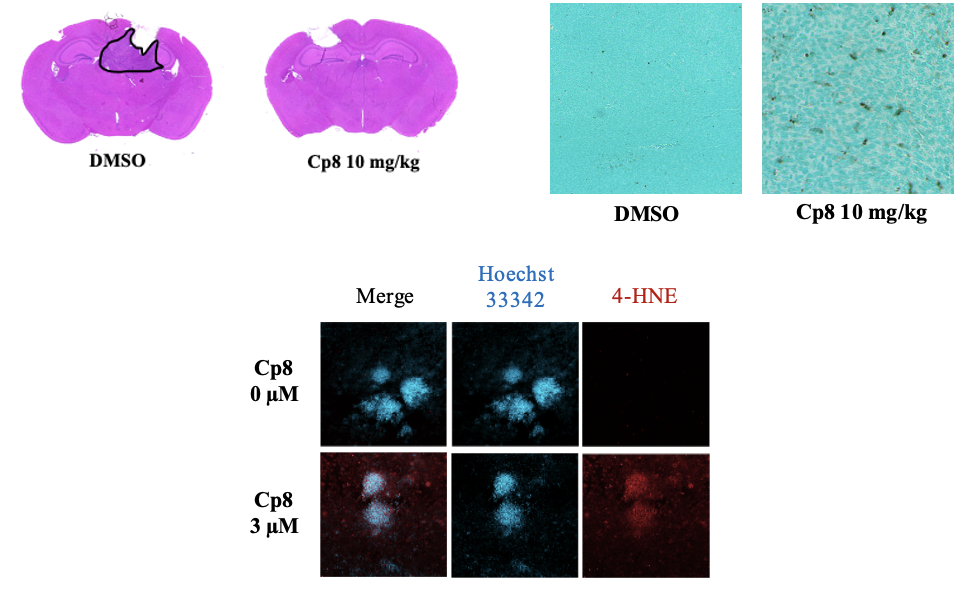


Representative TUNEL staining images from mice GBM brains of the treatment group (cp8 10 mg/kg) compared to the control group.

**Figure S11.**

**T98G**

sineg

GAPDH

SOX-2

siHDAC1

siHDAC2

sineg

siHDAC6

sineg


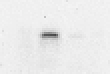

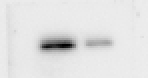


HDAC1/2/6


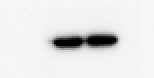

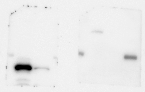

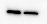

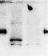

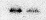

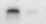

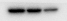


**T98G**

sineg

GAPDH

siHDAC1

sineg

siHDAC2

sineg

siHDAC6


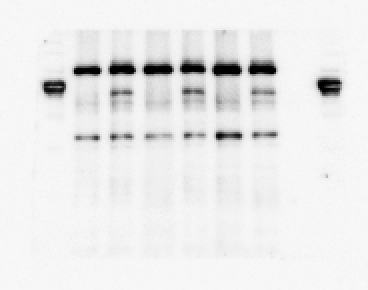


FLG


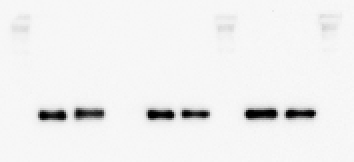

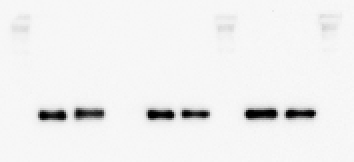

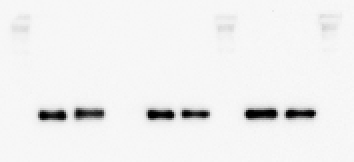


SOX-2 and FLG protein expression of HDAC1-, HDAC2-, or HDAC6-silencing T98G cells were examined using western blotting.

**Figure S12.**


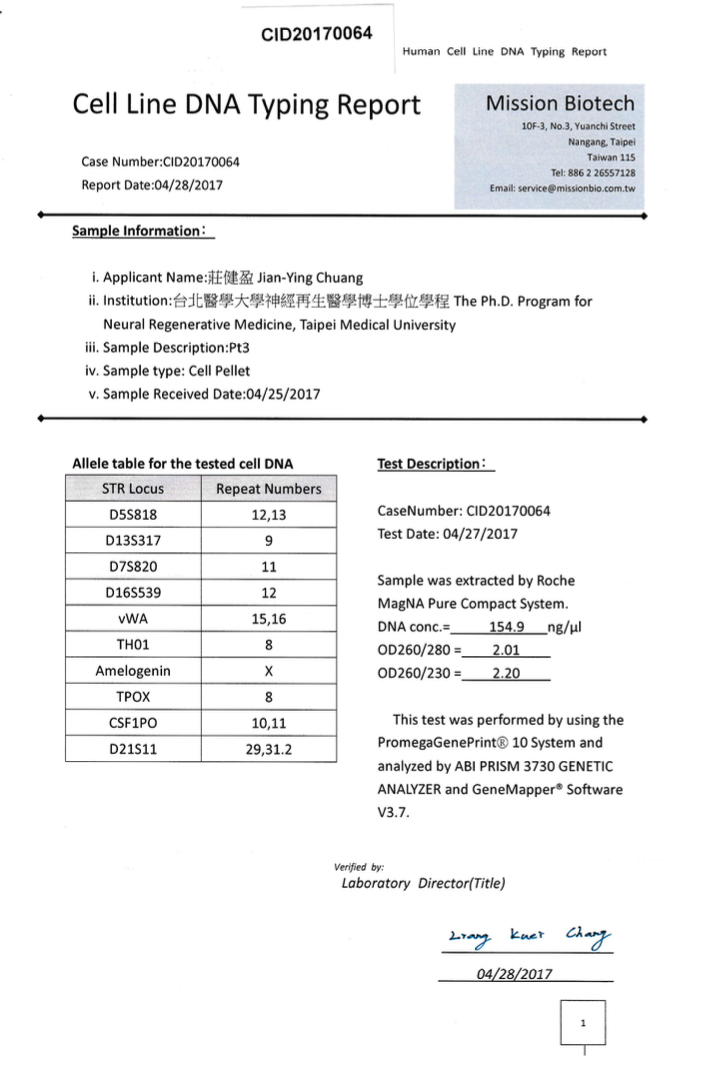


Cell line Pt#3 DNA typing report.

**Chemical synthesis scheme of abiraterone derivatives**

**Scheme S1.** Abiraterone (11) was treated with methane sulfonyl chloride in pyridine to produce a compound (12), which was then treated with sodium azide in DMF to produce an azide intermediate (13), which was then further reduced to an 3α amine (14), using triphenylphosphine as the reduction agent in a solvent mixture of methanol and THF. Intermediate (14) was subjected to reductive amination with 4-formylcinnamic ester to yield compound (15). Alternatively, compound (14) was reacted with 3-bromo benzene sulfonyl chloride which then underwent Heck olefination with tert-butyl acrylate yielding the corresponding cinnamate (15). The intermediates (15-16) were then prone to ester hydrolysis using LiOH, and the resulting carboxylic acid derivatives were then protected with NH_2_OTHP which then deprotected using TFA, resulting in the formation of corresponding hydroxamic acids (cp1 and cp2).

**The synthesis of compounds cp1 and cp2.**

Reagents and conditions: (a) CH_3_SO_2_Cl, pyridine, stirring, rt (b) NaN_3_, DMF, stirring, 80 °C (c) PPh_3_, MeOH, THF, stirring, 50 °C (d) For 15: (i) 4-formylcinnamic ester, NaCNBH3, CH_3_COOH, MeOH, rt; For 16: (i) 3-bromobenzenesulphonyl chloride, TEA, DCM, rt; (ii) Methylacrylate, Bis(tri-o- tolylphosphine)palladium, TEA, DMF, stirring, 100 °C b) LiOH(aq), dioxane, rt; (ii) NH_2_OTHP, EDC, HOBt, DIPEA, DMF, rt; (iii) 10% TFA, MeOH, rt.

**Scheme S2.** The amine derivative (14) was subjected to reductive amination with methyl-4-formyl benzoate using NaBH_3_CN to yield compound 17 and the reaction of 14 with Mono methylterphthalate in the presence of coupling reagents such as EDC, HOBt and DIPEA as base yielded the corresponding ester 18. The ester derivatives (17 and 18) were then converted to the designed hydroxamic acids (cp3 and cp4) following the same procedure used for (cp1 and cp2) as shown in Scheme 1. Moreover, intermediates 17 and 18 were coupled with o-phenylene diamine using EDC, HOBT, and DIPEA in DMF, resulting in final compounds (cp5 and cp6).

**The synthesis of compounds cp3 - cp6.**

Reagents and conditions: a) For 17: (i) Methyl-4-formylbenzoate, CH_3_COOH, NaCNBH_3_, MeOH, rt; For 18: Monomethylterphthalate, EDC.HCl, HOBt, DIPEA, DMF, rt; b) (i) LiOH(aq), dioxane, rt; (ii) NH_2_OTHP, EDC, HOBt, DIPEA, DMF, rt; iii) TFA(10% aq), MeOH, rt; c) o-phenylene diamines, EDC.HCl, HOBt, DIPEA, DMF, stirring, rt.

**Scheme S3.** Benzoic acid, diisopropyl azodicarboxylate (DIAD), and triphenylphosphine (TPP) were used to invert the 3β-alcohol and produce the benzoate ester (19), which was then hydrolysed with sodium methoxide in methanol to produce the 3α-alcohol (20), further treated with methane sulfonyl chloride in pyridine to produce a compound (21), which was then treated with sodium azide in DMF to produce an azide intermediate (22), which was then further reduced to an 3β amine (23), using triphenylphosphine as the reduction agent in a solvent mixture of methanol and THF. Further, reductive amination was performed for the synthesis of ester adducts by using amine (23) and Methyl 4-formylbenzoate. Ester intermediate was converted into the final compound (cp7) by utilizing the same methodology of compound (cp5).

**The synthesis of compound cp7.**

Reagents and conditions: (a) Benzoic acid, (C_6_H_5_)_3_P, DIAD, THF rt, 3h (b) Sodium methoxide solution in MeOH, DCM: MeOH, rt, overnight (c) CH_3_SO_2_Cl, pyridine, stirring, rt (d) NaN_3_, DMF, stirring, 80 °C (e) PPh_3_, CH_3_OH, THF, stirring, 50 °C (f) Methyl 4-formylbenzoate, NaBH_3_CN, CH_3_COOH, MeOH rt, 3h (g) i) 1 M LiOH(aq), p-dioxane, rt, 4h ii) NH_2_OTHP, EDC.HCl, HOBt, DIPEA, DMF, rt, 4h iii) 10% TFA (aq), MeOH, rt, 3h.

**Scheme S4.** Amine intermediate (23) was subjected to reductive amination with 4-formylcinnamic ester to yield ester intermediate (25). Alternatively, amine (23) was reacted with 4-bromo benzene sulfonyl chloride and 3-bromo benzene sulfonyl chloride which then underwent Heck olefination with tert-butyl acrylate yielding the corresponding cinnamate ester compounds (26 and 27). Ester adducts were converted into the desired hydroxamic acids (cp8 - cp10) using similar protocol utilised in Scheme 1.

**The synthesis of compounds cp8 - cp10.**

Reagents and conditions: (a) 25 4-formylcinnamic ester, NaCNBH3, acetic acid, MeOH, rt; for 26; i) 4-bromobenzenesulfonyl chloride, TEA, DCM rt, 3h, for 27 3-bromobenzenesulfonyl chloride, TEA, DCM rt, 3h, (ii) Methyl acrylate, K_2_CO_3_, TEA, Pd_2_(dba)_3_, [(t-Bu)_3_PH]BF_4,_ DMF, 100℃ (b) i)1 M LiOH(aq), p-dioxane, rt, 4h. ii) NH_2_OTHP, EDC.HCl, HOBt, DIPEA, DMF, rt, 4h iii) 10% TFA (aq), MeOH, rt, 3h.

**Experimental Section**

Nuclear magnetic resonance (^1^H NMR and ^13^C NMR) spectra were obtained with a Bruker DRX-500 spectrometer operating at 300 MHz & 600 MHz Chemical shifts are reported in parts per million (ppm, δ) downfield from TMS as an internal standard. High-resolution mass spectra (HRMS) were measured with a JEOL (JMS-700) electron impact (EI) mass spectrometer. Column chromatography was accomplished on silica gel (Merck Kieselgel 60, No.9385, 230e400 mesh ASTM). All reactions were carried out under an atmosphere of dry nitrogen.

*Synthesis of (3S,8R,9S,10R,13S,14S)-10,13-dimethyl-17-(pyridin-3-yl)-2,3,4,7,8,9,10,11,12,13,14,15-dodecahydro-1H-cyclopenta[a]phenanthren-3-yl methanesulfonate (12)*

A solution of compound (11) in pyridine was treated with methane sulfonyl chloride and stirred at room temperature for 3 hours. The reaction mixture was quenched with water and extracted with EtOAc (50 mL x 3). The combined organic layer was dried over anhydrous MgSO_4_, concentrated under reduced pressure, and was purified by silica gel chromatography (EtOAc: Hexane 2.5: 7.5) to give (12) in 84 % yield; ^1^H NMR (300 MHz, CDCl_3_): δ 1.11 - 1.12 (m, 6 H), 1.64-1.76 (m, 4 H), 1.79 – 1.98 (m, 6 H), 2.06 -2.19 (m, 4 H), 2.40 (m, 1 H), 2.55-2.58 (m, 2 H), 3.04 (s, 3 H), 4.56 (m, 1 H), 5.49 (d, *J* = 4.8 Hz, 1 H), 6.39 (s, 1 H), 7.88 (s, 1 H), 8.37 (d, *J* = 7.5 Hz, 1 H), 8.64 (s, 1 H), 8.75 (s, 1 H).

*Synthesis of 3-((3R,8R,9S,10R,13S,14S)-3-azido-10,13-dimethyl-2,3,4,7,8,9,10,11,12,13,14,15-dodecahydro-1H-cyclopenta[a]phenanthren-17-yl)pyridine (13).*

A solution of compound (12) (420 mg, 0.001 mol) in DMF (10 mL) was treated with sodium azide (195 mg, 0.003 mol carefully. The mixture was stirred and heated to 80 °C for 3 hours. The mixture was cooled to 25 °C and quenched with water and extracted with EtOAc (50 mL x 3). The combined organic layer was dried over anhydrous MgSO4, concentrated under reduced pressure and was purified by silica gel chromatography (EtOAc : Hexane : 2:8) to give (13) in 84 % yield; ^1^H NMR (300 MHz, DMSO): δ 1.03 - 1.05 (s, 6 H), 1.39-1.81 (m, 11 H), 2.02-2.14 (m, 4 H), 2.19-2.23 (m, 2 H), 4.01 (m, 1 H), 5.37 (d, *J* = 5.1 *Hz*, 1 H), 6.13 (m, 1 H), 7.35 (m, 1 H), 7.80 (m, 1 H), 8.44 (dd*, J*= 1.5 Hz & 4.7 Hz, 1 H), 8.60 (m, 1 H).

*Synthesis of (3R,8R,9S,10R,13S,14S)-10,13-dimethyl-17-(pyridin-3-yl)- 2,3,4,7,8,9,10,11,12,13,14,15-dodecahydro-1H-cyclopenta[a]phenanthren-3-amine (14).*

To a solution of (13) in methanol (5 mL) and THF (3 mL) was added PPh (296 mg 0.00113 mol). The mixture was stirred and heated to 50 °C overnight. The reaction mixture was cooled to 25 °C and the pH was adjusted to 1 with 2 N aqueous solution, extracted with DCM (50 mL). The pH of the aqueous solution was adjusted to 9 with sat. NaHCO and extracted with DCM (50 mL x 2). The organic layers were combined and dried over anhydrous MgSO4, concentrated under reduced pressure and was purified by silica gel chromatography (DCM : MeOH 93:7) to give (14) in 84 % yield; ^1^H NMR (300 MHz, DMSO-d*_6_*): δ 0.99 - 1.00 (s, 6 H), 1.11 (m, 1 H), 1.26 (m, 2 H), 1.34-1.40 (m, 2 H), 1.47-1.51 (m, 3 H), 1.53 - 1.68 (m, 4 H), 1.80 (m, 1 H), 1.95-2.09 (m, 4 H), 2.21 (m, 1 H), 3.05 (s, 1 H), 5.27 (d, *J*= 2.7 Hz, 1 H), 6.09 (m, 1 H), 7.32 (m, 1 H), 7.74 (m, 1 H), 8.41 (m, 1 H), 8.57 (s, 1 H).

*Synthesis of methyl (E)-3-(4-((((3R,10R,13S)-10,13-dimethyl-17-(pyridin-3-yl)-2,3,4, 7, 8, 9,10,11,12,13,14,15-dodecahydro-1H-cyclopenta[a]phenanthren-3-yl) amino) methyl)phenyl)acrylate* (15)

To the solution of compound (14), (100 mg, 0.28 mmol) in ethanol (6 mL), 4-formylcinnamic ester (54 mg, 0.28 mmol) and few drops of glacial acetic acid were added. The reaction mixture was stirred for 30 minutes at room temperature before adding sodium cyanoborohydride. The reaction was stirred for 3h at rt and then quenched with water. Ethyl acetate (3 x 50 mL) was used for extraction and the combined organic layer was dried over anhydrous MgSO4, concentrated under reduced pressure and purified by silica gel chromatography (EtOAc) to give compound (15) in 77% yield; ^1^H NMR (500 MHz, CDCl_3_): δ 1.02 (s, 3 H), 1.05 (s, 3 H), 1.29-1.30 (m, 2 H), 1.53 – 1.76 (m, 10 H), 2.00 – 2.13 (m, 5 H), 2.91 (s, 1 H), 3.77 (s, 5 H), 5.38 (d, *J* = 5 Hz, 1 H), 5.97(s, 1 H), 6.39 (d*, J* = 16 Hz, 1 H), 7.19 (m, 1 H), 7.35 (d*, J* = 8 Hz, 2 H), 7.45 (d*, J* = 8 Hz, 1 H), 7.61 – 7.66 (m, 2 H), 7.64 (d, *J* = 8 Hz, 1 H), 8.41 (d, *J*= 4.0 Hz, 1 H), 8.58 (s, 1 H).

*Synthesis of methyl (E)-3-(3-(N-((3R,10R,13S)-10,13-dimethyl-17-(pyridin-3-yl)-2,3,4,7,8,9,10,11,12,13,14,15-dodecahydro-1H-cyclopenta[a]phenanthren-3-yl)sulfamoyl)phenyl)acrylate* (16)

To the solution of compound (14), (280mg, 0.80mmol) in dichloromethane (4 mL), 3- bromobenzene sulfonyl chloride (225mg, 0.88mmol) was added at 0°C. The reaction mixture was stirred for 3 h at room temperature and then quenched with water. Ethyl acetate (3 x 50 mL) was used for extraction and the combined organic layer was dried over anhydrous MgSO4, concentrated under reduced pressure and purified by silica gel chromatography (EtOAc) to give a residue in 65% yield. To the solution of residue, Bis(tri-o-tolylphosphine) palladium (75mg,0.10mmol), triethyl amine (0.19ml,1.05mmol), and methyl acrylate (0.1 mL, 1.05mmol) in DMF was added and reaction mixture was stirred at 140 °C for 4 h. The reaction mixture was diluted with ethyl acetate and filtered over Celite. To the filtrate, water was added and extraction was done with ethyl acetate (3 x 50 mL). The combined organic layer was dried over anhydrous MgSO_4_, concentrated under reduced pressure to give a residue which was purified by silica gel chromatography (EtOAc: hexane: 2 : 3) to give compound (16) in 30 % yield; ^1^H NMR (300 MHz, DMSO): δ 0.96 (s, 3 H), 1.01 (s, 3 H), 1.35-1.44 (m, 5 H), 1.49-1.70 (m, 6 H), 1.81 (m, 1 H), 2.00-2.06 (m, 4 H), 2.22 (m, 1 H), 2.39 (s, 1 H), 3.74 (s, 3 H), 5.05 (s, 1 H), 6.15 (s, 1 H), 6.74 (d, *J* = 16 Hz, 1 H), 7.33 (m, 1 H), 7.38 (s, 1 H), 7.64 (t, *J* = 7.6 Hz, 2 H), 7.75 (d, *J* = 16 Hz, 1 H), 7.80 (s, 1 H), 7.86 (d, *J =* 7.2 Hz, 1 H), 7.97 (d, *J* = 7 Hz, 1 H), 8.21 (s, 1 H), 8.45 (s, 1 H), 8.62 (s, 1 H).

*Synthesis of (E)-3-(4-((((3R,10R,13S)-10,13-dimethyl-17-(pyridin-3-yl)-2,3,4,7,8,9,10,11,12,13,14,15-dodecahydro-1H-cyclopenta[a]phenanthren-3-yl) amino) methyl) phenyl)-N-hydroxy acrylamide* (cp1)

To a solution of compound (15), (160mg) in dioxane (5ml), LiOH (2 ml, 1M, aq) was added and the reaction mixture was stirred at room temperature for 4 h. The reaction mixture was then acidified using HCl (3N) and white precipitates (150mg) were filtered. To the solution of precipitates (150 mg, 0.29mmol) in DMF (5ml), EDC.HCl (113mg, 0.59mmol), HOBt (59mg, 0.43mmol) and DIPEA (0.12ml,0.73mmol) was added and stirred at room temperature for 30 min before adding NH_2_OTHP (51mg, 0.43 mmol). After being stirred for a further 5 h, the reaction mixture was quenched with water and extracted with EtOAc (50 mL x 3). The combined organic layer was dried over anhydrous MgSO_4_, concentrated under reduced pressure, and purified by silica gel chromatography (DCM: Methanol :: 98:2) to give a solid residue. The residue was dissolved in CH_3_OH (6mL), and 10% trifluoroacetic acid _(aq)_ (1.6mL) was added at room temperature. On the completion of the reaction, the mixture was concentrated under reduced pressure to give white precipitates. The precipitates were purified by silica gel chromatography (DCM: Methanol: 98:5) to afford the title compound (cp1) in 28.3% yield; mp: 229-230 °C; HPLC purity: - 95.52%. ^1^H NMR (500 MHz, DMSO-d*_6_*): δ 1.00 (s, 3 H), 1.01 (s, 3 H), 1.12 (bs, 1 H), 1.36-1.43 (m, 2 H), 1.47-1.56 (m, 4 H), 1.61-1.70 (m, 5 H), 1.97-2.05 (m, 4 H), 2.20 (m, 1 H), 2.37 (m, 1 H), 2.75 (bs, 2 H), 3.64 (s, 2 H), 5.26 (s, 1 H), 6.10 (s, 1 H), 6.39 (d, *J* = 15.8 Hz, 1 H), 7.33-7.47 (m, 6 H), 7.74 (d*, J*= 7.8 Hz, 1 H), 8.42 (s, 1 H), 8.58 (s, 1 H), 8.98 (bs, 1 H), 10.68 (bs, 1 H). ).^13^C NMR (75 MHz, CD_3_OD): 12.98, 15.48, 18.08, 20.46, 24.77, 26.67, 30.20, 31.09, 31.31, 34.78, 34.87, 36.60, 36.78, 47.08, 50.24, 57.46, 57.98, 118.54, 123.21, 123.94, 128.07, 130.06, 130.18, 135.63, 136.08, 138.41, 138.96, 145.57, 145.83, 150.94. HRMS (ESI) for C_34_H_42_N_3_O_2_ (M + H+): calcd, 524.3277; found, 524.3273.

*Synthesis of (E)-3-(3-(N-((3R,10R,13S)-10,13-dimethyl-17-(pyridin-3-yl)-2,3,4,7,8,9,10,11,12,13,14,15-dodecahydro-1H-cyclopenta[a]phenanthren-3-yl)sulfamoyl)phenyl)-N-hydroxyacrylamide* (cp2)

The title compound (cp2) was synthesized using compound (16) in a manner similar to that described for compound (cp1) in 38% yield; mp: 159-160 °C; HPLC purity: - 98.72%. ^1^H NMR (300 MHz, DMSO): δ 0.96 (s, 3 H), 1.02 (s, 3 H), 1.37-1.45 (m, 4 H), 1.54-1.71 (m, 7 H), 1.83 (m, 1 H), 1.98-2.06 (m, 4 H), 2.25 (m, 1 H), 2.38 (m, 1 H), 5.11 (d, *J* = 3.3 Hz, 1 H), 6.14 (s, 1 H), 6.57 (d, *J* = 15.8 Hz, 1 H), 7.33-7.40 (m, 2 H), 7.52 (d*, J* = 16.3 Hz, 1 H), 7.63 (t, *J* = 7.7 Hz, 1 H), 7.77-7.85 (m, 3 H), 8.01 (s, 1 H), 8.45 (d, *J*  = 3.6 Hz, 1 H), 8.61 (s, 1 H), 9.14 (s, 1 H), 10.85 (s, 1 H). ). ^13^C NMR (75 MHz, CD_3_OD): 15.34, 18.12, 20.38, 22.78, 29.38, 30.17, 30.96, 31.51, 34.60, 36.34, 37.72, 39.62, 47.12, 47.14, 50.29, 53.96, 57.46, 119.43, 121.01, 125.28, 125.71, 127.23, 129.52, 130.84, 133.21, 136.01, 138.32, 140.23, 140.77, 141.38, 141.69, 143.06, 149.30, 164.13. HRMS (ESI) for C_33_H_40_N_3_O_4_S (M + H+): calcd, 574.2740; found, 574.2742.

*Synthesis of methyl 4-((((3R,10R,13S)-10,13-dimethyl-17-(pyridin-3-yl)-2,3,4,7,8,9,10,11,12,13,14,15-dodecahydro-1H-cyclopenta[a]phenanthren-3-yl)amino)methyl)benzoate* (17)

To the solution of compound (14), (180 mg, 0.51mmol) in ethanol (20 mL), methyl-4- formyl benzoate (97 mg, 0.59 mmol) and few drops of glacial acetic acid were added. The reaction mixture was stirred for 30 minutes at room temperature before adding sodium cyanoborohydride. The reaction was stirred for 3 h at rt and then quenched with water. Ethyl acetate ( 3 x 50 mL) was used for extraction and the combined organic layer was dried over anhydrous MgSO_4_, concentrated under reduced pressure and purified by silica gel chromatography (EtOAc) to give a semisolid residue (17) in 50 % yield; ^1^H NMR (300 MHz, DMSO-d*_6_*): δ 1.01 (s, 3 H), 1.05 (s, 3 H), 1.43-1.48 (m, 2 H), 1.52-1.59 (m, 3 H), 1.64 – 1.72 (m, 6 H), 2.00-2.05 (m, 4 H), 2.46 (m, 1 H), 2.88 (bs, 1 H), 3.80 (s, 2 H), 3.87 (s, 3 H), 5.37 (d, *J* = 4.7 Hz, 1 H), 5.96-5.97 (m, 1 H), 7.19 (dd, *J* = 4.7 Hz, & 7.8 Hz, 1 H), 7.41 (d*, J* = 7.9 Hz, 2 H), 7.62 (d, *J* = 7.9 Hz, 1 H), 7.94 (d, *J* = 8.1 Hz, 2 H), 8.42 (s, 1 H), 8.59 (s, 1 H).

*Synthesis of methyl 4-(((3R,10R,13S)-10,13-dimethyl-17-(pyridin-3-yl)-2,3,4,7,8,9,10,11,12,13,14,15-dodecahydro-1H-cyclopenta[a]phenanthren-3-yl)carbamoyl)benzoate* (18)

A mixture of compound (14), (300 mg, 0.86mmol), EDC.HCl (330mg, 1.72 mmol), HOBt (174 mg, 1.2 mmol), DIPEA (0.39 ml, 3 mmol) in DMF (3 ml), and mono methyl terephthalate (232 mg, 1.3 mmol) was stirred at room temperature for 5h. The reaction mixture was quenched with water and extracted with EtOAc (50 mL x 3). The combined organic layer was dried over anhydrous MgSO4, concentrated under reduced pressure to give compound (18) in 56 % yield; ^1^H NMR (300 MHz, CD_3_OD): δ 1.12 (s, 3 H), 1.19 (s, 3 H), 1.25-1.32 (m, 2 H), 1.43-1.57 (m, 2 H), 1.62-1.86 (m, 6 H), 1.88 - 1.96 (m, 2 H), 2.08-2.18 (m, 3 H), 2.26-2.37 (m, 2 H), 2.72 (m, 1 H), 3.95 (s, 3 H), 4.25 (bs, 1 H), 5.50 (d, *J* = 5.1, 1 H), 6.11 (m, 1 H), 7.40 (dd*, J* = 8.1 Hz, & 5.1 Hz, 1 H), 7.81-7.88 (m, 3 H), 8.09 - 8.12 (m, 2 H), 8.41 (dd*, J* = 4.8 Hz & 1.5 Hz, 1 H), 8.55 (d, *J* = 1.5 Hz, 1 H).

*Synthesis of 4-((((3R,10R,13S)-10,13-dimethyl-17-(pyridin-3-yl)-2,3,4,7,8,9,10,11,12,13,14,15-dodecahydro-1H-cyclopenta[a]phenanthren-3-yl)amino)methyl)-N-hydroxybenzamide* (cp3)

To a solution of compound (17), (130 mg) in dioxane (3ml), LiOH (1.2 ml, 1M, aq) was added, and the reaction mixture was stirred at room temperature for 4 h. The reaction mixture was then acidified using HCl (3N), and white precipitates (90 mg) were filtered. To the solution of precipitates (90 mg, 0.18mmol) in DMF (4 ml), EDC.HCl (71mg, 0.37 mmol), HOBt (37 mg, 0.27 mmol), and DIPEA (0.08 ml,0.46 mmol) were added and stirred at room temperature for 30 min before adding NH_2_OTHP (32 mg, 0.27 mmol). After being stirred for a further 5 h, the reaction mixture was quenched with water and extracted with EtOAc (50 mL x 3). The combined organic layer was dried over anhydrous MgSO_4_, concentrated under reduced pressure and was purified by silica gel chromatography (EtOAc: Hexane: 2:2) to give solid residue. The residue was dissolved in CH_3_OH (4 mL), and Trifluoroacetic acid (1 mL) was added at 0 °C. The reaction mixture was stirred at room temperature for 3 h, and 20 ml of sodium bicarbonate solution (10 %) was added to the reaction mixture. Ethyl acetate (3 x 20 mL) was used for the extraction. The combined organic layer was dried over anhydrous MgSO_4_, concentrated under reduced pressure to give compound (cp3) in 32 % yield; mp: 125- 126 °C; HPLC purity: - 96.85%. ^1^H NMR (300 MHz, CD_3_OD): δ 1.08 (s, 3 H), 1.12 (s, 3H), 1.48 (m, 1 H), 1.58-1.83 (m, 9 H), 2.04-2.15 (m, 4 H), 2.18 (m, 1 H), 2.34 (m, 1 H), 2.49 (m, 1 H), 2.93 (bs, 1 H), 3.82 (s, 3 H), 5.44 (d, *J* = 4.4 Hz, 1 H), 6.08 (d*, J* = 1.4 Hz, 1 H), 7.37 (dd, *J* = 4.8 Hz, & 7.9 Hz, 1 H), 7.45 (d, *J* = 4.4 Hz, 1 H), 7.72 ( d, *J* = 8 Hz, 2 H), 7.83 (d, *J* = 7.8 Hz, 1 H), 8.38 (d, *J* = 4.3 Hz, 1 H), 8.53 (s, 1 H). ^13^C NMR (75 MHz, CDCl_3_): 15.57, 18.11, 20.33, 24.78, 26.71, 28.94, 3.31, 31.25, 31.33, 33.02, 35.05, 35.71, 37.21, 49.33, 50.44, 52.20, 57.66, 122.81, 123.55, 126.96, 128.53, 129.57, 133.62, 134.62, 138.95, 146.57, 146.73, 151.41. HRMS (ESI) for C_32_H_40_N_3_O_2_ (M + H+): calcd, 498.3121; found, 498.3120.

*Synthesis of N1-((3R,10R,13S)-10,13-dimethyl-17-(pyridin-3-yl)-2,3,4,7,8,9,10,11,12,13,14,15-dodecahydro-1H-cyclopenta[a]phenanthren-3-yl)-N4-hydroxyterephthalamide* (cp4)

The title compound (cp4) was synthesized using compound (18) in a manner similar to that described for compound (cp3) in 33.5 % yield; mp: 154-155 °C, HPLC purity: - 95.98%, ^1^H NMR (300 MHz, DMSO-d*_6_*): δ 1.05 (s, 3 H), 1.09 (s, 3 H), 1.42-1.46 (m, 2 H), 1.50-1.60(m, 4 H), 1.65-1.75 (m, 5 H), 2.00-2.11 (m, 4 H), 2.19-2.23 (m, 2 H), 4.14 (bs, 1 H), 5.34 (d, *J* = 4.7 Hz, 1 H), 6.15 (bs, 1 H), 7.36 (dd, *J* = 7.4 Hz, & 4.68 Hz, 1 H), 7.62 (d, *J* = 6.6 Hz, 1 H), 7.81 (s, 5 H), 8.46 (s, 1 H), 8.62 (s, 1 H), 9.12 (s, 1 H), 11.33 (s, 1 H). ^13^C NMR (75 MHz, CDCl_3_): 16.49, 18.75, 20.49, 25.91, 26.69, 30.27, 31.72, 34.16, 35.02, 36.98, 37.50, 46.45, 47.16, 50.59, 57.27, 123.45, 126.94, 127.39, 129.74, 133.33, 134.09, 134.38, 137.33, 139.10, 146.95, 151.10, 165.00, 166.25. HRMS (ESI) for C_32_H_38_N_3_O_3_ (M + H+): calcd, 512.2913; found, 512.2916.

*Synthesis of N-(2-aminophenyl)-4-((((3R,10R,13S)-10,13-dimethyl-17-(pyridin-3-yl)-2,3,4,7,8,9,10,11,12,13,14,15-dodecahydro-1H-cyclopenta[a]phenanthren-3-yl)amino)methyl)benzamide* (cp5)

To a solution of compound (17), (410mg) in dioxane (6 ml), LiOH (4 ml, 1M, aq) was added, and the reaction mixture was stirred at room temperature for 4 h. The reaction mixture was then acidified using HCl (3N) and filtered white precipitates (180 mg). To the solution of precipitates (180 mg, 0.36mmol) in DMF (4 ml), EDC.HCl (138mg, 0.71 mmol), HOBt (73 mg, 0.54mmol), and DIPEA (0.17 ml,0.9 mmol) were added and stirred at room temperature for 30 min before adding o-Phenylenediamine (39 mg, 0.36mmol). After being stirred for a further 5 h, the reaction mixture was quenched with water and extracted with EtOAc (50 mL x 3). The combined organic layer was dried over anhydrous MgSO_4_, concentrated under reduced pressure, and purified by silica gel chromatography (methanol: DCM: 5:95) to give compound (cp5) in 44% yield. Solid compound; mp: 164-165 °C, HPLC purity: - 98.22%, ^1^H NMR (500 MHz, CD_3_OD): δ 1.06, (s, 3 H), 1.10 (s, 3 H), 1.34 (m, 1 H), 1.47 (m,1 H), 1.85-1.79 (m, 9 H), 2.02-2.12 (m, 3 H), 2.16 (m, 1 H), 2.27 (m, 1 H), 2.51 (m, 1 H), 2.88 (bs, 1 H), 3.78 (s, 2 H), 5.41 (d, *J=* 4.9 Hz, 1 H), 6.05-6.06 (m, 1 H), 6.74 (t, *J* = 7.6 Hz, 1 H), 6.84( d, *J* = 7.8 Hz, 1 H), 7.04 ( m, 1 H) 7.16 (d, *J*= 7.5 Hz, 1 H), 7.33 (dd, *J* = 4.96 Hz &7.9 Hz , 1 H), 7.46 (d, *J* = 8 Hz, 2 H), 7.80 (m, 1 H), 7.92 (d, *J* = 8 Hz, 2 H), 8.34 (dd*, J*= 1.5 Hz & 4.8 Hz, 1 H), 8.50 (d, *J* = 1.9 Hz, 1 H). ^13^C NMR (75 MHz, CD_3_OD): 15.64, 18.21, 20.37, 25.06, 30.33, 31.29, 31.37, 33.18, 35.06, 36.08, 37.24, 49.51, 50.54, 51.98, 57.64, 117.38, 118.29, 122.56, 123.56, 123.99, 126.23, 127.13, 127.67, 128.34, 129.58, 132.97, 133.57, 134.59, 139.30, 142.37, 143.84, 146.59, 146.76, 151.37, 167.18. HRMS (ESI) for C_38_H_45_N_4_O (M + H+): calcd, 573.3593; found, 573.3593.

*Synthesis of N1-(2-aminophenyl)-N4-((3R,10R,13S)-10,13-dimethyl-17-(pyridin-3-yl)-2,3,4,7,8,9,10,11,12,13,14,15-dodecahydro-1H-cyclopenta[a]phenanthren-3-yl)terephthalamide* (cp6)

The title compound (cp6) was synthesized using (18) and *o*-Phenylenediamine in a manner similar to that described for compound (cp5) in 39 % yield; mp: 129-130 °C, HPLC purity: - 98.33%, ^1^H NMR (300 MHz, DMSO-*_d6)_*: δ 1.05 (s, 3 H), 1.10 (s, 3 H), 1.39-1.46 (m, 2 H), 1.47-1.61 (m, 4 H), 1.57-1.73 (m, 4 H), 2.00-2.11 (m, 5 H), 2.21-2.26 (m, 2 H), 4.17 (bs, 1 H), 4.92 (s, 2 H), 5.35 (s, 1 H), 6.14 (s, 1 H), 6.61 (t*, J* = 8.1 Hz, 1 H), 6.80 (d, *J*= 8.1 Hz, 1 H), 6.99 (t*, J* = 6.9 Hz, 1 H), 7.18 (d, *J* = 7.8 Hz, 1 H), 7.35 (dd*, J* = 4.5 Hz & 8.1 Hz, 1 H), 7.64 (d*, J* = 6 Hz, 1 H), 7.78 (d, *J* = 8.4 Hz, 1 H), 7.87 (d, *J* = 8.4 Hz, 2 H), 8.04 (d*, J*= 8 Hz, 2 H), 8.44 (dd, *J* = 1.2 Hz, & 4.6 Hz, 1 H), 8.61 (d*, J* = 1.6 Hz, 1 H), 9.75 (s, 1 H). ^13^C NMR (75 MHz, CD_3_OD): 15.74, 18.15, 20.40, 25.49, 30.26, 30.34, 31.32, 31.41, 33.77, 35.05, 35.62, 36.21, 37.25, 50.49, 57.56, 117.39, 118.20, 122.84, 123.60, 123.74, 126.21, 127.19, 127.67, 129.58, 133.47, 136.79, 137.74, 137.77, 139.01, 142.40, 146.62, 151.29, 166.30, 167.51, 167.58. HRMS (ESI) for C_38_H_43_N_4_O_2_ (M + H+): calcd, 587.3386; found, 587.3386.

*Synthesis of (3S,8R,9S,10R,13S,14S)-10,13-dimethyl-17-(pyridin-3-yl)-2,3,4,7,8,9,10,11,12,13,14,15-dodecahydro-1H-cyclopenta[a]phenanthren-3-yl benzoate* (19)

A mixture of (11), (5g, 0.0143 mol), benzoic acid (1.75g, 0.0143 mol), triphenylphosphine (3.93g, 0.015 mol), diisopropylazodicarboxylate (3ml, 0.0149 mol) in THF was stirred at room temperature for 3 h. The reaction mixture was quenched with water and extracted with EtOAc (50 ml x 3). The combined organic layers were dried over anhydrous MgSO_4_ and concentrated under reduced pressure. The residue was purified by silica gel chromatography (n-Hexane: EtOAc = 92:8) to give a solid product (19) in 52.7% yield; ^1^H NMR (300 MHz, CD_3_OD): δ 1.13, (s, 3H), 1.20 (s, 3H), 1.28 -1.60 (m, 5H), 1.71 -1.99 (m, 7H), 2.09-2.43 (m, 5H), 4.60 (bs, 1H), 5.39 (s, 1H), 6.12 (s, 1H), 7.39-7.43 (m, 3H), 7.56 (m, 1H), 7.88 (m, 1H), 7.98-8.06 (m, 2H), 8.41 (dd*, J* = 4.8 Hz and 1.5 Hz, 1H), 8.56 (m, 1H).

*Synthesis of (3R,8R,9S,10R,13S,14S)-10,13-dimethyl-17-(pyridin-3-yl)-2,3,4,7,8,9,10,11,12,13,14,15-dodecahydro-1H-cyclopenta[a]phenanthren-3-ol* (20)

Compound of (19), (6g, 0.013 mol), was dissolved in DCM and methanol (50:50) and sodium methoxide 5.4 M (3ml, 0.055 mol) was added to the solution. The reaction mixture was stirred for overnight at room temperature and then quenched with water and extracted with EtOAc (50 ml x 3). The combined organic layers were dried over anhydrous MgSO_4_ and concentrated under reduced pressure. The residue was purified by silica gel chromatography (n-Hexane: EtOAc = 8:2) to give a solid product (20) in 57.6% yield; ^1^H NMR (300 MHz, CDCl_3_): δ 1.08 (s, 3H), 1.10 (s, 3H), 1.28 -1.53(m, 5H), 1.67-1.83 (m, 7H), 2.63 (m, 5H), 4.06 (bs, 1H), 5.48 (bs, 1H), 6.03 (dd*, J =* 3.3 and 1.8 Hz, 1H), 7.25 (m, 1H), 7.68 (m, 1H), 8.49 (dd*, J* = 4.8 and 1.5 Hz, 1H), 8.65 (d, *J* = 1.8 Hz, 1H).

*Synthesis of (3R,8R,9S,10R,13S,14S)-10,13-dimethyl-17-(pyridin-3-yl)-2,3,4,7,8,9,10,11,12,13,14,15-dodecahydro-1H-cyclopenta[a]phenanthren-3-yl methanesulfonate* (21)

To accomplish the synthesis of compound (21), the intermediate (20) (3 g, 0.008 mol) was dissolved in pyridine and methane sulfonyl chloride was added to the solution. The reaction mixture stirred for 3 hours at room temperature and then quenched with water and extracted with EtOAc (50 ml x 3). The combined organic layers were dried over anhydrous MgSO_4_ and concentrated under reduced pressure. The residue was purified by silica gel chromatography (n-Hexane: EtOAc = 7.5:2.5) to give a solid product (21) in 70.8% yield; ^1^H NMR (300 MHz, CDCl_3_): δ 1.08 (s, 3H), 1.11 (d*, J* = 3.6 Hz, 3H), 1.62-1.87 (m, 11 H), 2.01-2.48 (m, 6H), 3.03 (s, 3H), 5.04 (bs, 1H), 5.44 (s, 1H), 6.03 (bs, 1H), 7.25 (m, 1H), 7.68 (m, 1H), 8.49 (dd, *J* = 4.8 Hz and 1.8 Hz, 1H), 8.65 (s, 1H).

*Synthesis of 3-((3S,8R,9S,10R,13S,14S)-3-azido-10,13-dimethyl-2,3,4,7,8,9,10,11,12,13,14,15-dodecahydro-1H-cyclopenta[a]phenanthren-17-yl)pyridine* (22)

The mixture of (21), (2.6 g, 0.006 mol) and sodium azide (1.5 g, 0.023 mol) was dissolved in DMF. The reaction mixture was heated at 80ºC for 3 hours and then quenched with water and extracted with EtOAc (50 ml x 3). The combined organic layers were dried over anhydrous MgSO_4_ and concentrated under reduced pressure. The residue was purified by silica gel chromatography (n-Hexane: EtOAc = 7.5:2.5) to give a solid product (22) in 87.2% yield; ^1^H NMR (300 MHz, DMSO): δ 1.03-1.05 (m, 9H), 1.09-1.25 (m, 6H), 1.63-1.71 (m, 2H), 1.98-2.29 (m, 7H), 5.45 (s, 1H), 6.13 (s, 1H), 7.35(m, 1H), 7.78 (dd, *J =* 7.8 Hz and 1.6 Hz, 1H), 8.45 (dd*, J* = 4.5 Hz*, J* = 1.2 Hz, 1H), 8.60 (s, 1H).

*Synthesis of (3S,8R,9S,10R,13S,14S)-10,13-dimethyl-17-(pyridin-3-yl)-2,3,4,7,8,9,10,11,12,13,14,15-dodecahydro-1H-cyclopenta[a]phenanthren-3-amine* (23)

For synthesis of title compound (23), the intermediate (22) (1.9g, 0.005 mol), triphenylphosphine (2g, 0.007 mol), was dissolved in the methanol and THF in ratio 20 ml: 12 ml. The reaction mixture was heated at 50ºC for overnight, quenched with water and extracted with butanol (50 ml x 3). The combined organic layers were dried over anhydrous MgSO_4_ and concentrated under reduced pressure. The residue was purified by silica gel chromatography (DCM: Methanol = 95:5) to give a solid product (23) in 60.8% yield; ^1^H NMR (300 MHz, DMSO): δ 1.02-1.07 (m, 7H), 1.24-1.44 (m, 3H), 1.52-1.70 (m, 6H), 1.77-1.88 (m, 4H), 2.02-2.35 (m, 5H), 3.18 (s, 1H), 5.34 (s, 1H), 6.13 (s, 1H), 7.35 (dd, *J* = 7.8 Hz and 4.8 Hz, 1H), 7.77 (d*, J* = 7.8 Hz, 1H), 8.44 (bs, 1H), 8.60 (s, 1H).

*Synthesis of methyl 4-((((3S,8R,9S,10R,13S,14S)-10,13-dimethyl-17-(pyridin-3-yl)-2,3,4,7,8,9,10,11,12,13,14,15-dodecahydro-1H-cyclopenta[a]phenanthren-3-yl) amino) methyl) benzoate* (24)

A mixture of compound (23), (300 mg, 0.0008 mol), Methyl 4-formylbenzoate (211 mg, 0.0012 mol), NaCNBH_3_ (82 mg, 0.0013 mol)_,_ and acetic acid (2 drops), were dissolved in methanol, stirred at room temperature for 3 hours and then quenched with water and extracted with EtOAc (50 ml x 3). The combined organic layers were dried over anhydrous MgSO_4_ and concentrated under reduced pressure. The residue was purified by silica gel chromatography EtOAc to give a solid product (24) with a yield of 60.88%. ^1^H NMR (300 MHz, CDCl_3_): δ 1.01 (s, 3H), 1.10 (s, 3H), 1.29 (s, 2H), 1.65-1.92 (m, 13H), 2.05-2.31(m, 3H), 3.93-3.95 (m, 5H), 5.39 (s, 1H), 6.02 (s, 1H), 7.27 (m, 1H), 7.43 (d, J = 8.4 Hz, 2H), 7.67 (d, *J* = 8.1Hz, 1H), 8.08 (d, J = 8.1 Hz, 2H), 8.48 (d, *J* = 4.8 Hz, 1H), 8.64 (d, *J* = 2.4 Hz, 1H).

*Synthesis of 4-((((3S,8R,9S,10R,13S,14S)-10,13-dimethyl-17-(pyridin-3-yl)-2,3,4,7,8,9,10,11,12,13,14,15-dodecahydro-1H-cyclopenta[a]phenanthren-3-yl) amino) methyl)-N-hydroxy benzamide* (cp7)

The title compound (cp7) was synthesized using compound (24) in a manner similar to that described for compound (cp1) in 32.82% yield; mp: 159-160 °C; HPLC purity: - 99.03%, ^1^H NMR (300 MHz, DMSO): δ 1.03 (s, 6H), 1.25 (bs, 3H), 1.40-1.57 (m, 2H), 1.63-1.84 (m, 5H), 2.00-2.29 (m, 6H), 2.33-2.38 (m, 3H), 3.85 (s, 2H), 4.11 (m, 1H), 5.33 (d, *J* = 4.5 Hz, 1H), 6.13 (s, 1H), 7.33 (dd, *J* = 7.8 and = 4.5 Hz, 1H), 7.44 (d*, J* = 8.0 Hz, 2H), 7.71 (d, *J* = 8.1 Hz, 2H), 7.78 (m, 1H), 8.45 (dd, *J* = 4.5 and 1.5 Hz, 1H), 8.60 (d*, J* = 2.1 Hz, 1H).^13^C (150 MHz, DMSO): 16.69, 19.33, 20.80, 26.98, 29.00, 29.10, 29.24, 29.44, 30.03, 30.31, 31.35, 31.73, 34.99, 36.98, 40.52, 47.08, 50.11, 57.35, 57.44, 123.82, 127.43, 129.44, 130.08, 132.56, 133.75, 147.60, 148.26, 151.46, 158.04, 158.24, 164.20, 164.21. HRMS (ESI) for C_32_H_40_N_3_O_2_ (M + H^+^): calcd, 498.3121; found, 498.3120.

*Synthesis of methyl (E)-3-(4-((((3S,8R,9S,10R,13S,14S)-10,13-dimethyl-17-(pyridin-3-yl)-2,3,4,7,8,9,10,11,12,13,14,15-dodecahydro-1H-cyclopenta[a]phenanthren-3-yl) amino) methyl) phenyl) acrylate* *(25)*

A mixture of compound (23), (300 mg, 0.0008 mol), Methyl 3-(4-formylphenyl) acrylate (164 mg, 0.0008 mol), NaCNBH_3_ (82 mg, 0.001 mol), and acetic acid (2 drops) was dissolved in methanol (4 ml). The reaction mixture was stirred at room temperature for 3 hours and then quenched with water and extracted with ethyl acetate (50 ml*3). The combined organic layers were dried over anhydrous MgSO_4_ and concentrated under reduced pressure. The residue was purified by silica gel chromatography (Ethyl acetate: n-hexane = 40:60 ) to give a solid product (25) in 71.11% yield; ^1^H NMR (300 MHz, DMSO): δ 1.05 (d*, J* = 2.1 Hz, 6H), 1.25 (bs, 4H), 1.38-1.72 (m, 6H), 1.92-2.08 (m, 6H), 2.20-2.39 (m, 2H), 3.18 (d, *J =* 4.8 Hz, 1H), 3.75 (s, 3H), 4.18 (bs, 2H), 5.45 (d, *J* = 4.2 Hz, 1H), 6.14 (s, 1H), 6.71 (d, *J* = 15.9 Hz, 1H), 7.36 (dd, *J* = 8.1 and 4.8 Hz, 1H), 7.54 (d, *J =* 8.1 Hz, 1H), 7.67-7.83 (m, 4H), 8.45 (dd, *J =* 4.5 and 1.5 Hz, 1H), 8.61 (d, *J =* 1.5 Hz, 1H).

*Synthesis of methyl (E)-3-(4-(N-((3S,8R,9S,10R,13S,14S)-10,13-dimethyl-17-(pyridin-3-yl)-2,3,4,7,8,9,10,11,12,13,14,15-dodecahydro-1H-cyclopenta[a]phenanthren-3-yl) sulfamoyl) phenyl) acrylate (26)*

To a solution of (23), (300 mg, 0.0008 mol) in DCM (4 ml) was added 4-bromo benzene sulfonyl chloride (241 mg, 0.0009 mol) and TEA (0.12 ml 0.0008 mol). The reaction mixture was stirred at room temperature for 3hours, quenched with water and extracted with EtOAc (50 ml x 3). The combined organic layers were dried over anhydrous MgSO_4_ and concentrated under reduced pressure. The residue was purified by silica gel chromatography (n-Hexane: EtOAc = 92:8) to give a solid product in 63.65% yield. Tris (dibenzylidene acetone) dipalladium (74 mg, 0.00008 mol), methyl acrylate (0.11ml, 0.0012 mol), K_2_CO_3_ (112 mg, 0.0008 mol), TEA (110 mg, 0.0010 mol) and Tri-*tert*-butyl phosphonium tetrafluoroborate (47 mg, 0.0001 mol) was dissolved in DMF. The reaction mixture was refluxed for 1hour. The reaction mixture was filtered over Celite, and the filtrate was dried in vacuum and purified by silica gel chromatography (n-Hexane: EtOAc = 6.5:3.5) to give a solid product (26) in 60.7% yield. ^1^H NMR (300 MHz, CDCl_3_): 1.00 (s, 3H), 1.03 (s, 3H), 1.14-1.81 (m, 11H), 2.01-2.10 (m, 3H), 2.17-2.23 (m, 3H), 3.10 (m,1H), 3.83 (s, 3H), 5.29 (d, *J* = 4.5 Hz, 1H), 6.02 (dd, *J* = 3.3 and 1.8 Hz, 1H), 6.55 (d, *J* = 15.9 Hz, 1H), 7.27 (m, 1H), 7.55 (m, 1H), 7.69-7.71 (m, 2H), 7.91(m, 1H), 8.03 - 8.07 (m, 2H), 8.48 (d, *J* = 3.9 Hz, 1H), 8.63 (s, 1H).

*Synthesis of methyl (E)-3-(3-(N-((3S,8R,9S,10R,13S,14S)-10,13-dimethyl-17-(pyridin-3-yl)-2,3,4,7,8,9,10,11,12,13,14,15-dodecahydro-1H-cyclopenta[a]phenanthren-3-yl) sulfamoyl) phenyl) acrylate (27)*

The title compound (27) was obtained in 60.7% yield from (23) and 3-bromobenzenesulfonyl chloride in a manner similar to that described for the synthesis of compound (26); ^1^H NMR (300 MHz, CDCl_3_): δ 1.00 (s, 3H), 1.03 (s, 3H), 1.25-1.55(m, 4H), 1.57-1.63 (m, 3H), 1.68-1.71 (m, 2H), 1.74 – 1.81(m, 2H), 2.01-2.10 (m, 3H), 2.17-2.23 (m, 3H), 3.10 (m, 1H), 3.83 (s, 3H), 5.29 (d, *J* = 4.5 Hz, 1H), 6.02 (m, 1H), 6.55 (d, *J* = 15.9 Hz, 1H), 7.26 (m, 1H), 7.56 (m, 1H), 7.69-7.74 (m, 2H), 7.91 (m, 1H), 8.03-8.06 (m, 2H), 8.48 (d, *J* = 3.9 Hz, 1H), 8.63 (s, 1H).

*Synthesis of (E)-3-(4-((((3S,8R,9S,10R,13S,14S)-10,13-dimethyl-17-(pyridin-3-yl)-2,3,4,7,8,9,10,11,12,13,14,15-dodecahydro-1H-cyclopenta[a]phenanthren-3-yl) amino) methyl) phenyl)-N-hydroxyacrylamide* (cp8)

The title compound (cp8) was synthesized using compound (25) in a manner similar to that described for compound (cp1) in 40.4% yield; mp: 169-170 °C; HPLC purity: - 96.49%, ^1^H NMR (300 MHz, MeOD): δ 1.13 (s, 3H), 1.17 (s, 3H), 1.33 (bs, 2H), 1.61-1.96 (m, 7H), 2.05-2.22 (m, 9H), 4.60 (s, 2H), 5.59 (s, 1H), 6.12 (s, 1H), 6.55 (d*, J* = 15.9 Hz, 1H), 7.41 (m, 1H), 7.56 (t, *J* = 7.5 Hz, 3H), 7.67 (t, *J* = 8.1 Hz, 2H), 7.87 (d, *J* = 6.9 Hz, 1H), 8.42 (d, *J* = 4.5 Hz, 1H), 8.56 (s, 1H). ^13^C (150 MHz, CD_3_OD): 15.50, 18.06, 20.47, 24.76, 26.68, 30.21, 31.09, 31.30, 34.78, 34.89, 36.60, 36.79, 47.08, 50.25, 57.46, 57.98, 118.52, 122.18, 123.18, 123.83, 128.06, 129.43, 130.00, 130.07, 132.79, 133.80, 135.36, 136.05, 138.44, 138.98, 145.82, 146.07, 151.03, 164.45. HRMS (ESI) for C_34_H_42_N_3_O_2_ (M + H^+^): calcd, 524.3277; found, 524.3280.

*Synthesis of (E)-3-(4-(N-((3S,8R,9S,10R,13S,14S)-10,13-dimethyl-17-(pyridin-3-yl)-2,3,4,7,8,9,10,11,12,13,14,15-dodecahydro-1H-cyclopenta[a]phenanthren-3-yl) sulfamoyl) phenyl)-N-hydroxy acrylamide* (cp9)

The title compound (cp9) was obtained in 26.1% yield from compound (26) in a manner similar to that described for synthesis of compound (cp1); mp: 167-168 °C, HPLC purity: - 95.20%, ^1^H NMR (300 MHz, DMSO): 1.06 (s, 3H), 1.12 (s, 3H), 1.32 (bs, 2H), 1.48-1.55 (m, 2H), 1.63-1.85 (m, 7H), 2.02-2.40 (m, 6H), 2.98 (m, 1H), 5.27 (d, *J* = 5.1 Hz, 1H), 6.37 (dd, *J* = 3.0 and 1.8 Hz, 1H), 6.63 (d*, J* = 15.9 Hz, 1H), 7.64 (d*, J* = 15.9 Hz, 1H), 7.75-7.92 (m, 5H), 8.40 (dd, *J* = 8.1 and 1.8 Hz, 1H), 8.62 (d, *J* = 4.8 Hz, 1H), 8.76 (s, 1H). ^13^C (150 MHz, CD_3_OD): 15.31, 18.12, 20.38, 22.78, 29.38, 30.17, 30.96, 31.54, 34.59, 36.34, 37.72, 39.62, 47.12, 50.30, 53.96, 57.46, 119.43, 121.00, 125.28, 125.73, 127.23, 129.52, 130.84, 133.25, 136.01, 138.31, 140.29, 140.77, 141.32, 141.63, 143.07, 149.28, 164.12. HRMS (ESI) for C_33_H_40_N_3_O_4_S (M + H^+^): calcd, 574.2740; found, 574.2739.

*Synthesis of (E)-3-(3-(N-((3S,8R,9S,10R,13S,14S)-10,13-dimethyl-17-(pyridin-3-yl)-2,3,4,7,8,9,10,11,12,13,14,15-dodecahydro-1H-cyclopenta[a]phenanthren-3-yl) sulfamoyl) phenyl)-N-hydroxy acrylamide* (cp10)

The title compound (cp10) was obtained in 25.2% yield from compound (27) in a manner similar to that described for synthesis of compound (cp1); mp: 161-162 °C, HPLC purity: - 98.22%, ^1^H NMR (300 MHz, DMSO): 0.95 (s, 3H), 1.01 (s, 3H), 1.25 (bs, 2H), 1.42-1.75(m, 8H), 1.95-2.10 (m, 7H), 2.90 (m, 1H), 5.19 (d*, J* = 4.5 Hz, 1H), 6.25 (s, 1H), 6.58 (d, *J* = 15.9 Hz, 1H), 7.51-7.67 (m, 3H), 7.79 - 7.81 (m, 3H), 8.05 (dd, *J* = 5.1 and 1.5 Hz, 1H), 8.56 (dd*, J* = 5.1 and 1.2 Hz, 1H), 8.70 (d, *J* = 1.8 Hz, 1H). ^13^C (150 MHz, CD_3_OD): 15.35, 18.14, 20.40,28.12, 29.36,30.18, 30.99, 31.48, 34.65, 36.34, 37.71, 39.64, 47.10, 50.32, 53.92, 57.46, 120.21, 121.00,125.45, 127.02, 127.90, 129.43, 132.72, 135.46, 138.21, 138.74, 139.48, 140.79, 142.03, 142.35, 142.73, 149.56,164.08. HRMS (ESI) for C_33_H_40_N_3_O_4_S (M + H^+^): calcd, 574.2740; found, 574.2739.

**Chemical synthesis section**

**^1^H NMR of Compound (cp1)**


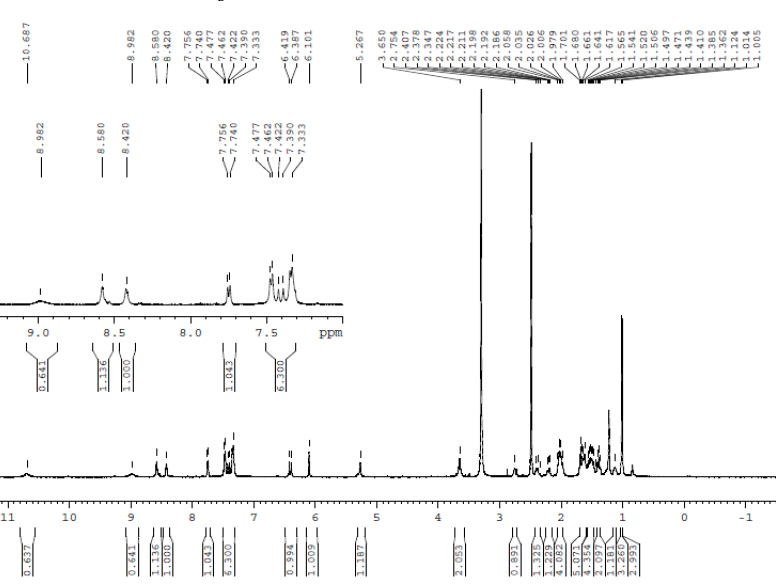


**^1^H NMR of Compound (cp2)**


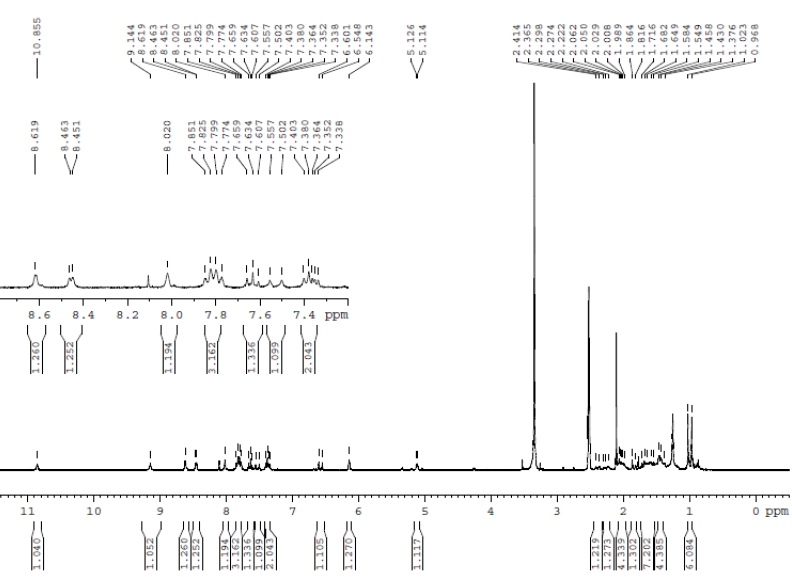


**^1^H NMR of Compound (cp3)**


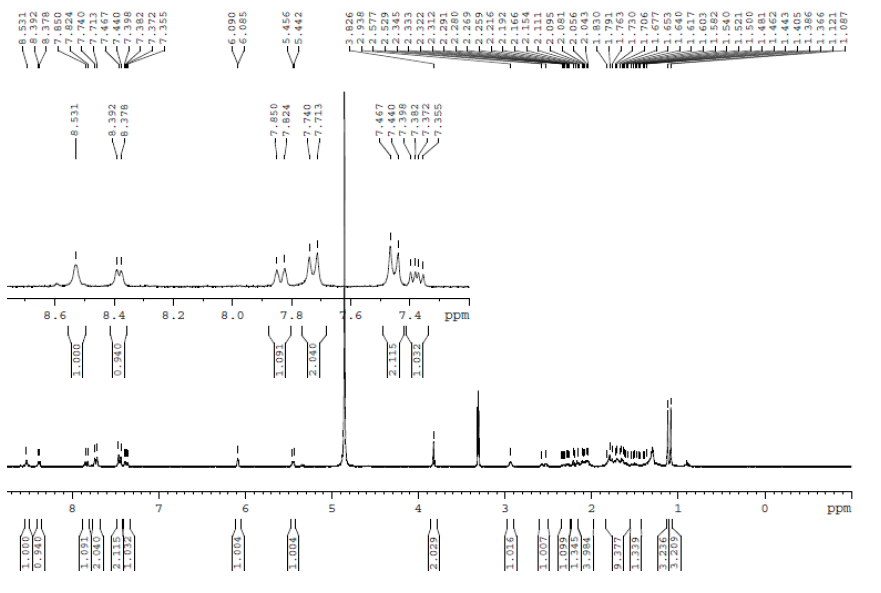


**^1^H NMR of Compound (cp4)**


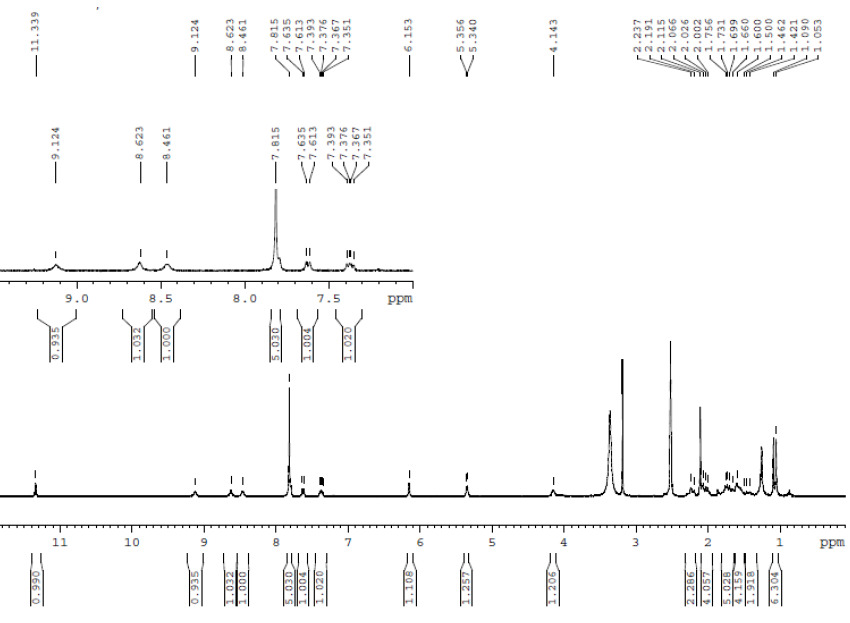


**^1^H NMR of Compound (cp5)**


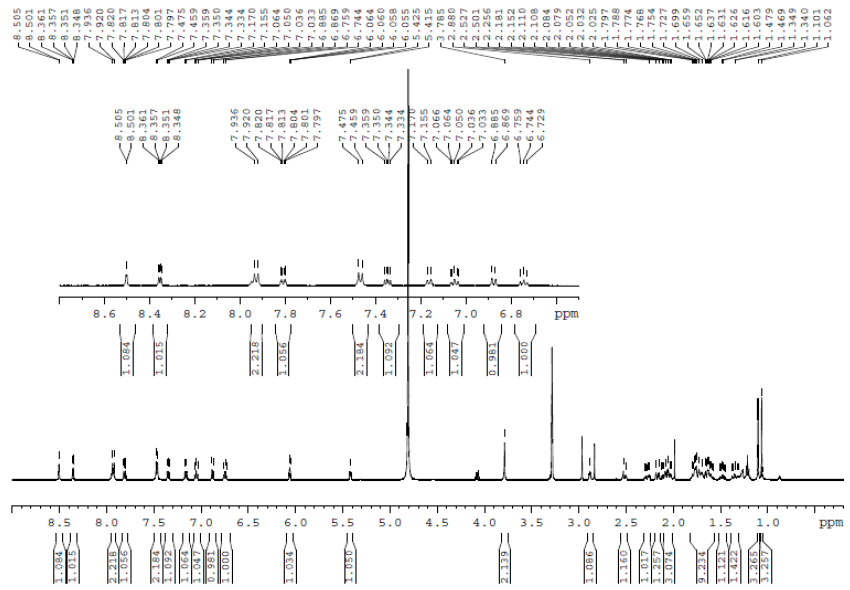


**^1^H NMR of Compound (cp6)**


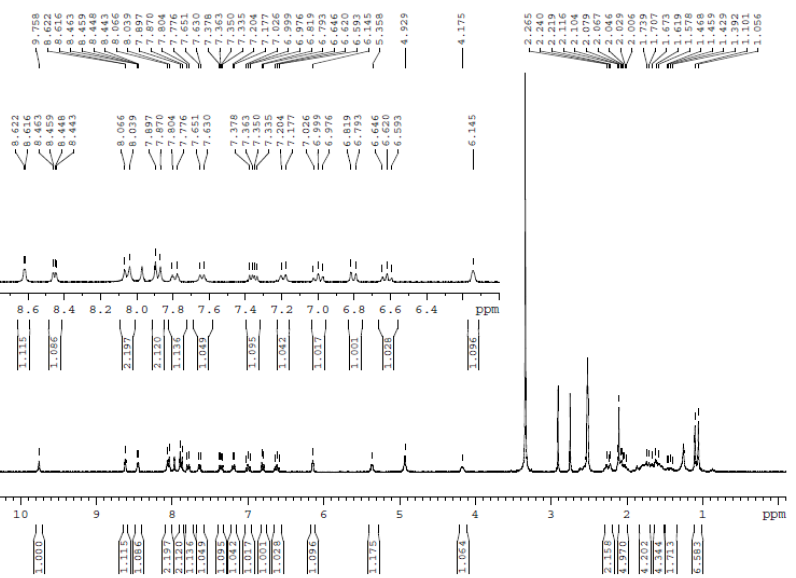


**^1^H NMR of Compound (cp7)**


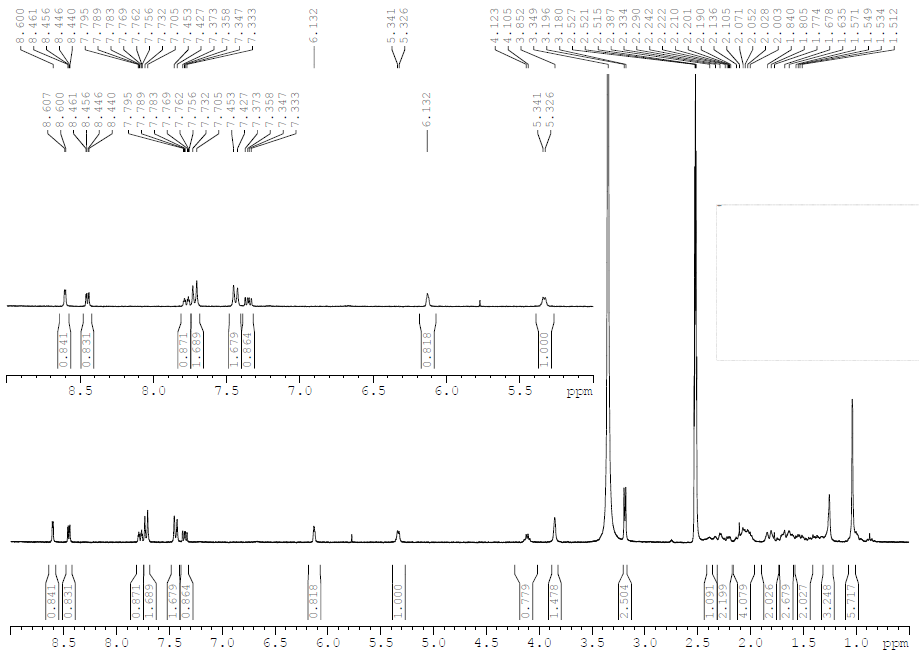


**^1^H NMR of Compound (cp8)**


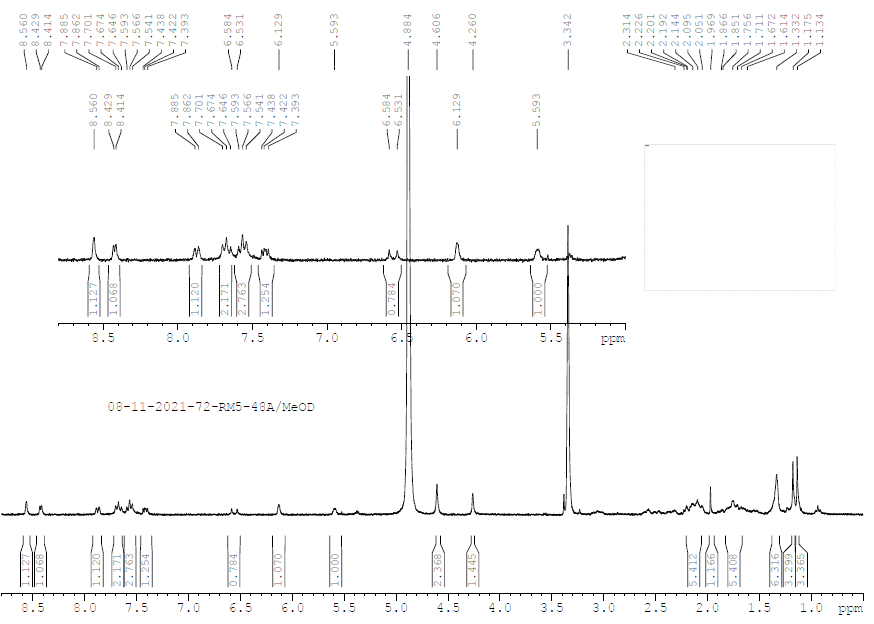


**^1^H NMR of Compound (cp9)**


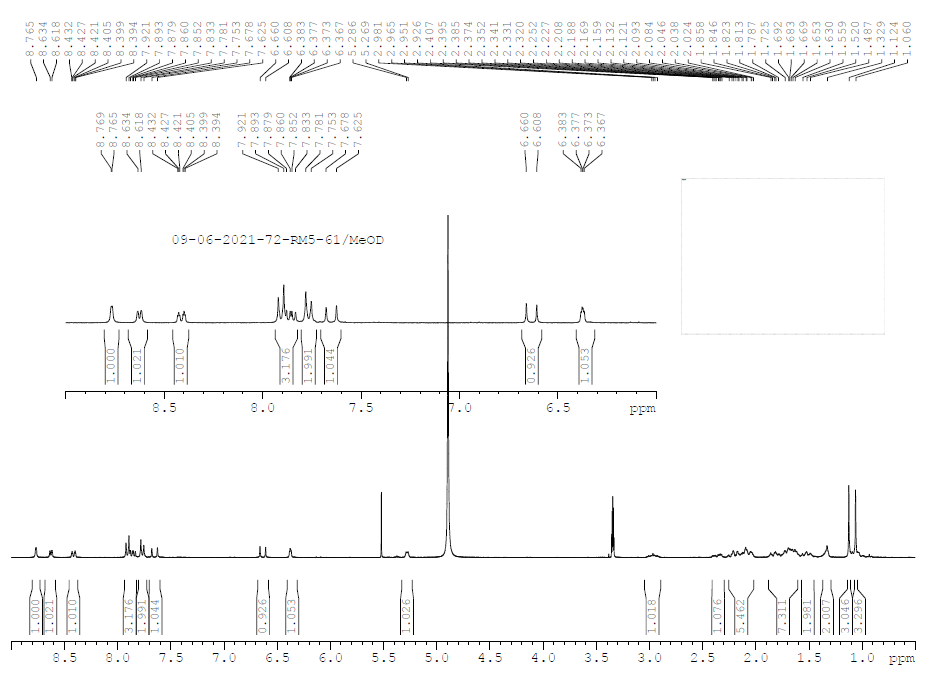


**^1^H NMR of Compound (cp10)**


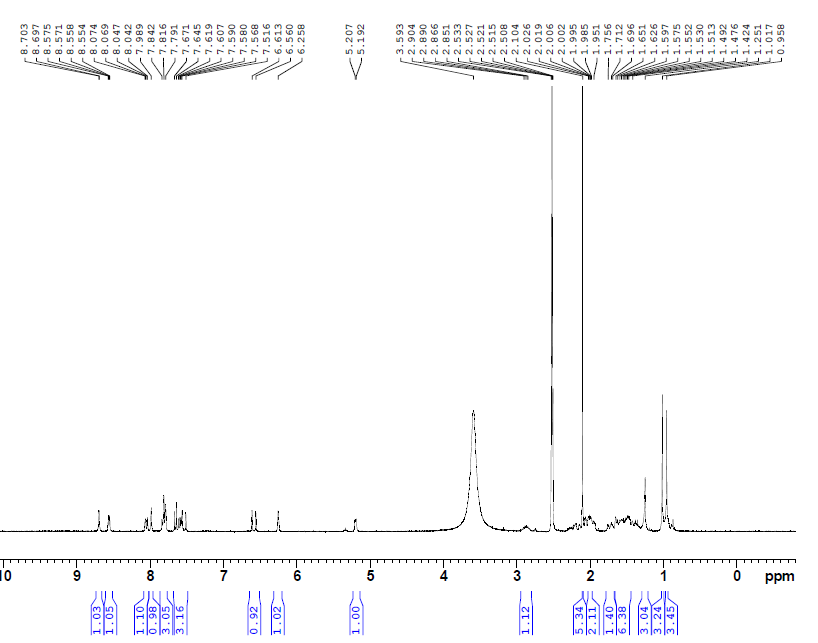


**^13^C NMR of Compound (cp1)**


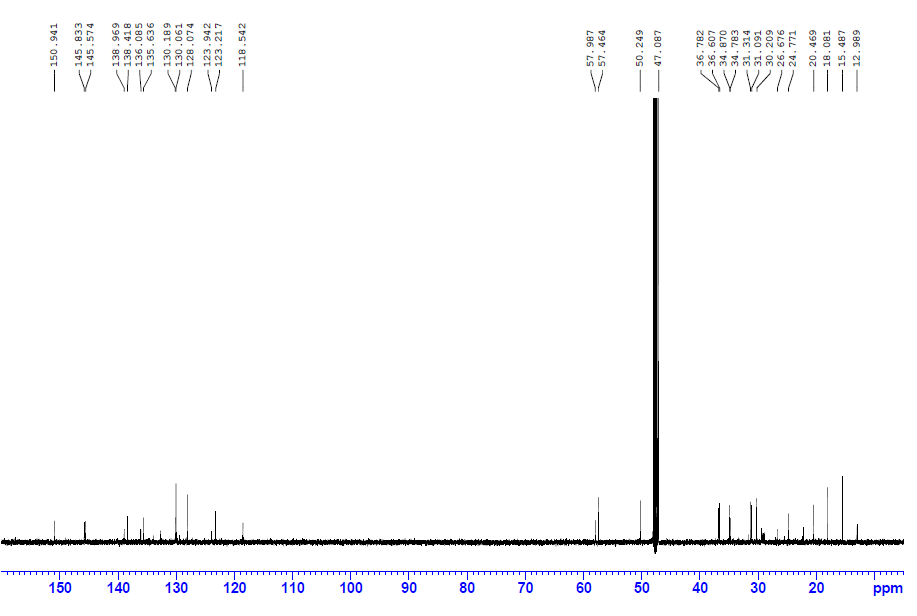


**^13^C NMR of Compound (cp2)**


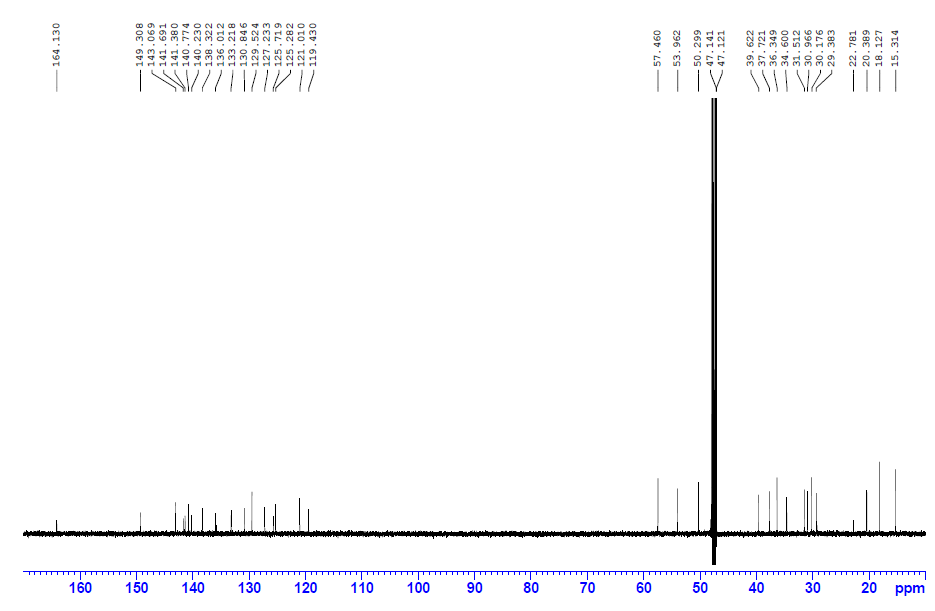


**^13^C NMR of Compound (cp3)**


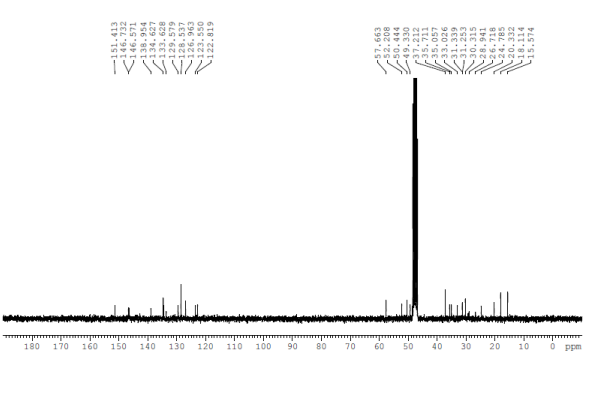


**^13^C NMR of Compound (cp4)**


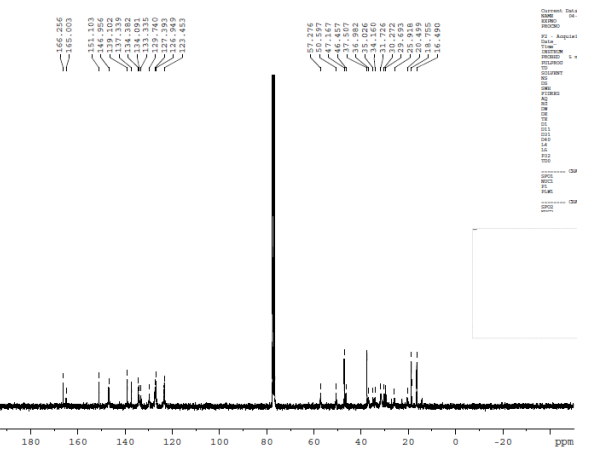


**^13^C NMR of Compound (cp5)**


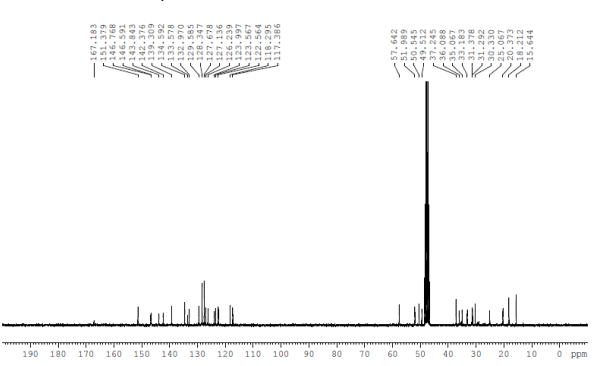


**^13^C NMR of Compound (cp6)**


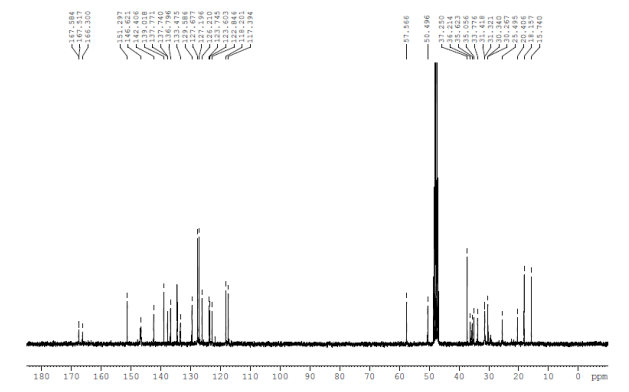


**^13^C NMR of Compound (cp7)**


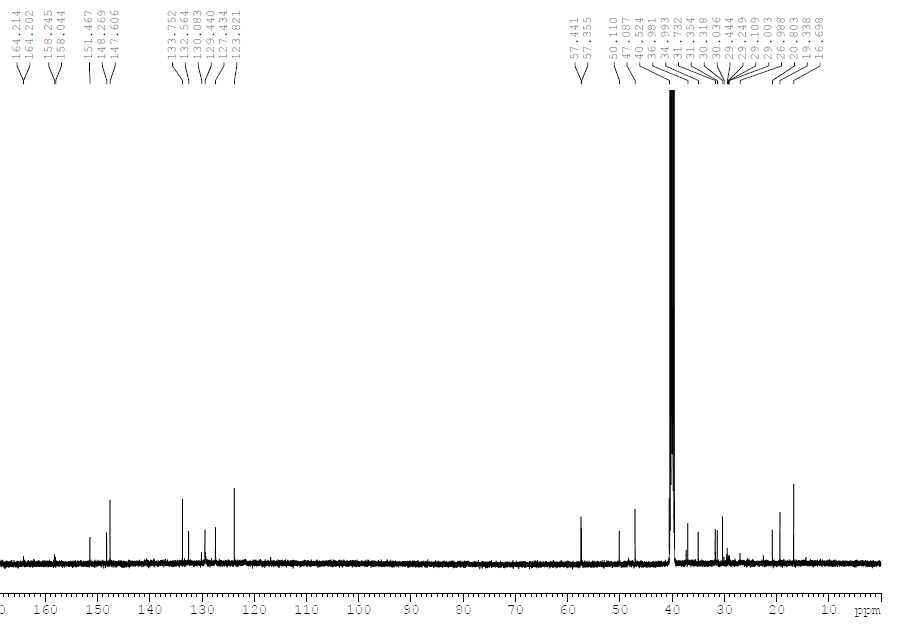


**^13^C NMR of Compound (cp8)**


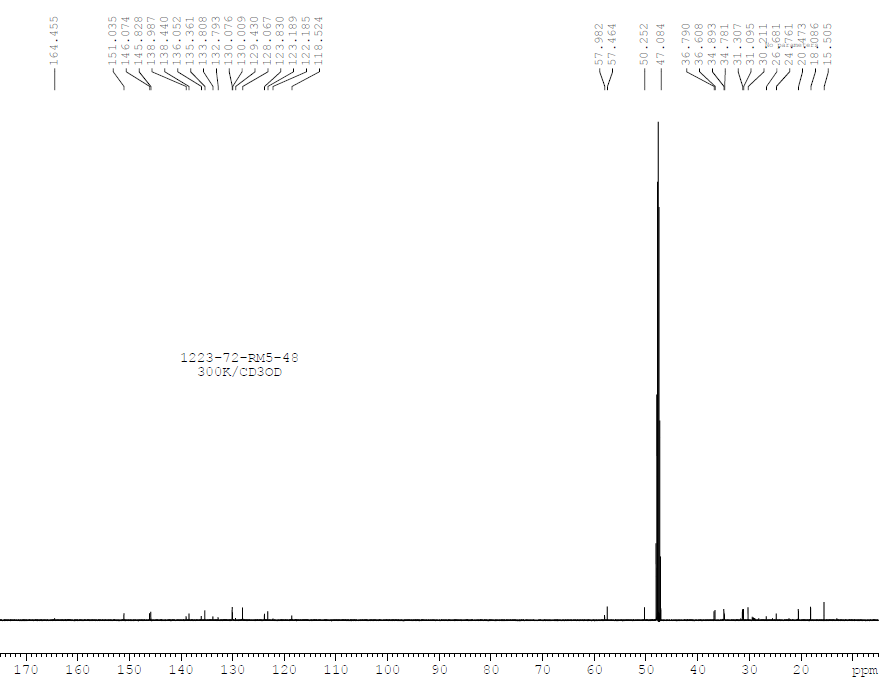


**^13^C NMR of Compound (cp9)**


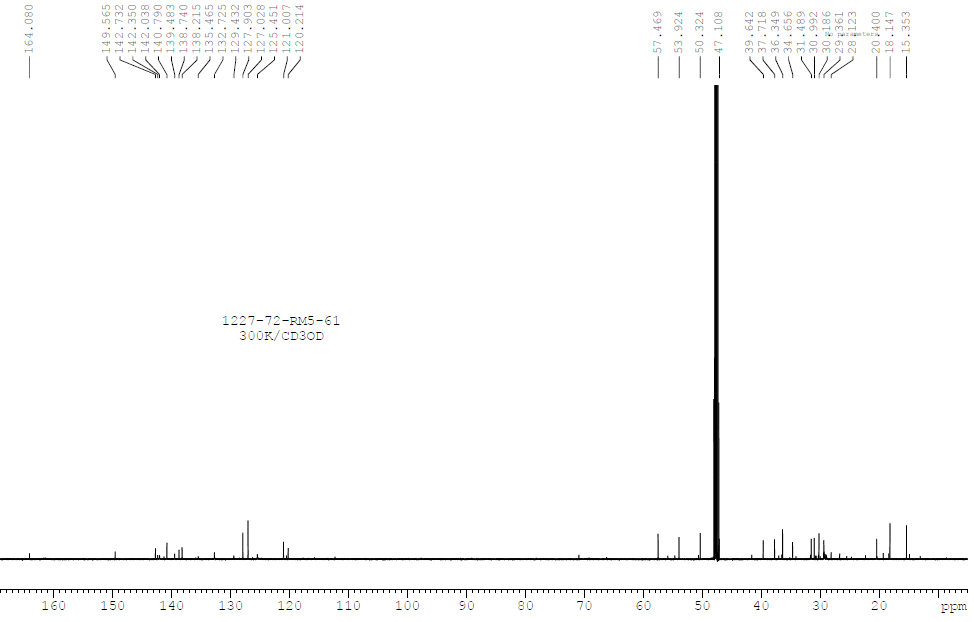


**^13^C NMR of Compound (cp10)**


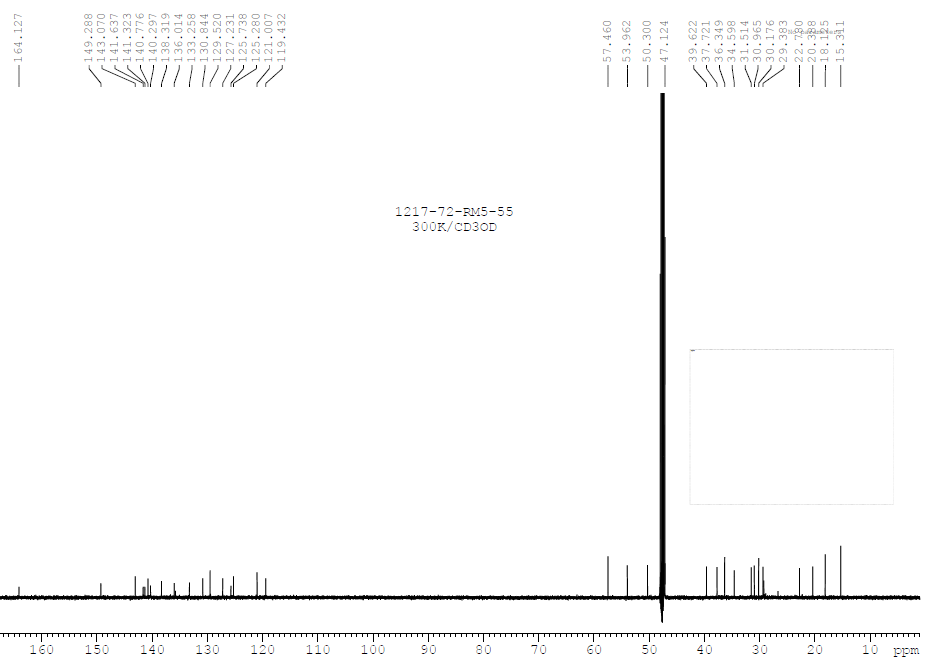


**HRMS Data of Compound (cp1)**


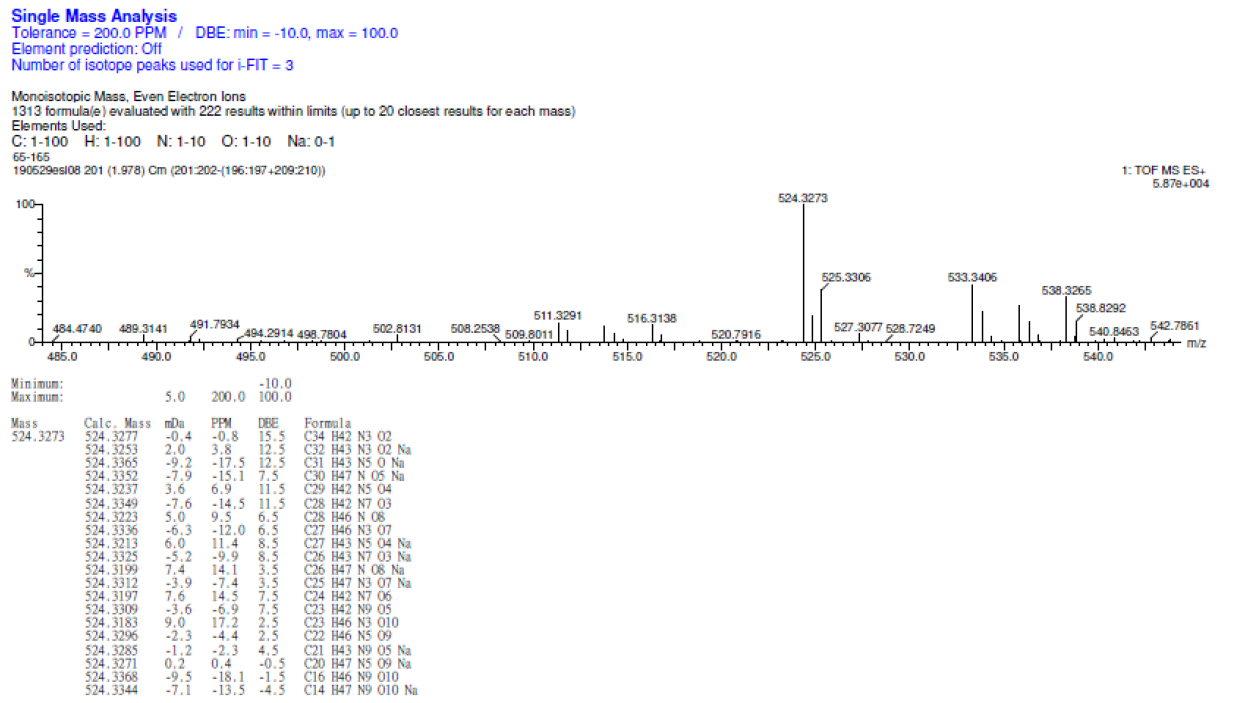


**HRMS Data of Compound (cp2)**


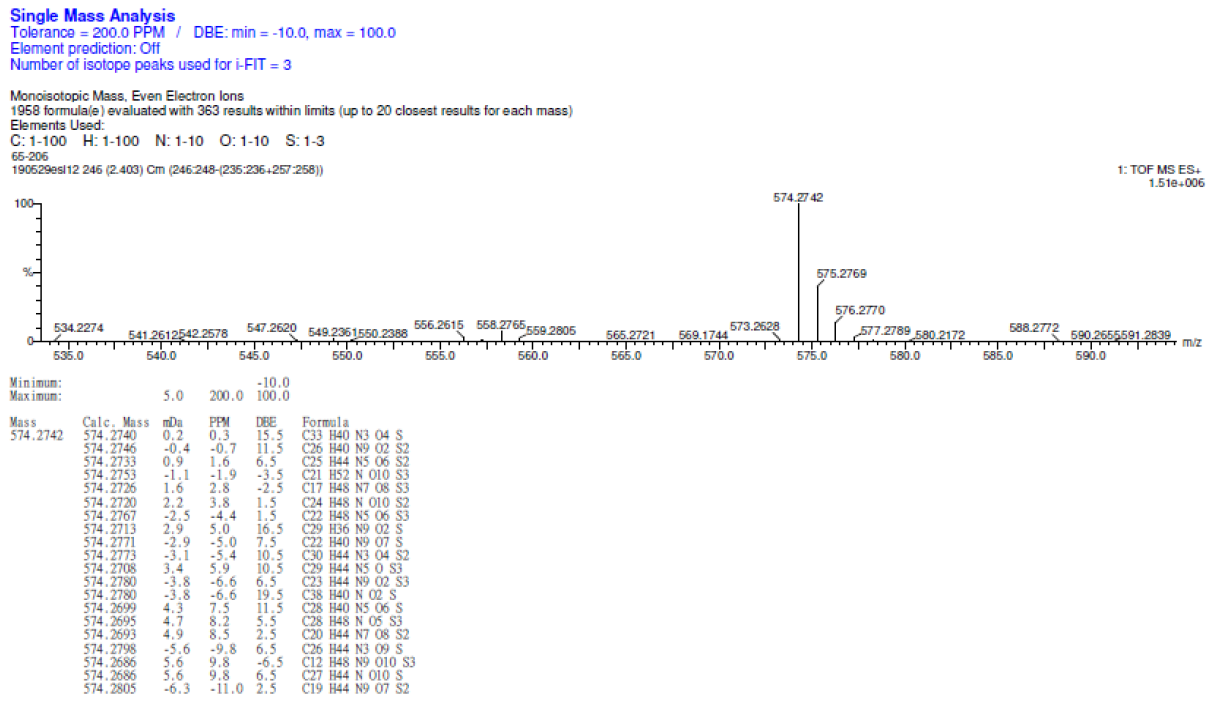


**HRMS Data of Compound (cp3)**


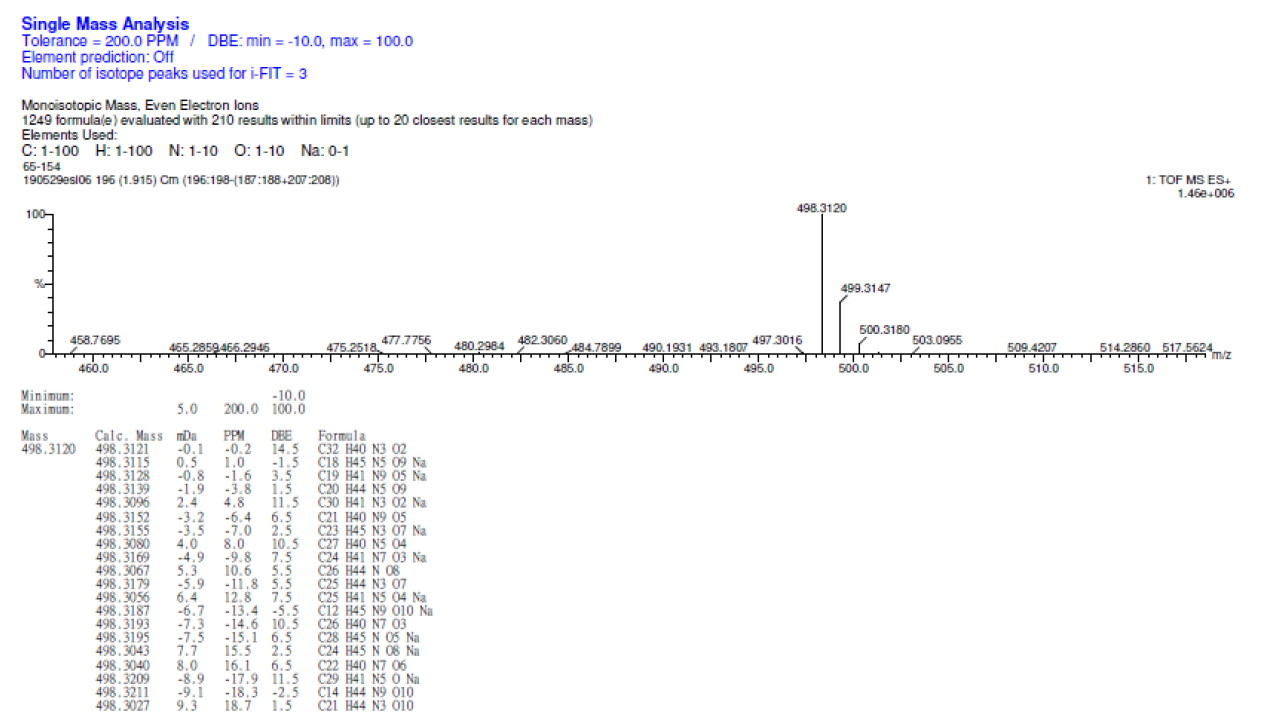


**HRMS Data of Compound (cp4)**


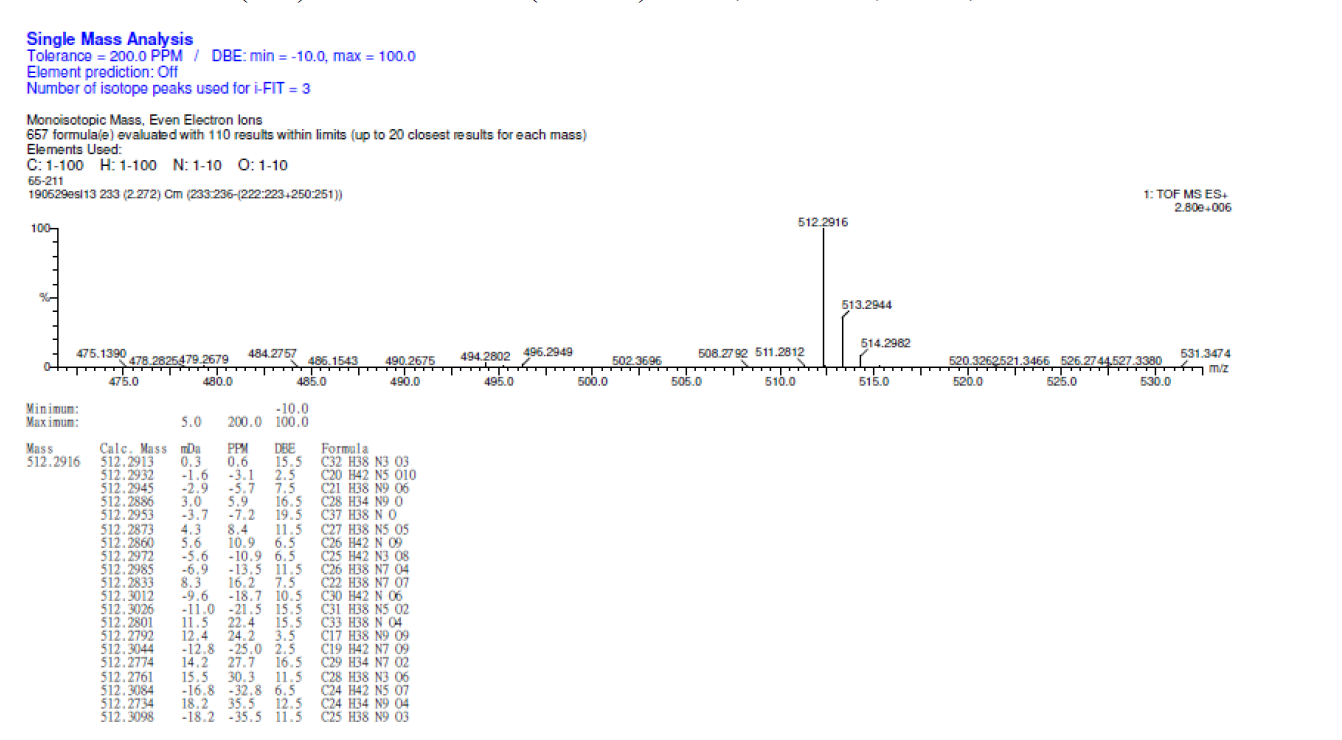


**HRMS Data of Compound (cp5)**


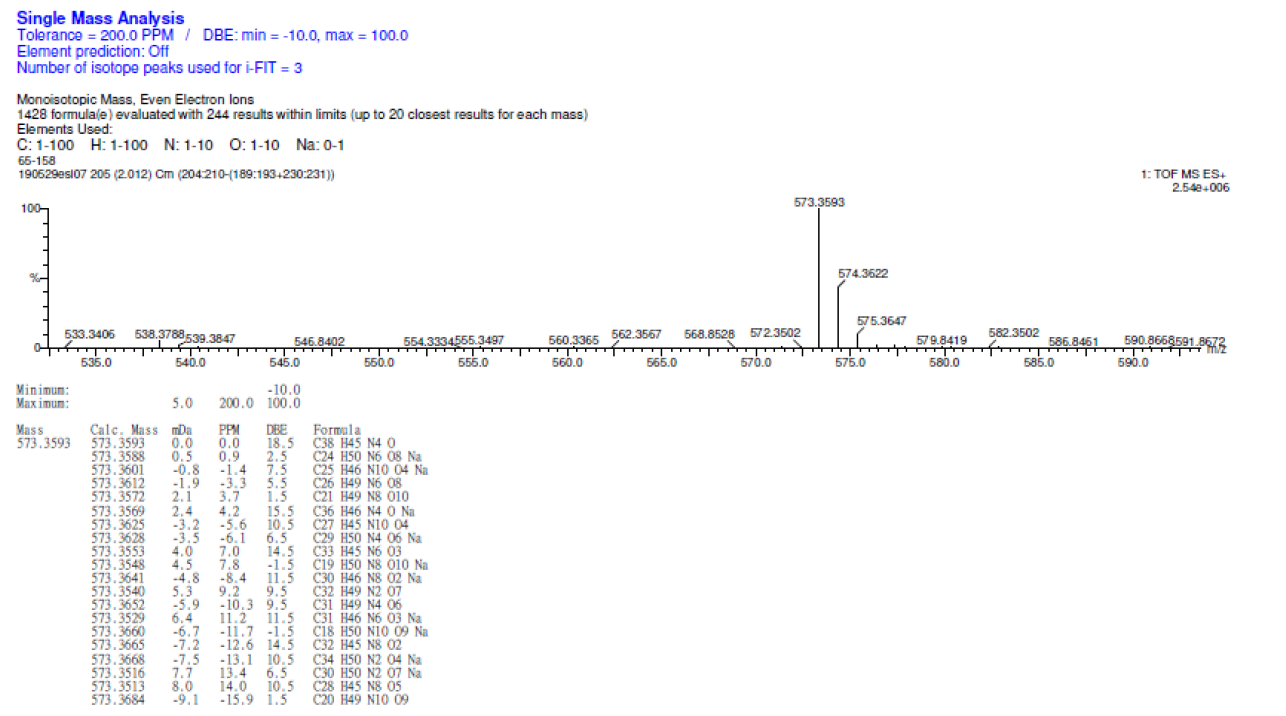


**HRMS Data of Compound (cp6)**


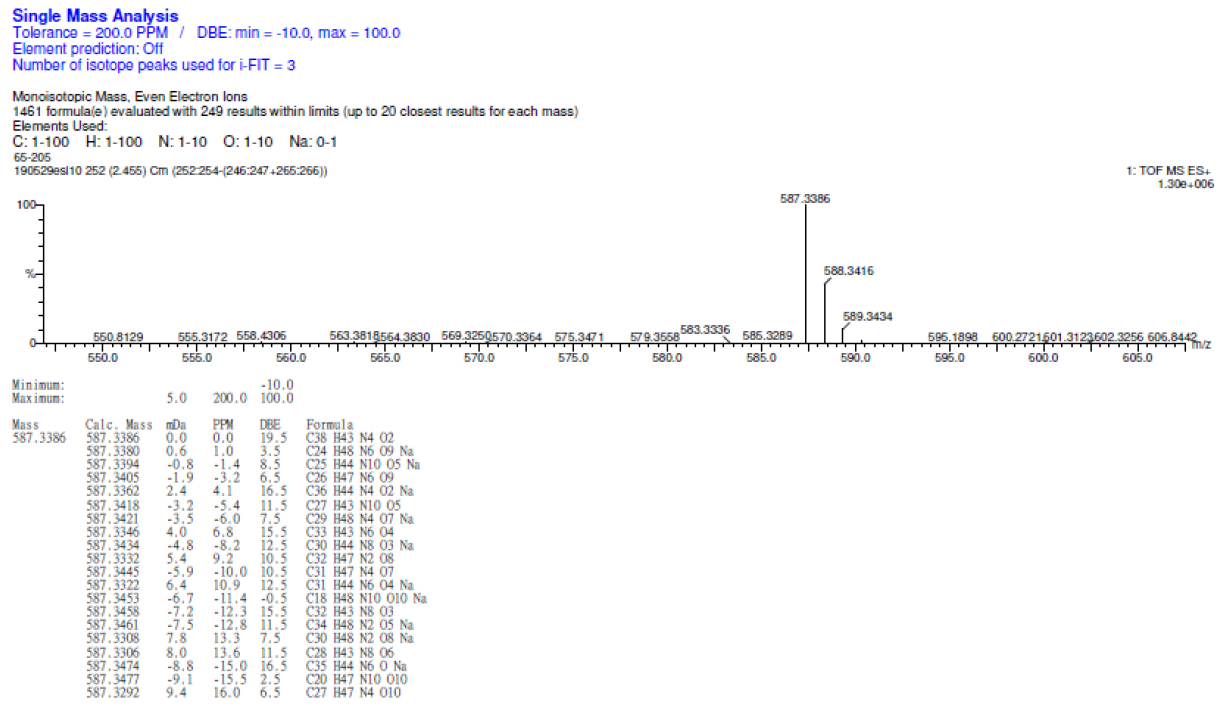


**HRMS Data of Compound (cp7)**


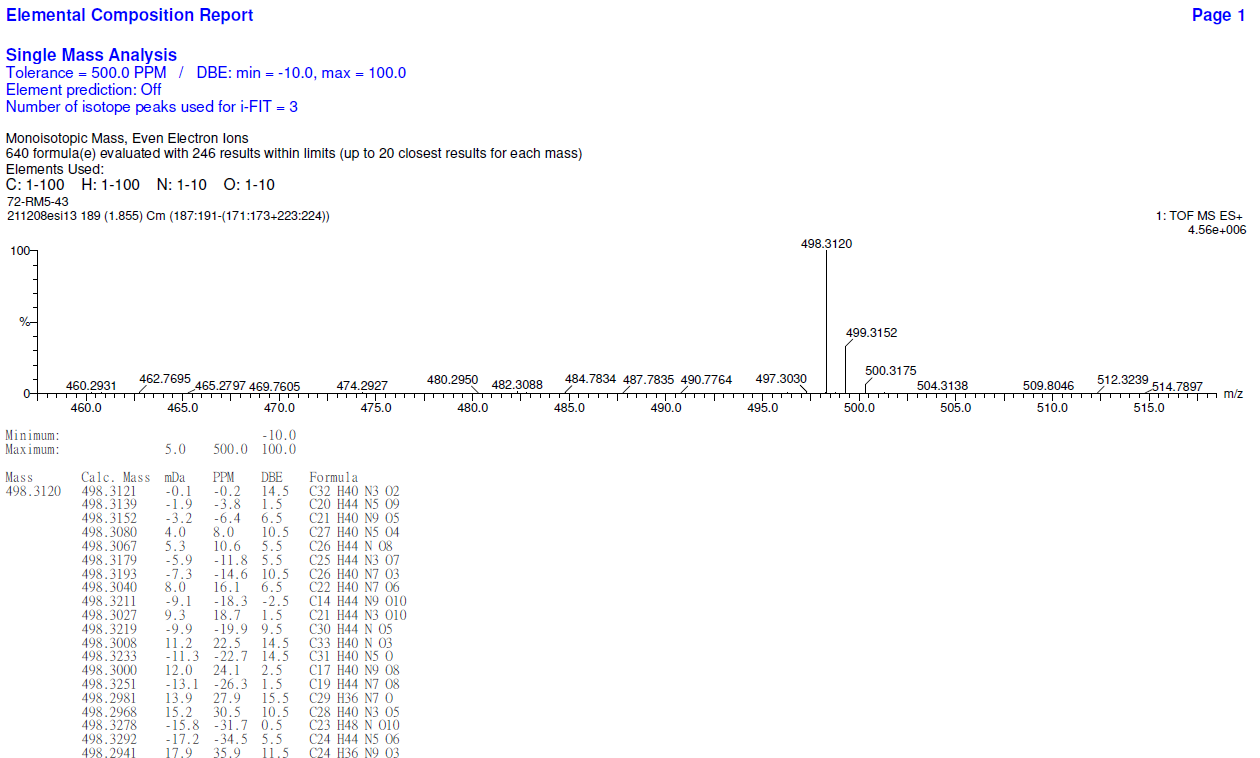


**HRMS Data of Compound (cp8)**


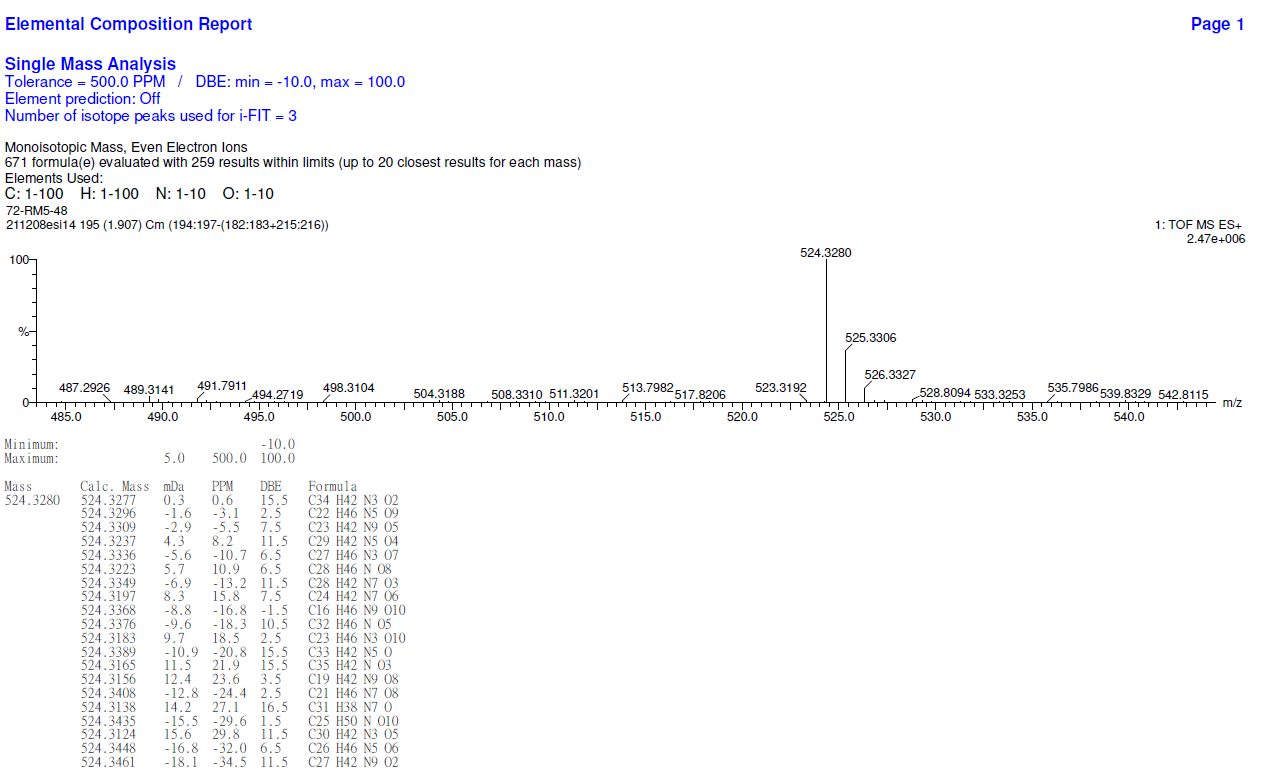


**HRMS Data of Compound (cp9)**


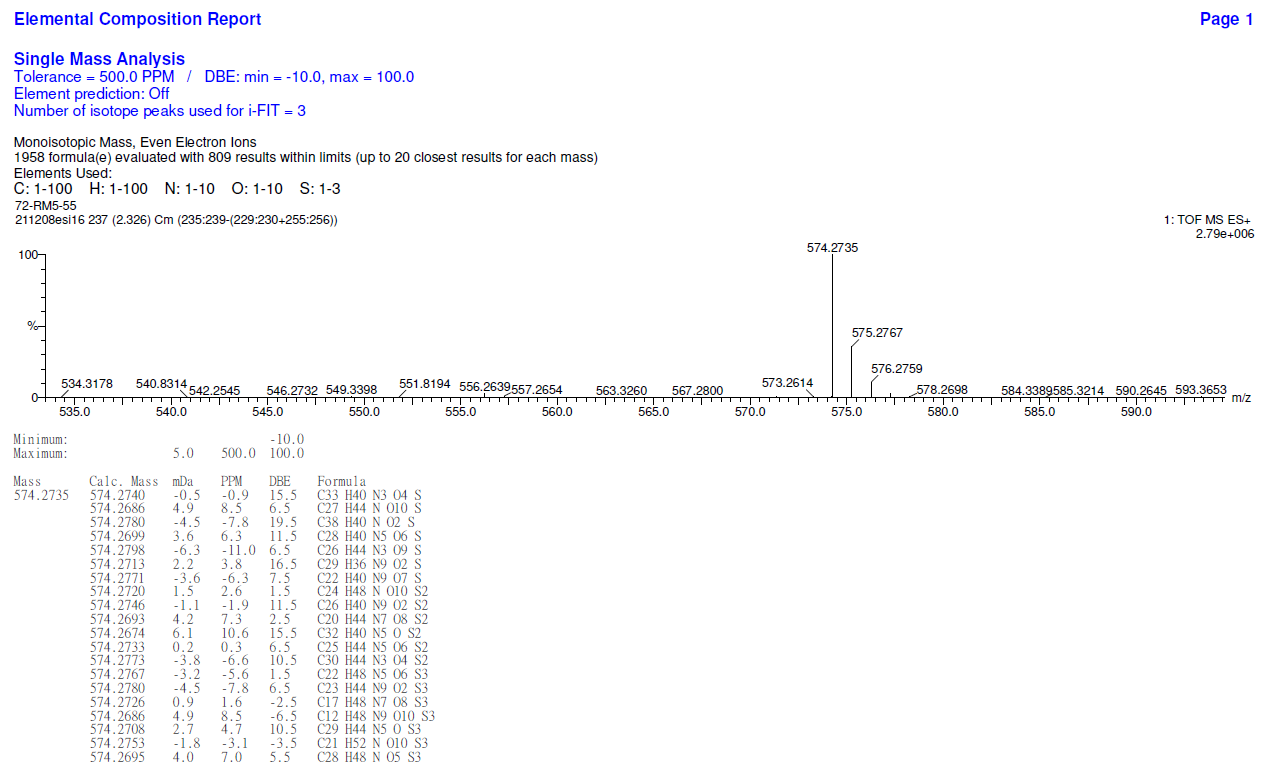


**HRMS Data of Compound (cp10)**


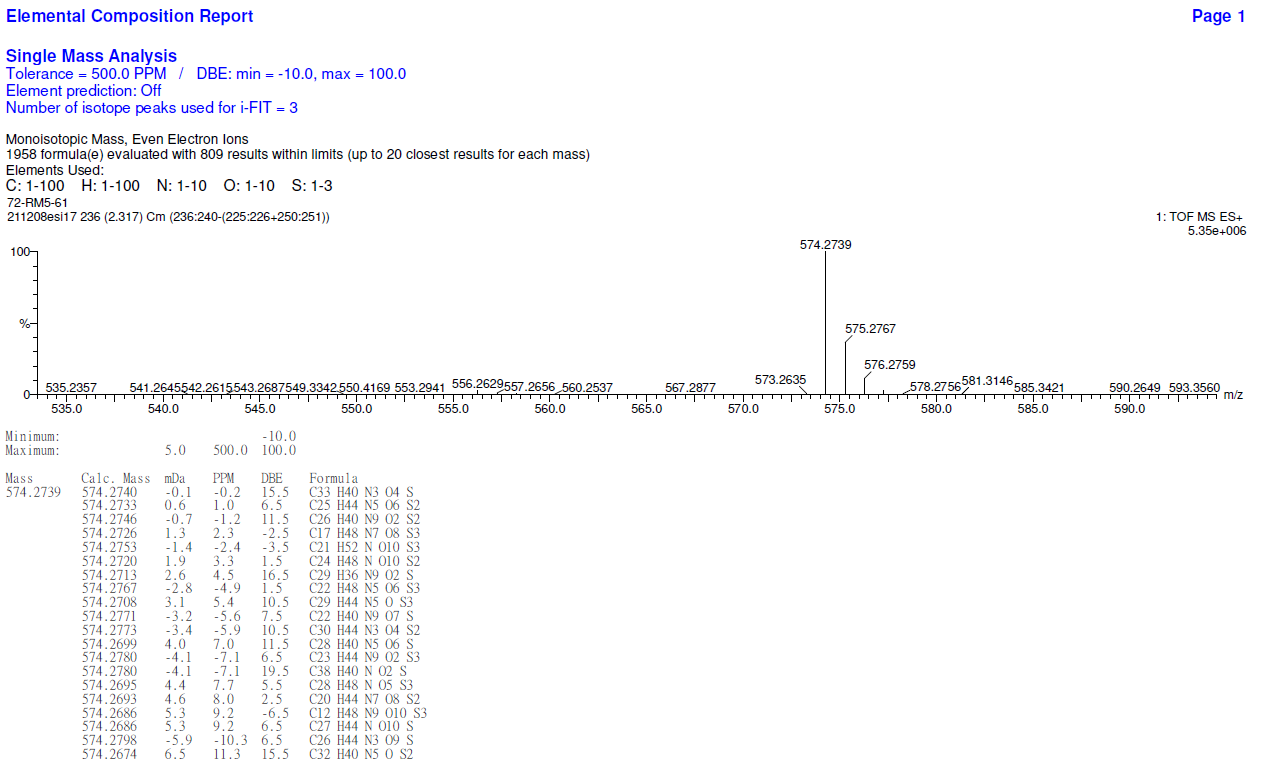


**HPLC Purity Data of Compound (cp1)**


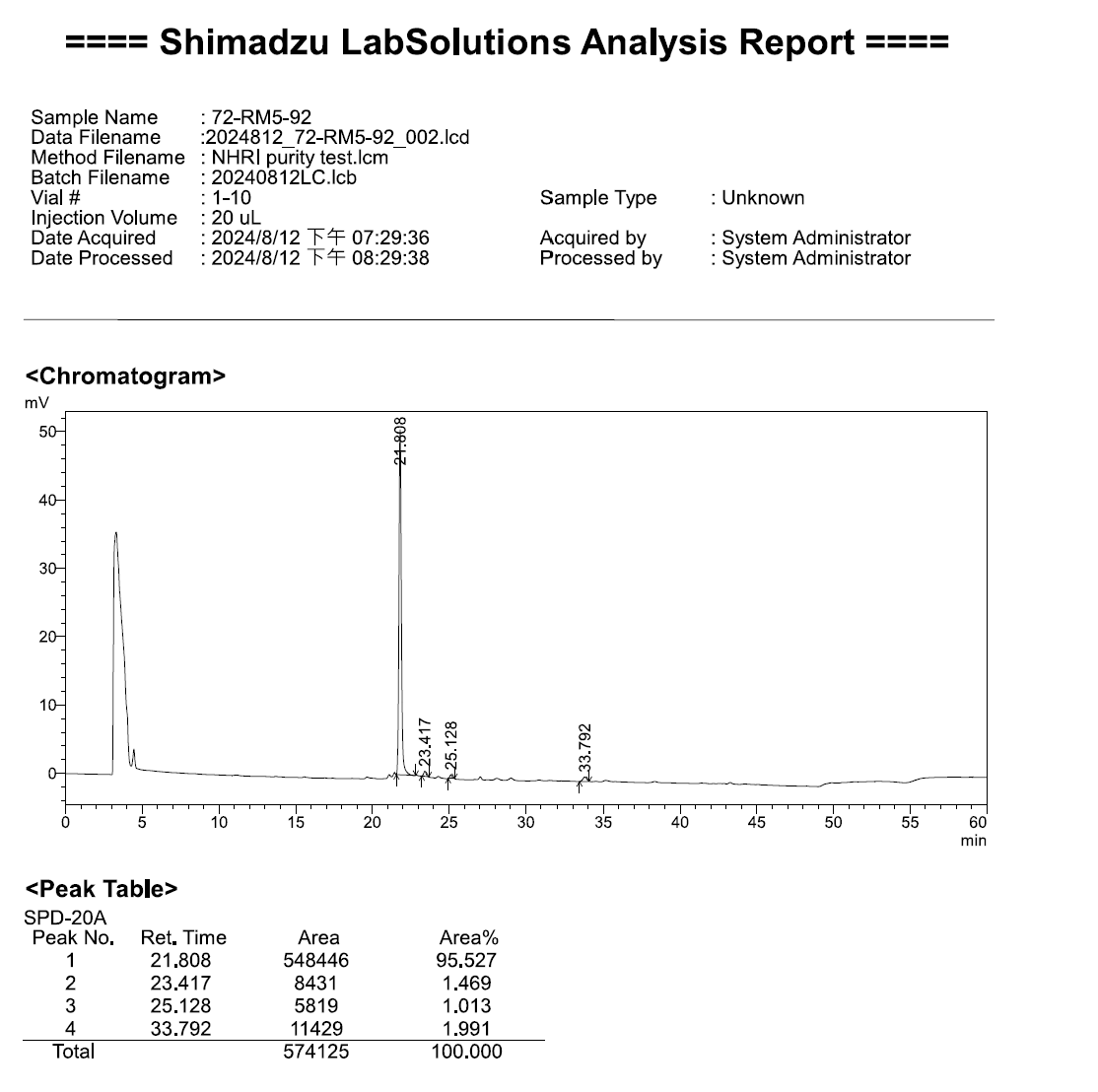


**HPLC Purity Data of Compound (cp2)**


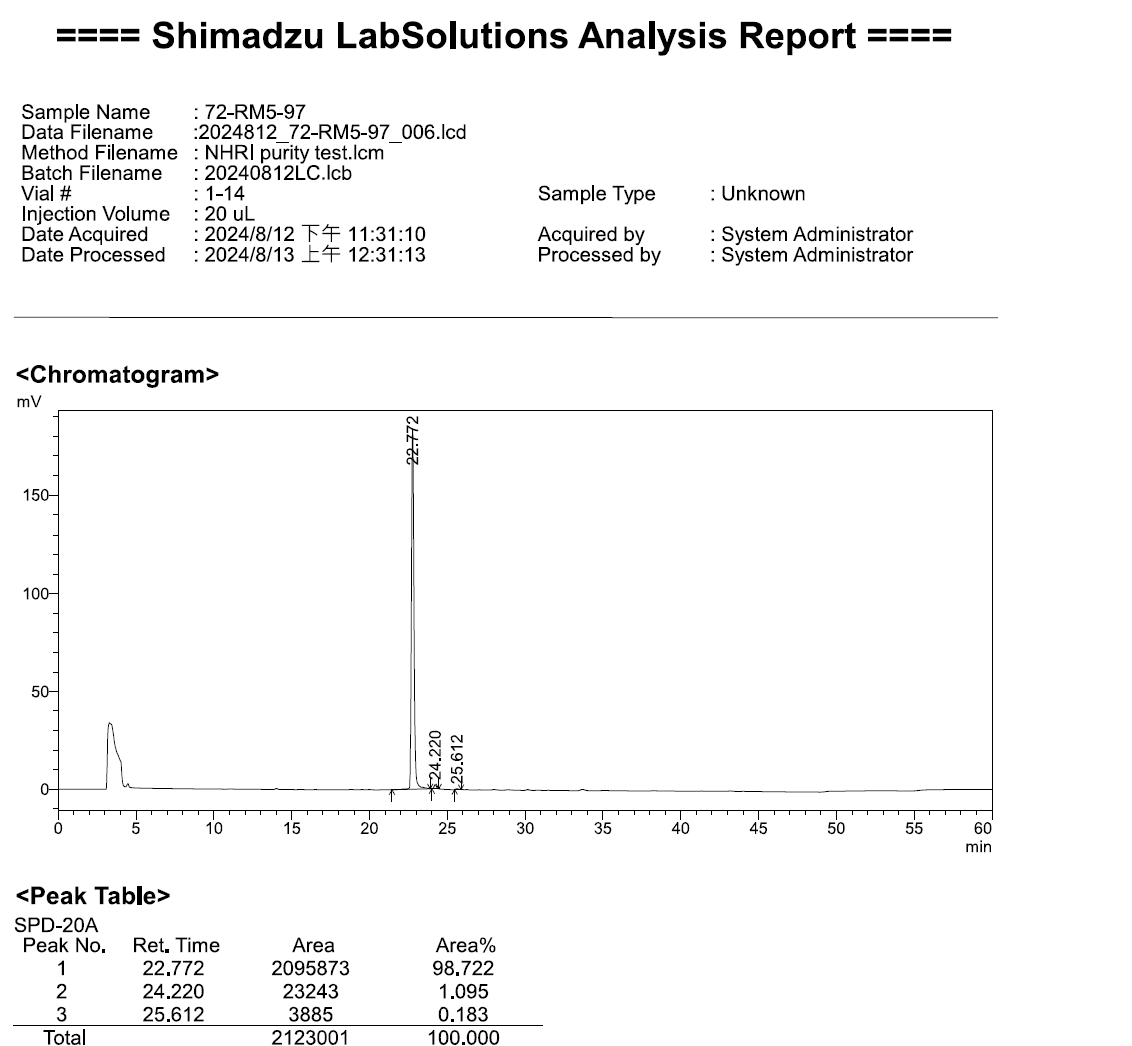


**HPLC Purity Data of Compound (cp3)**


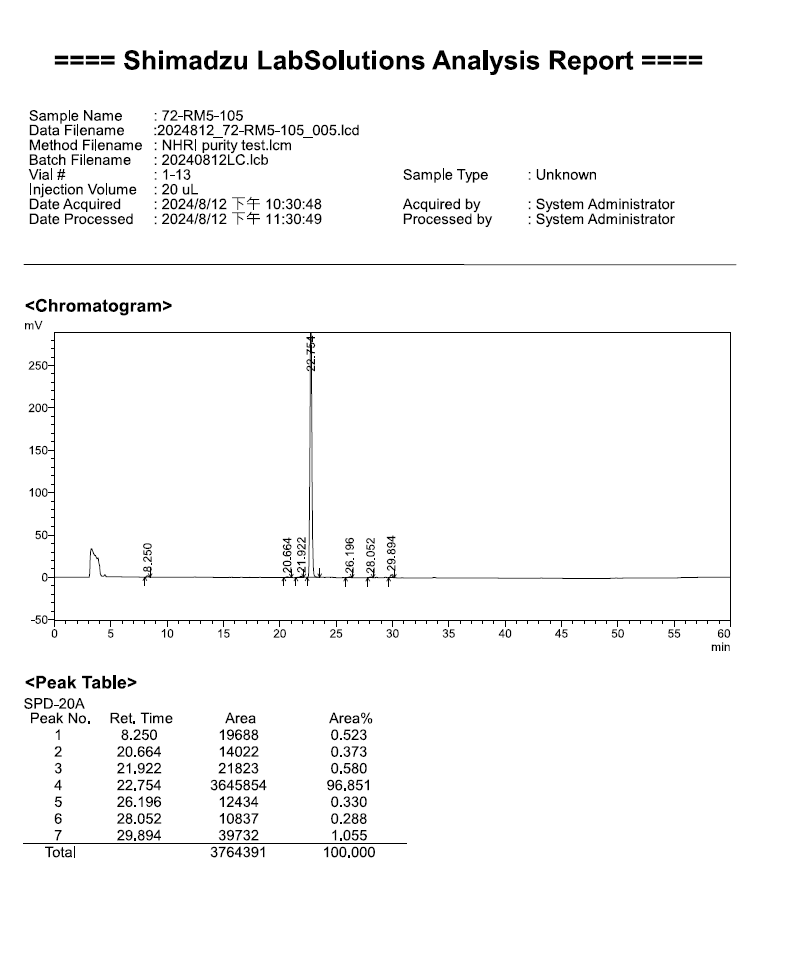


**HPLC Purity Data of Compound (cp4)**


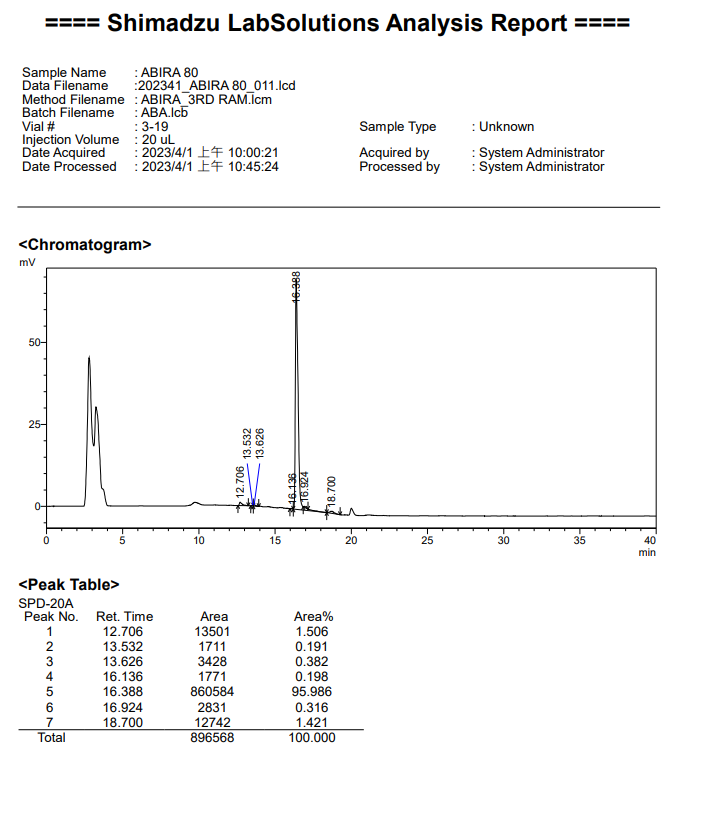


**HPLC Purity Data of Compound (cp5)**


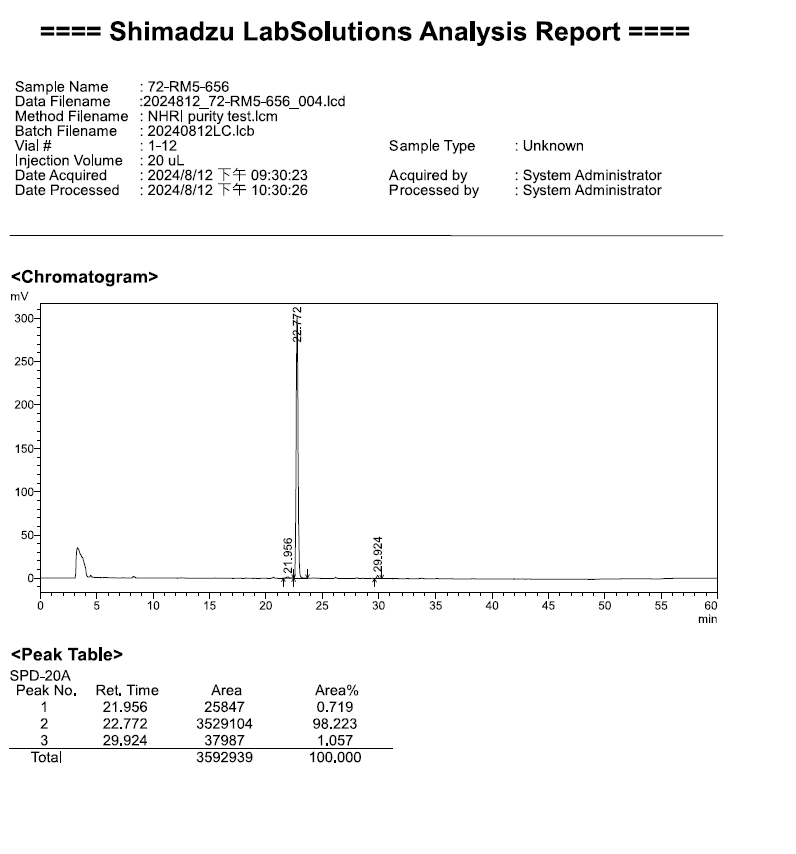


**HPLC Purity Data of Compound (cp6)**


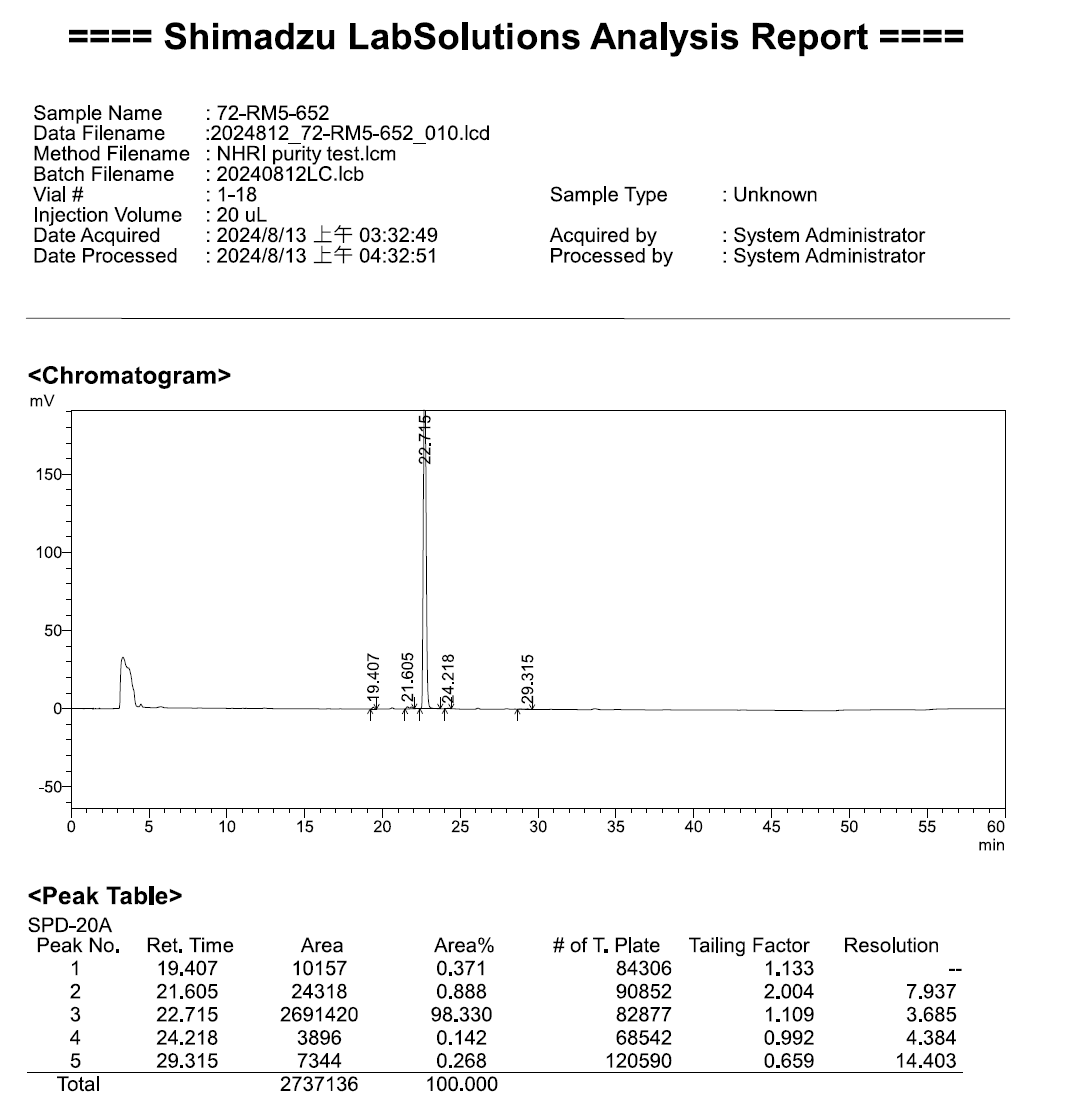


**HPLC Purity Data of Compound (cp7)**


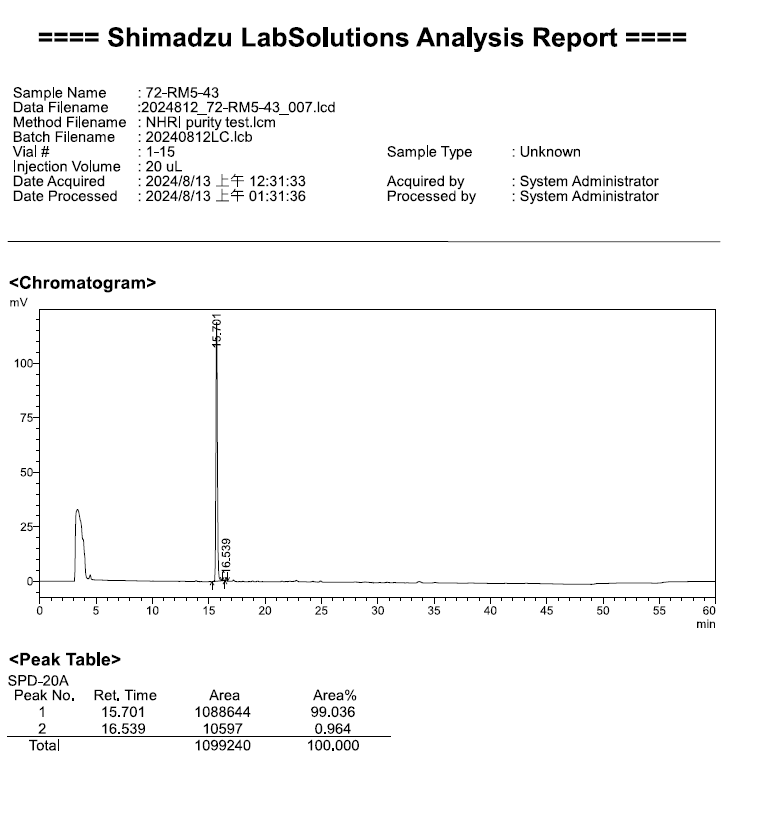


**HPLC Purity Data of Compound (cp8)**


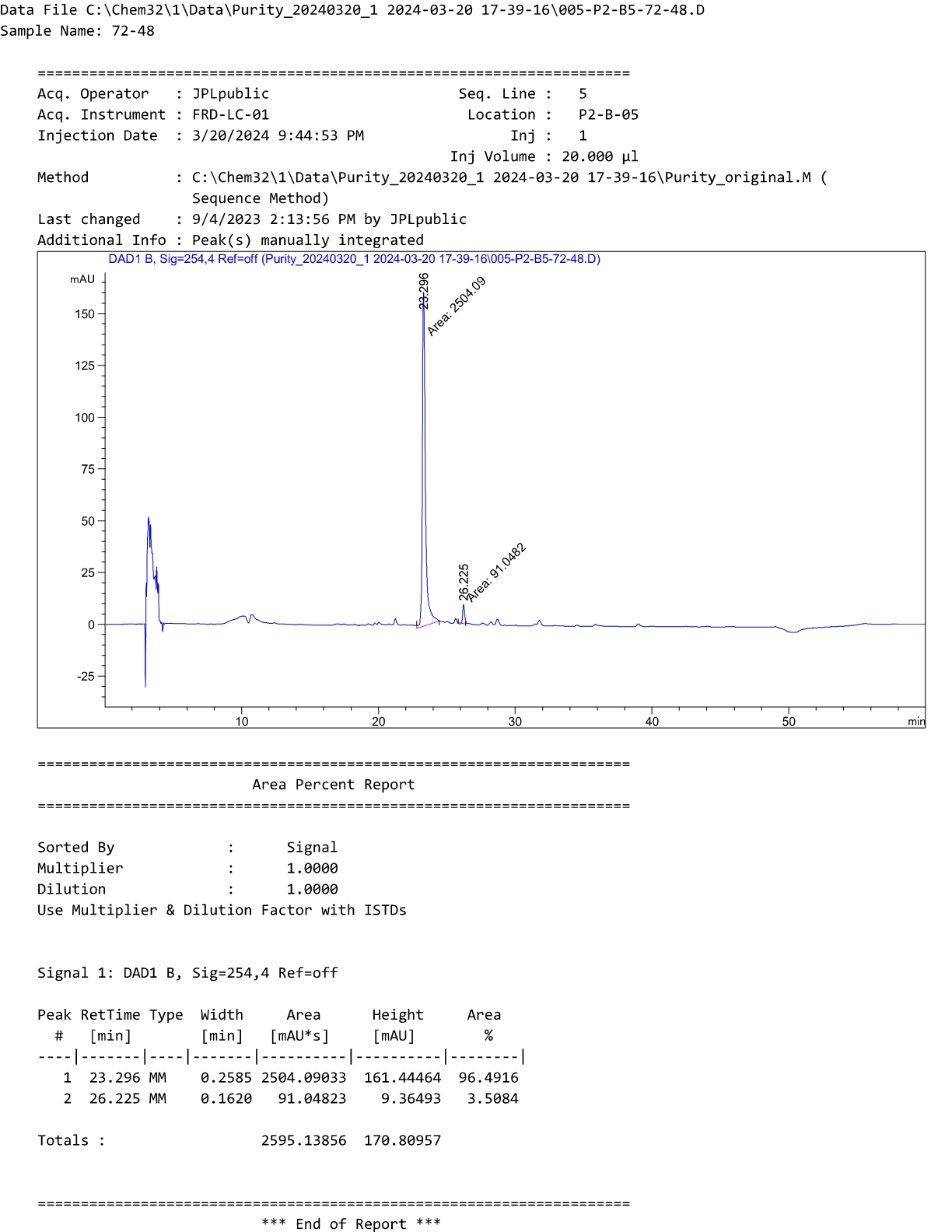


**HPLC Purity Data of Compound (cp9)**


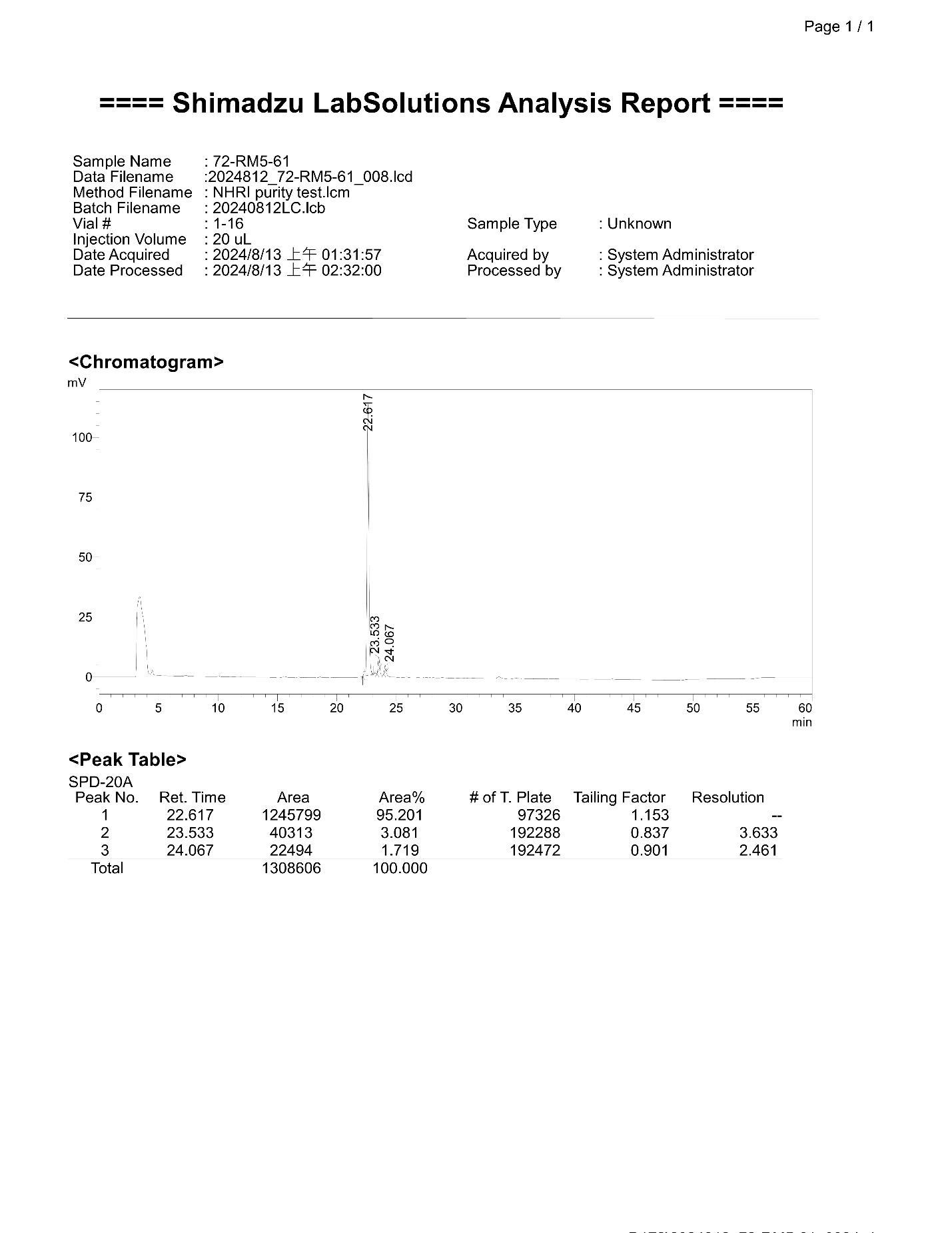


**HPLC Purity Data of Compound (cp10)**


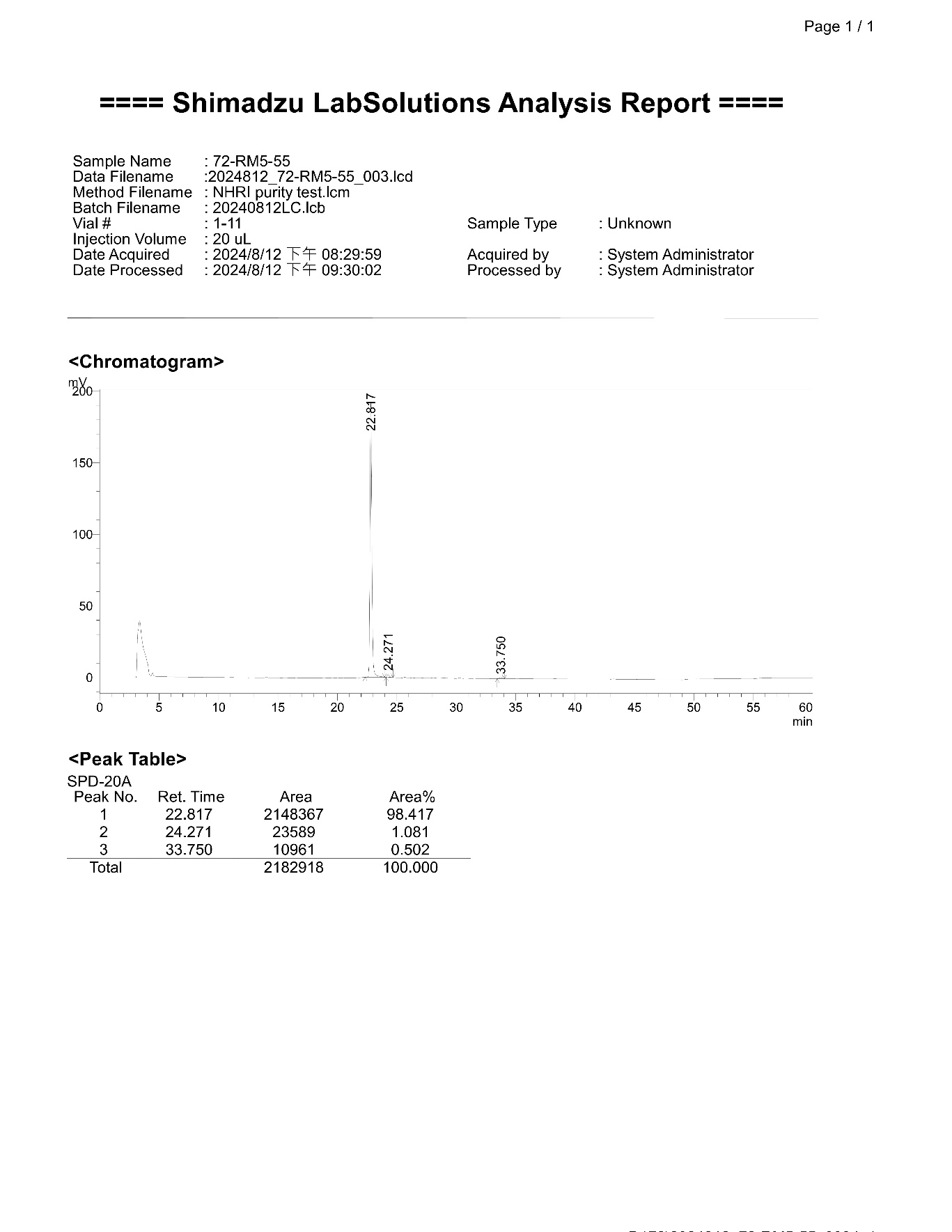

Supplement: Supplementary file 2 — Additional file 2. [file 12929_2026_1241_MOESM2_ESM.docx]
